# Effectiveness and safety of multidrug therapy containing clofazimine for paucibacillary leprosy and clarithromycin for rifampicin-resistant leprosy: a systematic review and meta-analysis

**DOI:** 10.3389/fmed.2023.1139304

**Published:** 2023-05-10

**Authors:** Thais Montezuma, Sebastian Vernal, Elaine Nascimento Andrade, Jurema Guerrieri Brandão, Gustavo Laine Araújo de Oliveira, Ciro Martins Gomes

**Affiliations:** ^1^Health Technology Assessment Unit, Hospital Alemão Oswaldo Cruz, São Paulo, Brazil; ^2^LIM-49, Instituto de Medicina Tropical de São Paulo, Universidade de São Paulo, São Paulo, Brazil; ^3^Coordenação-Geral de Vigilância das Doenças em Eliminação, Ministério da Saúde do Brasil, Brasília, Brazil; ^4^Programa de Pós-Graduação em Ciências Médicas, Universidade de Brasília, Brasília, Brazil; ^5^Programa de Pós-Graduação em Patologia Molecular, Universidade de Brasília, Brasília, Brazil

**Keywords:** leprosy, meta-analysis, drug resistance, microbial, GRADE approach, systematic review

## Abstract

**Introduction:**

The present study aimed to evaluate leprosy cure and relapse rates as primary outcomes related to two additional strategies for leprosy treatment: clofazimine for paucibacillary (PB) leprosy patients and clarithromycin for patients with rifampicin-resistant leprosy.

**Methods:**

We conducted two systematic reviews (protocols CRD42022308272 and CRD42022308260). We searched the PubMed, EMBASE, Web of Science, Scopus, LILACS, Virtual Health Library and Cochrane Library databases, registers of clinical trial databases and gray literature. We included clinical trials evaluating the addition of clofazimine to PB leprosy treatment and the use of clarithromycin for treating patients with rifampicin-resistant leprosy. Risk of bias (RoB) in randomized clinical trials was assessed by the RoB 2 tool and that in non-randomized clinical trials was assessed by the ROBINS-I tool; and the certainty of the evidence was assessed by the Grading of Recommendations Assessment, Development and Evaluation (GRADE) system. A meta-analysis of dichotomous outcomes was performed.

**Results:**

For clofazimine, four studies were included. Cure and relapse rates were not different with the addition of clofazimine to PB leprosy treatment and demonstrated very low certainty of evidence. For clarithromycin, six studies were included. Considerable heterogeneity resulted from the difference between comparators, and studies showed no difference in the assessed outcomes with the addition of clarithromycin to rifampicin-resistant leprosy treatment. Mild adverse events were reported for both drugs but did not significantly impact treatment.

**Discussion:**

The effectiveness of both drugs still needs to be determined. Adding clofazimine to PB leprosy treatment may reduce the repercussions of an incorrect operational classification with no apparent relevant side effects.

**Systematic review registration:**

https://www.crd.york.ac.uk/prospero/display_record.php?ID=CRD42022308272; https://www.crd.york.ac.uk/prospero/display_record.php?ID=CRD42022308260, identifier: CRD42022308272; CRD42022308260.

## 1. Introduction

Leprosy is a chronic infectious granulomatous disease caused by *Mycobacterium leprae* and *Mycobacterium lepromatosis* that predominantly affects the skin and peripheral nerves (1). Regrettably, leprosy is still one of the most neglected diseases worldwide, impacting more than 120 countries, mainly in underdeveloped settings; more than 200 thousand new cases were reported in 2019 (2). Early diagnosis and treatment are crucial for reducing the burden of this disease and avoiding long-term irreversible consequences such as deformities and mutilations (3, 4).

Although leprosy is one of the oldest known diseases of humankind, effective leprosy treatment only began in 1941 with the discovery of sulfone (5, 6). The historical management of leprosy involved compulsory isolation, leading to permanent social stigma (7). Dapsone toxicity has always been a concern, joined by reports of resistance (8, 9). In this scenario, the World Health Organization (WHO) (5) recruited a group of specialists, called “THELEP.” Despite the lack of proper evidence in those days, the problem was too urgent for a solution to be delayed; thus, in 1981, THELEP recommended multidrug therapy (MDT) (5) to solve the dapsone resistance problem and to make shorter treatment periods possible.

**Figure 1:**
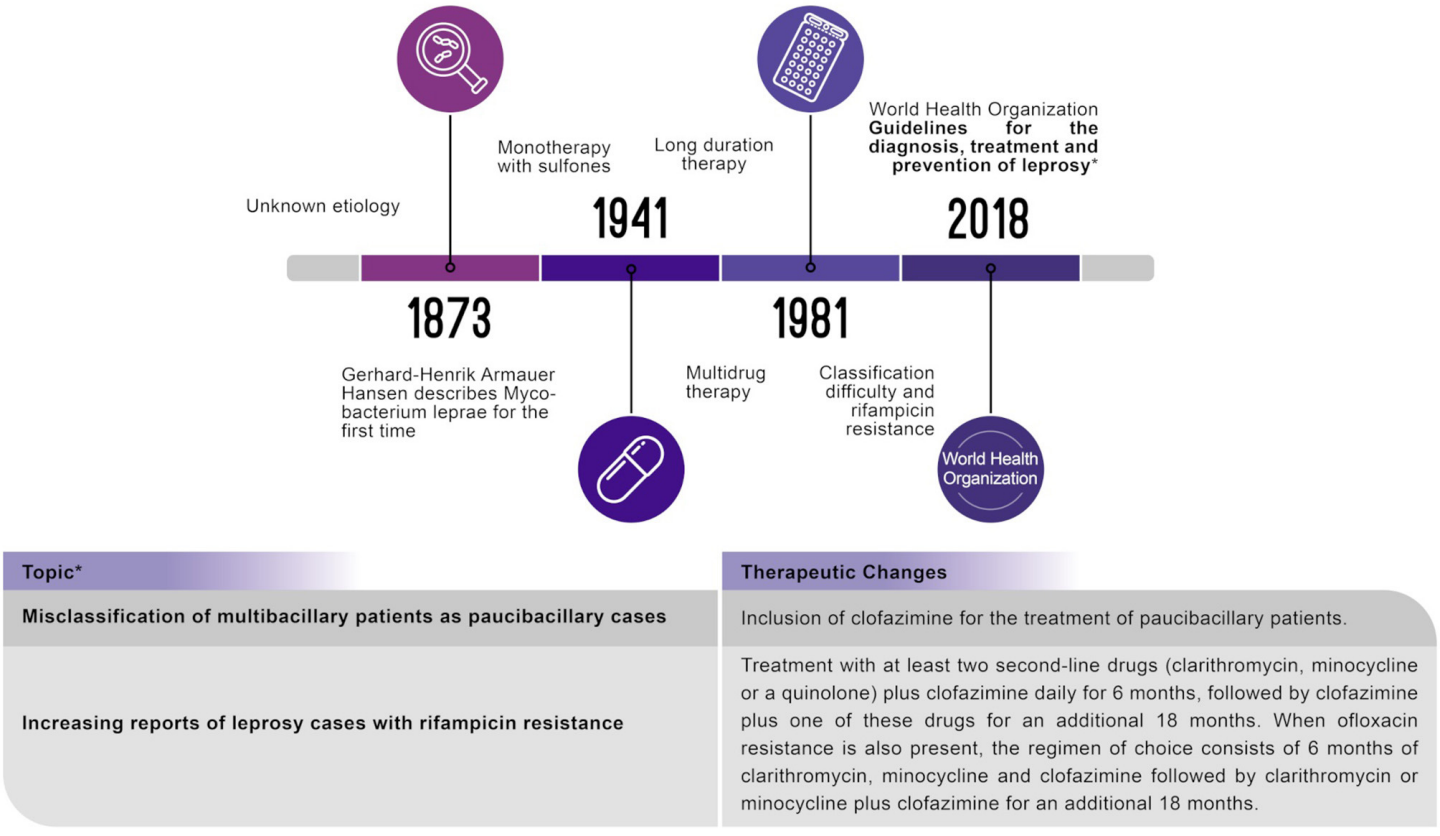
This systematic literature review evaluated clinical pathways and the World Health Organization recommendations.

Although new cases are registered annually, the incidence of leprosy has dramatically reduced since the introduction of MDT; however, leprosy persists in some countries with endemic pockets such as Brazil, India and Indonesia. Despite the success of MDT, new challenges still arise (2, 10). Recent reports of rifampicin-resistant *M*. leprae (11, 12) and the inherent difficulty in properly classifying patients as having the multibacillary (MB) or paucibacillary (PB) forms are also threats to leprosy control (13, 14). Considering the significant gap in the literature, the WHO relies on expert opinions. In 2018, the “Guidelines for the Diagnosis, Treatment and Prevention of Leprosy” (15) recommended the use of clofazimine for patients with PB leprosy and clarithromycin for leprosy cases resistant to rifampicin (Figure 1).

The present study aimed to evaluate leprosy cure and relapse rates as primary outcomes related to two additional strategies for leprosy treatment: (I) clofazimine for PB leprosy patients and (II) clarithromycin for patients with rifampicin-resistant leprosy. In addition, as secondary outcomes, adverse events bacteriological and morphological index reductions, quality of life and treatment adherence were also assessed.

## 2. Methods

### 2.1. Protocol and registration

Two separate review protocols were recorded to analyse the two recent WHO therapeutic recommendations for leprosy treatment. The protocols were registered in the International Prospective Register of Systematic Reviews (PROSPERO): CRD42022308272 (clofazimine review) and CRD42022308260 (clarithromycin review). The reviews strictly followed the recommendations of the Cochrane Handbook for Systematic Reviews of Interventions (16) and were reported following the Reporting Items for Systematic Review and Meta-Analyses (PRISMA) statement (17).

### 2.2. Eligibility criteria

#### 2.2.1. Population, intervention and comparator studies eligible for the clofazimine review

For the clofazimine review, studies targeting individuals of any age diagnosed with PB leprosy that addressed leprosy treatment using clofazimine in combination with dapsone and rifampicin for 6 months (WHO-PB-MDT) were eligible. For eligibility, dapsone and rifampicin must have been used for 6 months in a comparator group.

#### 2.2.2. Population, intervention and comparator studies eligible for the clarithromycin review

For the clarithromycin review, studies targeting individuals of any age diagnosed with PB or MB leprosy that addressed leprosy treatment with the use of clarithromycin alone or in combination with another drug were eligible. One of the following drug combinations must have been used in a comparator group: dapsone, rifampicin, quinolone, minocycline, clofazimine, ofloxacin and/or sparfloxacin. The presence of rifampicin resistance was assessed by subgroup analysis.

#### 2.2.3. Outcomes and study designs eligible for the clofazimine and clarithromycin reviews

Regarding eligibility, all studies must have evaluated at least one of the following outcomes: efficacy/effectiveness (cure and relapse rates and bacteriological and morphological index reductions), safety (any adverse event or serious adverse event), quality of life or treatment adherence. Eligible study designs included randomized clinical trials (RCTs), non-randomized clinical trials, and observational studies with comparator groups (cohort or case–control studies). Systematic reviews, narrative reviews, experimental animal studies, cross-sectional studies, or case reports were excluded. There were no restrictions regarding the study follow-up time, language or year of publication.

### 2.3. Sources of information and search strategy

For both reviews, literature searches were conducted on April 1, 2022, in the PubMed, EMBASE, Web of Science, Scopus, LILACS, Virtual Health Library (BVS) and Cochrane Library databases. PubMed, EMBASE and Cochrane Library alerts were set up to provide a weekly update of new literature until August 13, 2022. A search was performed for ongoing studies in clinicaltrials.gov and the International Clinical Trials Registry Platform (ICTRP). The thesis and dissertation databases were manually checked, and gray literature was accessed in the Opengrey.eu database. The reference lists of the relevant studies were searched by the “backwards snowballing” method (Supplementary Tables 1A, 2A, respectively).

### 2.4. Selection of studies

In both reviews, the references were exported to EPPI-4 (EPPI Centre, London, UK), and duplicates were removed using the automatic tool. The titles and abstracts were screened by two independent reviewers: CG and SV for the clofazimine review and CG and TM for the clarithromycin review. Disagreements were evaluated by a third reviewer: TM or SV. The full texts of the selected studies were evaluated in the same way.

### 2.5. Data collection process

Data were extracted using a standardized form developed by a leprosy specialist (CG). Two reviewers extracted the data independently, and disagreements were resolved through consensus: GC + TM for the clofazimine review and CG + SV for the clarithromycin review. The extracted information is disclosed in Supplementary material B.

### 2.6. Risk of bias assessment of the included studies

The risk of bias of RCTs was assessed at the outcome level using the Cochrane 2.0 Risk of Bias tool (RoB 2), and that of non-randomized clinical trials was evaluated using the Risk of Bias In Non-randomized Studies of Interventions (ROBINS-I) tool by two independent evaluators with subsequent consensus (CG and TM).

### 2.7. Data analysis

Effect sizes are presented as relative risks (RR) for dichotomous outcomes and by the mean difference (MD) for continuous outcomes. Meta-analysis of the dichotomous outcomes was performed using a random-effects model with the Mantel–Haenszel method in Review Manager software version 5.4 (The Cochrane Collaboration, 2020) if at least two comparable studies were identified. Heterogeneity was verified by forest graphs, chi-squared values (*p* < 0.05) and *I*^2^ statistics (>50%). Regression models were used to assess publication bias if at least ten studies were included.

### 2.8. Analysis of certainty in the final set of evidence

The certainty in the set of evidence was analyzed using the Grading of Recommendations Assessment, Development and Evaluation (GRADE) system (18).

## 3. Results

### 3.1. Results of the clofazimine review

#### 3.1.1. Selected studies (clofazimine review)

The database search resulted in 8,841 references (11 RCT registers and 8,830 records identified in the databases, with 4,796 duplicate records removed), and 4,045 titles and abstracts were screened. Ultimately, 65 full texts were analyzed, and four studies were included in the review [eight publications (19–26), with four included in the main study by de Sá Gonçalves et al. (22) identified as a clinical trial for uniform multidrug therapy for leprosy patients in Brazil - U-MDT/CT-BR (21, 24–26)]. The flow chart of the selected studies (Supplementary Figure 1A) and the excluded studies, including the reasons for exclusion (Supplementary Table 3A), are shown in Supplementary material A.

#### 3.1.2. Characteristics of the included studies (clofazimine review)

Three RCTs (19, 20, 22) and one non-RCT (23) were included; three were conducted in India, and one was conducted in Brazil. Four hundred sixty-four participants were included (the study sample size ranged from 40 to 300 patients with PB leprosy) (Table 1). In all studies, the diagnosis followed the criteria recommended by the WHO.

**Table 1:** Characteristics of the included studies evaluating the use of clofazimine in paucibacillary leprosy treatment.

**References**	**Study design**	**Country**	**Follow-up time (years)**	**N**	Age (years)^*^	**Men (*N*)**	**Intervention**	**Comparator**	**Outcomes evaluated**
Bhate et al. (19)	RCT	India	1	80	19 to 45	80	600 mg rifampicin monthly + 100 mg clofazimine every 2 days + 100 mg dapsone daily for 6 months; followed by dapsone monotherapy for 1 year	600 mg rifampicin monthly + 100 mg dapsone daily for 6 months, followed by dapsone monotherapy for 1 year	Cure rate, AEs
de Sá Gonçalves et al. (22)	RCT	Brazil	6	40	6 to 65	NR	WHO-PB-MDT	WHO-PB-MDT without clofazimine	AEs, treatment adherence
Katoch et al. (20)	RCT	India	3.5	300	NR	240	WHO-PB-MDT	WHO-PB-MDT without clofazimine + placebo	Cure rate, relapse rate, AEs
Prasad et al. (23)	CT	India	1	44	10 to 72	32	Rifampicin, clofazimine and dapsone	Rifampicin and dapsone	Cure rate, AEs

RCT, randomized clinical trial; CT, clinical trial; AEs, adverse events; NR, not reported.

* Age is presented as the minimum and maximum age.

#### 3.1.3. Risk of bias (clofazimine review)

The studies by Bhate et al. (19) and Katoch et al. (20) were classified as having a high RoB for the cure outcome. The study by Katoch et al. (20) was classified as having a high RoB for the relapse outcome. These studies were evaluated by the RoB 2 tool. The study by Prasad et al. (23) was evaluated by the ROBINS-I tool and was classified as having a serious RoB for the cure outcome. The RoB analysis details are provided in Supplementary material B.

#### 3.1.4. Primary outcomes (clofazimine review)

**Figure 2:**
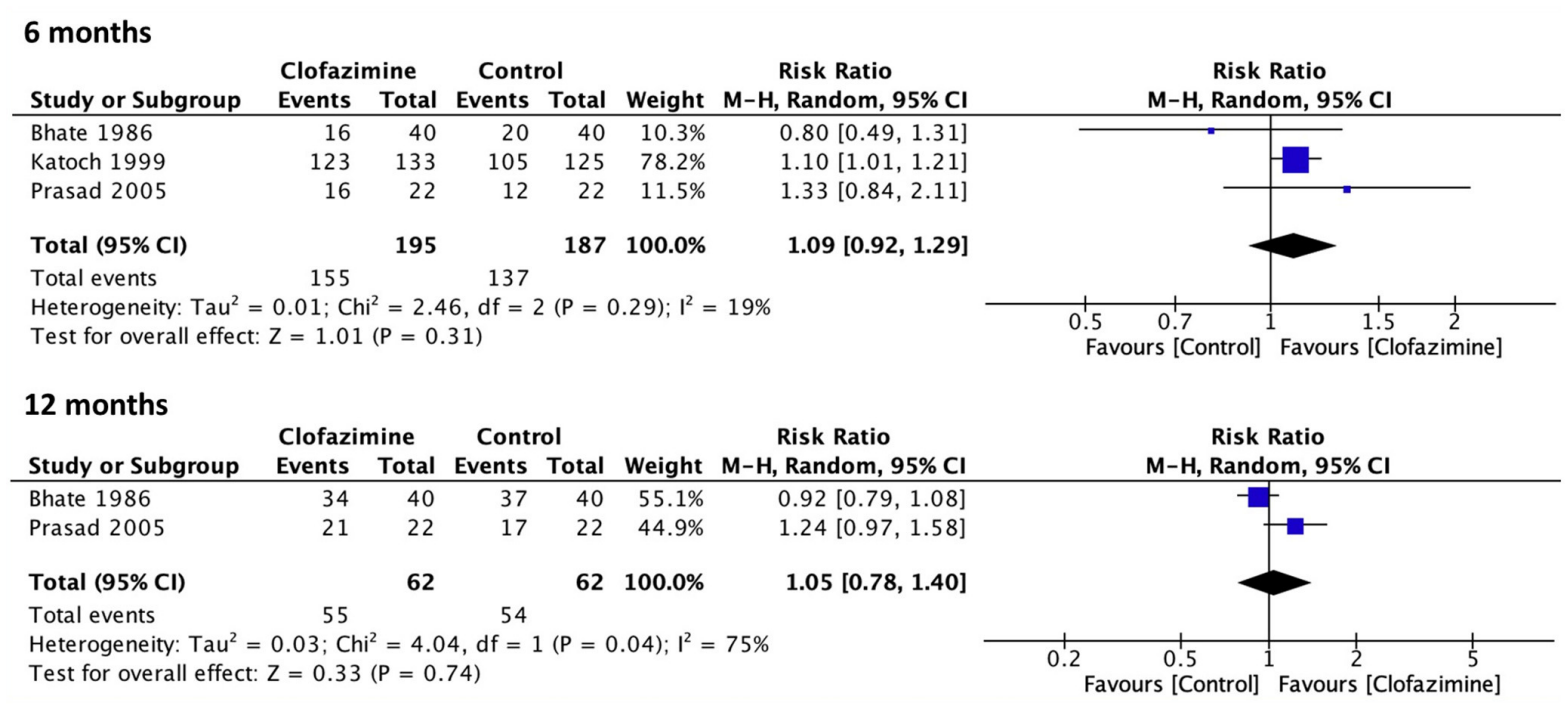
Meta-analysis of the studies evaluating clinical cure events comparing World Health Organization paucibacillary multidrug therapy for leprosy with (clofazimine) or without clofazimine (control) at the 6 and 12 month follow-ups.

The cure outcome (clinical inactivity) was assessed in three studies (19, 20, 23) with a 6 month follow-up. The summary effect of the treatment using clofazimine for PB leprosy showed an RR of 1.09 (95% CI: 0.92 to 1.29) compared to that of the control treatment (dapsone and rifampicin). No significant heterogeneity was found. Two studies evaluated the cure outcome within a 12 month follow-up period (19, 23). The summary effect showed an RR of 1.05 (95% CI: 0.78 to 1.40). There was significant heterogeneity among the studies (*P-*value = 0.04; *I*^2^ = 75%) (Figure 2). Relapse was assessed in only one study (20) at a follow-up time between 2.5 and 3.5 years, with an RR of 0.20 (95% CI: 0.01 to 4.13).

#### 3.1.5. GRADE approach (clofazimine review)

The certainty of the body of evidence was evaluated for the cure outcome and classified as having very low certainty. It was impossible to assess publication bias due to the small number of studies. In the same way, for the relapse outcome, it was impossible to assess inconsistency because only one study was included (Supplementary material B).

### 3.2. Results of the clarithromycin review

#### 3.2.1. Selected studies (clarithromycin review)

The initial screening resulted in 2,133 references (two RCT registers and 2,131 records identified in databases, with 632 duplicate records removed), and 1,501 titles and abstracts were screened. Eleven full texts were evaluated, and six studies were included (27–32). The flow chart of the selected studies (Supplementary Figure 2A) and the excluded studies, including the reasons for exclusion (Supplementary Table 4A), are shown in Supplementary material A.

#### 3.2.2. Characteristics of the included studies (clarithromycin review)

A total of 456 participants were included in six RCTs (27–32), and the sample size ranged from 14 to 300 individuals with MB leprosy. In the study by Ji et al. (29, 30), leprosy was diagnosed through skin smears, and in the other four included studies, the diagnosis was defined following the WHO's recommendations [included individuals with positive acid-fast bacilli and more than ten skin lesions (31)].

#### 3.2.3. Risk of bias (clarithromycin review)

The study by Girdhar et al. (27) was classified as having a high risk of general bias, and the study by Ji et al. (29, 30) was assessed as having some concerns for the “cure” outcome. The study by Girdhar et al. (27) was also classified as having a high RoB for the relapse outcome. The RoB analysis details are provided in Supplementary material B.

#### 3.2.4. Primary outcomes (clarithromycin review)

It was impossible to perform a meta-analysis of any of the outcomes due to the heterogeneity of the intervention arms and follow-up times of the included studies. The characteristics of the individual studies are presented in Table 2. The difference between comparators and the variety of associations made this a complex analysis. Only one study compared a modified MB-MDT substituting 600 mg of rifampicin per month with 2 g of clarithromycin per month vs. a classic WHO MB-MDT at 3 months after the start of therapy (28). Analyses of the cure and relapse outcomes of the included studies are presented in Tables 3, 4, respectively. Data on the cure and relapse outcomes at later times were not available.

**Table 2:** Characteristics of the included studies evaluating the use of clarithromycin in leprosy treatment.

**References**	**Study design**	**Country**	**Follow-up time**	**N**	Age (years)^*^	**Men (N)**	**Intervention**	**Comparator**	**Outcomes evaluated**
Girdhar et al. (27) Adults	RCT	India	24 months	300	30.9 (16.2)	123	500 mg single dose clarithromycin + 600 mg rifampicin + 200 mg ofloxacin + 100 mg minocycline	600 mg single dose rifampicin + 200 mg ofloxacin + 100 mg minocycline	Cure rate, relapse rate
Girdhar et al. (27) Children							250 mg single dose clarithromycin + 300 mg rifampicin + 200 mg ofloxacin + 100 mg minocycline	300 mg rifampicin + 200 mg ofloxacin + 100 mg minocycline	
Gunawan et al. (28)	RCT	Indonesia	3 months	14	NR	11	2 g clarithromycin monthly + 300 mg de clofazimine; 100 mg dapsone daily + 50 mg clofazimine for 3 months	WHO-MB-MDT for 3 months	AEs, bacteriological and morphological index reductions
Ji et al. (30)	RCT	Mali	56 days	36	31 (9.0)	28	500 mg clarithromycin daily + 100 mg minocycline for 56 days	Comparator 1: 500 mg clarithromycin daily for 56 days Comparator 2: 100 mg minocycline daily for 56 days	Cure rate, bacteriological and morphological index reductions
Ji et al. (29)	RCT	Mali	31 days	50	31.3 (10.5)	37	2 g clarithromycin + 200 mg minocycline single doses on the 1st day + placebo daily for 30 days	Comparator 1: 2 g clarithromycin + 200 mg of minocycline + 800 mg ofloxacin single doses on the 1st day and followed placebo daily for 30 days Comparator 2: 600 mg rifampicin + 300 mg clofazimine single doses on the 1st day + 100 mg dapsone daily + 50 mg de clofazimine for 30 days Comparator 3: 600 mg rifampicin single dose on the first day + placebo daily for 30 days Comparator 4: 300 mg clofazimine single dose on the 1st day + 100 mg dapsone daily + 50 mg clofazimine for 30 days	Cure rate, AEs, bacteriological and morphological index reductions
Tejasvi et al. (31)	RCT	India	48 weeks	30	NR	28	500 mg clarithromycin + 600 mg rifampicin, + 200 mg sparfloxacin + 100 mg minocycline daily for 12 weeks	WHO-MB-MDT for 12 months	AEs, bacteriological and morphological index reductions
Wongdjaja et al. (32)	RCT	Indonesia	12 weeks	26	34.5 (11.56)	19	500 mg clarithromycin daily + 600 mg rifampicin + 400 mg ofloxacin 3x/week for 12 weeks	WHO-MB-MDT for 12 weeks	AEs, bacteriological and morphological index reductions

RCT, randomized clinical trial; AEs, adverse events; NR, not reported.

* Age is presented as the mean (standard deviation).

**Table 3:** Outcome analysis of the inclusion of clarithromycin in multibacillary leprosy treatment in the included studies.

**References**	**Follow-up**	**Intervention**	***N* cure**	***N* total**	**Comparator**	***N* cure**	***N* total**	RR (95% CI)^*^	**Effect direction**
Girdhar et al. (27)	6 months	Clarithromycin, rifampicin, ofloxacin and minocycline	117	149	Rifampicin, ofloxacin and minocycline	110	151	1.08 (0.95–1.23)	No difference
	12 months		133	149		135	151	1.00 (0.92–1.08)	
	18 months		133	145		140	148	0.97 (0.91–1.03)	
	24 months		128	140		126	135	0.98 (0.92–1.05)	
Ji et al. (30)	56 days	Clarithromycin and minocycline	11	11	Clarithromycin	12	12	1.00 (0.85–1.17)	No difference
					Minocycline	11	11	1.00 (0.85–1.18)	
Ji et al. (29)	30 days	Clarithromycin and minocycline, followed by a placebo	3	10	Clarithromycin, minocycline and ofloxacin, followed by placebo	2	10	1.50 (0.32–7.14)	No difference
					Rifampicin, clofazimine and dapsone	9	9	0.33 (0.14–0.80)	Favored the comparator
					Rifampicin followed by a placebo	10	10	0.33 (0.14–0.80)	Favored the comparator
					Dapsone and clofazimine	4	10	0.75 (0.22–2.52)	No difference

* RR (95% CI): Relative risk (95% confidence interval), measured by Review Manager version 5.4 software.

**Table 4:** Relapse outcome analysis of the inclusion of clarithromycin in multibacillary leprosy treatment in the Girdhar et al. (27) study.

**References**	**Follow-up**	**Intervention**	***N* relapse**	***N* total**	**Comparator**	***N* relapse**	***N* total**	RR (95% CI)^*^	**Effect direction**
Girdhar et al. (27)	6 months	Clarithromycin, rifampicin, ofloxacin and minocycline	0	149	Rifampicin, ofloxacin and minocycline	0	151	Not estimable	No difference
	12 months		1	149		2	151	0.51 (0.05–5.53)	
	18 months		1	145		0	148	3.06 (0.13–74.55)	
	24 months		0	140		1	135	0.96 (0.06–15.26)	

* RR (95% CI): Relative risk (95% confidence interval), measured by Review Manager version 5.4 software.

#### 3.2.5. Analysis of certainty in the final set of evidence (clarithromycin review)

Regarding the heterogeneity of the studies, an analysis of the certainty of the evidence was performed, including the individual outcomes of each study, and the set was classified as having very low or low certainty of evidence (Supplementary material B). Downgraded domains of inconsistency and publication bias could not be evaluated.

### 3.3. Additional outcomes (clofazimine review and clarithromycin review)

Other outcomes, including bacteriological and morphological index reductions, were appraised for both reviews. Various adverse events were reported; nevertheless, these incidents were usually mild and did not significantly impact treatment feasibility. A detailed description of the other secondary outcomes is provided in Supplementary material B.

## 4. Discussion

The clofazimine and clarithromycin reviews showed no difference in the outcomes with the addition of clofazimine in PB leprosy treatment and the addition of clarithromycin in rifampicin-resistant leprosy treatment. The studies had methodological limitations, and the certainty of the evidence was very low. Thus, there is uncertainty about the new WHO recommendations for leprosy treatment.

Early diagnosis and treatment are among the most critical actions for leprosy control (8, 33). Treatment success depends on proper prescription of PB- or MB-MDT for 6 or 12 months, respectively, with a further distinction between adults and children. In addition, promoting, supervising and guaranteeing treatment adherence and preventing further reinfection, especially through the systematic assessment and follow-up of household contacts, are also crucial for leprosy control. At the end of MDT, clinical and bacilloscopic results are difficult to interpret since patients' reactional states can clinically worsen and bacillus depuration can be slow. Facing these difficulties, the determination of disease persistence or relapse becomes a challenging task for general physicians, making it impossible to rule out treatment failure and making further investigation of antimicrobial resistance mandatory (34).

The long-term MDT duration and the absence of precise criteria for cure evaluation reinforce that the treatment and follow-up of leprosy patients cannot be separated. Owing to the urgency to provide more effective and accessible therapies, in 2018, the WHO recommended the inclusion of clofazimine for PB patients and the inclusion of clarithromycin for patients with rifampicin resistance.

Considering the addition of clofazimine in PB leprosy treatment, the meta-analysis showed no significant difference in the clinical cure rates compared to the control treatment (dapsone and rifampicin) at the 6 and 12 month follow-ups. However, studies were considerably heterogeneous at the second time point. Only one study evaluated relapse (20) rates and showed that after 3.5 years of treatment, the inclusion of clofazimine in MDT for PB leprosy treatment was not different. Other outcomes, including adverse events, treatment adherence and patient satisfaction, were not different between the two types of PB-MDTs (Supplementary material B), and concerns regarding skin discolouration with clofazimine were discarded.

The inclusion of clofazimine has raised controversies and increased the costs of PB leprosy treatment (35). Although different treatment types for PB and MB leprosy still exist, the more similar the drugs are, the smaller the chance of relapse in MB leprosy patients wrongly identified as having PB leprosy. Moreover, the treatment scheme simplification facilitated the logistic distribution since only two types of blister drug packs (adults and children) were needed. Studies that tested a 6 month MB-MDT showed that the cure rate is still relevant in some cases (36), although these results should be interpreted carefully. Indeed, it seems that the incorporation of clofazimine into PB-MDT is safe and may mitigate possible relapses resulting from the sometimes tricky differentiation between PB and MB leprosy (37).

The inclusion of clarithromycin in the leprosy treatment arsenal is an interesting option once this drug is proven to be effective against mycobacteria (29, 38, 39). Even though this alternative focuses on antimicrobial resistance to rifampicin, it can also be an option for WHO-MDT in terms of adverse reactions and drug interactions. Unfortunately, most studies have associated this macrolide with rifampicin, meaning that the role of clarithromycin as a possible replacement for rifampicin still needs to be determined. No differences in the assessed outcomes were observed in the various types of multidrug combinations with clarithromycin. The only study that compared clarithromycin with clofazimine and dapsone vs. WHO-MB-MDT showed no difference in the reduction in the bacteriological index after 3 months of therapy (28). However, only a few patients (seven in each group) were included. No safety or adherence issues were detected in the evaluation of the secondary outcomes.

The RoB evaluation is shown in detail in Supplementary material B. Considerable methodological limitations were found in the studies that evaluated the effectiveness and safety outcomes. These biases can lead to overestimation or underestimation of the effect of the intervention. Although an extensive search of the literature was performed, it was impossible to assess publication bias due to the limited number of studies. The strengths of this review, in addition to the careful literature search, were the rigorous process and the full compliance with a previously registered protocol. Finally, the assessment of the certainty of the body of evidence was performed judiciously using the GRADE approach.

The assessment of the certainty of the evidence for the primary outcomes considering the use of clofazimine in PB leprosy treatment was judged to be very low. A similar judgement was made considering the use of clarithromycin in leprosy patients, with the certainty of evidence classified as very low or low. Given this finding, it is possible to determine that there is little confidence in the effect estimate obtained and that the true effect is probably substantially different from the estimated effect. The results also point to the imprecision of studies available in the literature. Thus, new studies with a good methodological quality and an adequate sample size must be carried out to investigate the effect of the inclusion of clofazimine and clarithromycin in leprosy treatment, as recently recommended by the WHO.

## 5. Conclusion

The addition of clofazimine to PB leprosy treatment helps reduce the negative impact of misclassification with no additional apparent relevant side effects. Although new articles were published after the 2018 WHO recommendations, the effectiveness of this intervention and the inclusion of clarithromycin to substitute for rifampicin in the WHO-MDT still need to be determined. New clinical trials and investment in pharmacovigilance are essential for elucidating these topics.

## Data availability statement

The raw data supporting the conclusions of this article will be made available by the authors, without undue reservation.

## Author contributions

TM, SV, and CG: formal analysis, investigation, resources, supervision, validation, visualization, writing—original draft, and writing—review and editing. EA, JB, and GO: formal analysis, investigation, and resources. All authors contributed to the article and approved the submitted version.

## Supplementary material

Supplementary material A Complete search strategy, the flow charts of study selection, and the tables of excluded studies, including the reasons for exclusion for both reviews.

Supplementary material B Detailed evaluation of all secondary outcomes included in the clofazimine review, clarithromycin review, and certainty of evidence analysis using the GRADE approach.
