# Supplementary material for: Effectiveness and safety of multidrug therapy containing clofazimine for paucibacillary leprosy and clarithromycin for rifampicin-resistant leprosy: a systematic review and meta-analysis
Source: Front Med (Lausanne). 2023 May 10;10:1139304. doi: 10.3389/fmed.2023.1139304 (PMC10206035; doi:10.3389/fmed.2023.1139304)
Supplement: Supplementary material A — Complete search strategy, the flow charts of study selection, and the tables of excluded studies, including the reasons for exclusion for both reviews. [file Data_Sheet_1.docx]

**SUPPLEMENTARY FILE A**

**Complete search strategy, flow diagram of studies selected and table with excluded studies in the selection of reports assessed for eligibility and the reasons for exclusion of clofazimine review and clarithromycin review.**

**Table 1S-A.** Complete search strategy of clofazimine review in general databases and register clinical trials databases

| **MEDLINE via Pubmed: pubmed.ncbi.nlm.nih.gov** |
| --- |
| **Results: 891** |
| ((((((((((("Leprosy"[Mesh]) OR (Hansen's Disease[Text Word])) OR (Hansen Disease[Text Word])) OR (Leprosy[Text Word])) OR ((((((((((((("Leprosy, Paucibacillary"[Mesh]) OR (Leprosy, Paucibacillary[Text Word])) OR (Leprosies, Paucibacillary[Text Word])) OR (Paucibacillary Leprosies[Text Word])) OR (Indeterminate Tuberculoid[Text Word])) OR (Indeterminate Tuberculoids[Text Word])) OR (Tuberculoid, Indeterminate[Text Word])) OR (Tuberculoids, Indeterminate[Text Word])) OR (Paucibacillary Leprosy[Text Word])) OR (Borderline Tuberculoid[Text Word])) OR (Borderline Tuberculoids[Text Word])) OR (Tuberculoid, Borderline[Text Word])) OR (Tuberculoids, Borderline[Text Word]))) OR (("Mycobacterium leprae"[Mesh]) OR (Mycobacterium leprae[Text Word]))) OR ((((((((((((("Leprosy, Tuberculoid"[Mesh]) OR (Leprosy, Tuberculoid[Text Word])) OR (Leprosies, Tuberculoid[Text Word])) OR (Tuberculoid Leprosies[Text Word])) OR (Tuberculoid Leprosy[Text Word])) OR (Leprosy, Neural[Text Word])) OR (Leprosies, Neural[Text Word])) OR (Neural Leprosies[Text Word])) OR (Neural Leprosy[Text Word])) OR (Leprosy, Macular[Text Word])) OR (Leprosies, Macular[Text Word])) OR (Macular Leprosies[Text Word])) OR (Macular Leprosy[Text Word]))) OR ((((((((((((("Leprosy, Lepromatous"[Mesh]) OR (Leprosy, Lepromatous[Text Word])) OR (Lepromatous Leprosies[Text Word])) OR (Lepromatous Leprosy[Text Word])) OR (Leprosies, Lepromatous[Text Word])) OR (Leprosy, Cutaneous[Text Word])) OR (Cutaneous Leprosies[Text Word])) OR (Cutaneous Leprosy[Text Word])) OR (Leprosies, Cutaneous[Text Word])) OR (Leprosy, Nodular[Text Word])) OR (Leprosies, Nodular[Text Word])) OR (Nodular Leprosies[Text Word])) OR (Nodular Leprosy[Text Word]))) OR ((((((((("Leprosy, Borderline"[Mesh]) OR (Leprosy, Borderline[Text Word])) OR (Borderline Leprosies[Text Word])) OR (Borderline Leprosy[Text Word])) OR (Leprosies, Borderline[Text Word])) OR (Leprosy, Dimorphous[Text Word])) OR (Dimorphous Leprosies[Text Word])) OR (Dimorphous Leprosy[Text Word])) OR (Leprosies, Dimorphous[Text Word]))) OR ((((((((("Leprosy, Multibacillary"[Mesh]) OR (Leprosy, Multibacillary[Text Word])) OR (Leprosies, Multibacillary[Text Word])) OR (Multibacillary Leprosies[Text Word])) OR (Midborderline Lepromatous[Text Word])) OR (Lepromatous, Midborderline[Text Word])) OR (Multibacillary Leprosy[Text Word])) OR (Borderline Lepromatous[Text Word])) OR (Lepromatous, Borderline[Text Word])))) OR (Lepros*[Text Word])  AND  (((((((((("Clofazimine"[Mesh]) OR (Clofazimine[Text Word])) OR (N,5-Bis(4-chlorophenyl)-3,5-dihydro-3-((1-methylethyl)imino)-2-phenazinamine[Text Word])) OR (Lamprene[Text Word])) OR (B-663[Text Word])) OR (B 663[Text Word])) OR (B663[Text Word])) OR (G-30,320[Text Word])) OR (G 30,320[Text Word])) OR (G30,320[Text Word])) |
| **EMBASE: embase.com** |
| **Results: 2,655** |
| ('m. leprae infection'/exp OR 'm. leprae infection' OR 'mycobacterium leprae infection'/exp OR 'mycobacterium leprae infection' OR 'elephantiasis graecorum'/exp OR 'elephantiasis graecorum' OR 'hansen disease'/exp OR 'hansen disease' OR 'hanseniasis'/exp OR 'hanseniasis' OR 'infection by mycobacterium leprae'/exp OR 'infection by mycobacterium leprae' OR 'infection of mycobacterium leprae'/exp OR 'infection of mycobacterium leprae' OR 'lepra'/exp OR 'lepra' OR 'leprology'/exp OR 'leprology' OR 'leprosis'/exp OR 'leprosis' OR 'leprosy'/exp OR 'leprosy' OR 'leprous infection'/exp OR 'leprous infection' OR 'morbus hansen'/exp OR 'morbus hansen' OR 'border line leprosy'/exp OR 'border line leprosy' OR 'borderline lepromatous'/exp OR 'borderline lepromatous' OR 'borderline leprosy'/exp OR 'borderline leprosy' OR 'dimorphous leprosy'/exp OR 'dimorphous leprosy' OR 'leprosy, borderline'/exp OR 'leprosy, borderline' OR 'midborderline leprosy'/exp OR 'midborderline leprosy' OR 'lepromatous lepra'/exp OR 'lepromatous lepra' OR 'lepromatous leprosy'/exp OR 'lepromatous leprosy' OR 'leprosy, lepromatous'/exp OR 'leprosy, lepromatous' OR 'leprosy, tuberculoid'/exp OR 'leprosy, tuberculoid' OR 'tuberculoid lepra'/exp OR 'tuberculoid lepra' OR 'tuberculoid leprosy'/exp OR 'tuberculoid leprosy' OR 'mb leprosy'/exp OR 'mb leprosy' OR 'leprosy, multibacillary'/exp OR 'leprosy, multibacillary' OR 'multibacillary leprosy'/exp OR 'multibacillary leprosy' OR 'pb leprosy'/exp OR 'pb leprosy' OR 'leprosy, paucibacillary'/exp OR 'leprosy, paucibacillary' OR 'paucibacillary leprosy'/exp OR 'paucibacillary leprosy' OR 'mycobacterium leprae'/exp OR 'mycobacterium leprae' OR 'bacillus hansen'/exp OR 'bacillus hansen' OR 'bacillus leprae'/exp OR 'bacillus leprae' OR 'hansen bacillus'/exp OR 'hansen bacillus' OR 'icrc bacillus'/exp OR 'icrc bacillus' OR 'leprosy bacillus'/exp OR 'leprosy bacillus' OR 'mycobacterium hansenii'/exp OR 'mycobacterium hansenii' OR 'mycoplasma leprae'/exp OR 'mycoplasma leprae')  AND  ('2 (para chloroanilino) 5 (para chlorophenyl) 3, 5 dihydro 3 (isopropylimino) phenazine'/exp OR '2 (para chloroanilino) 5 (para chlorophenyl) 3, 5 dihydro 3 (isopropylimino) phenazine' OR '3 (4 chloroanilino) 10 (4 chlorophenyl) 2, 10 dihydro 2 isopropyliminophenazine'/exp OR '3 (4 chloroanilino) 10 (4 chlorophenyl) 2, 10 dihydro 2 isopropyliminophenazine' OR '3 (para chloranilino) 10 (para chlorophenyl) 2, 10 dihydro 2 (isopropylimino) phenazine'/exp OR '3 (para chloranilino) 10 (para chlorophenyl) 2, 10 dihydro 2 (isopropylimino) phenazine' OR '3 (para chloroanilino) 10 (para chlorophenyl) 2, 10 dihydro 2 isopropyliminophenazine'/exp OR '3 (para chloroanilino) 10 (para chlorophenyl) 2, 10 dihydro 2 isopropyliminophenazine' OR 'b 663'/exp OR 'b 663' OR 'b663'/exp OR 'b663' OR 'clofazimide'/exp OR 'clofazimide' OR 'clofazimine'/exp OR 'clofazimine' OR 'clofozine'/exp OR 'clofozine' OR 'g 30 320'/exp OR 'g 30 320' OR 'g 30320'/exp OR 'g 30320' OR 'g30 320'/exp OR 'g30 320' OR 'g30320'/exp OR 'g30320' OR 'hansepran'/exp OR 'hansepran' OR 'lam 320'/exp OR 'lam 320' OR 'lam320'/exp OR 'lam320' OR 'lampren'/exp OR 'lampren' OR 'lampren b 663'/exp OR 'lampren b 663' OR 'lamprene'/exp OR 'lamprene' OR 'lamprene capsules'/exp OR 'lamprene capsules' OR 'lapren'/exp OR 'lapren' OR 'lapren sl'/exp OR 'lapren sl' OR 'nsc 141046'/exp OR 'nsc 141046' OR 'qrm 003'/exp OR 'qrm 003' OR 'qrm 007'/exp OR 'qrm 007' OR 'qrm003'/exp OR 'qrm003' OR 'qrm007'/exp OR 'qrm007' OR 'rimino compound b 663'/exp OR 'rimino compound b 663' OR 'rimino phenazine'/exp OR 'rimino phenazine' OR 'riminophenazine'/exp OR 'riminophenazine' OR 'riminophenazine b 663'/exp OR 'riminophenazine b 663')  AND  [embase]/lim |
| **BVS: bvsalud.org** |
| **Results: 706** |
| (((mh:(hanseníase)) OR (doença de hansen) OR (lepra) OR (reação hansênica reversa) OR (reação hansênica tipo 1) OR (reação hansênica tipo 2) OR (reação hansênica do tipo eritema nodoso) OR (reações hansênicas) OR (mh:(leprosy)) OR (hansen disease) OR (hansen's disease) OR (mh:(lepra)) OR (enfermedad de hansen) OR (mal de hansen) OR (exc01.150.252.410.040.552.475.371*) OR (exsp4.012.148.164*)) OR ((mh:(hanseníase multibacilar)) OR (hanseníase dimorfa-dimorfa) OR (hanseníase dimorfa-virchowiana) OR (mh:(leprosy, multibacillary)) OR (borderline lepromatous) OR (lepromatous, borderline) OR (lepromatous, midborderline) OR (leprosies, multibacillary) OR (midborderline lepromatous) OR (multibacillary leprosies) OR (multibacillary leprosy) OR (mh:(lepra multibacilar)) OR (lepromatosa intermedia) OR (lepromatosa limítrofe) OR (exc01.150.252.410.040.552.475.371.775*)) OR ((mh:(hanseníase paucibacilar)) OR (tuberculoide indeterminada) OR (tuberculoide limítrofe) OR (exc01.150.252.410.040.552.475.371.850*) OR (mh:(leprosy paucibacillary)) OR (borderline tuberculoid) OR (borderline tuberculoids) OR (indeterminate tuberculoid) OR (indeterminate tuberculoids) OR (leprosies, paucibacillary) OR (paucibacillary leprosies) OR (paucibacillary leprosy) OR (tuberculoid, borderline) OR (tuberculoid, indeterminate) OR (tuberculoids, borderline) OR (tuberculoids, indeterminate) OR (mh:(lepra paucibacilar)) OR (tuberculoide indeterminada) OR (tuberculoide limítrofe)) OR ((mh:(hanseníase tuberculoide)) OR (hanseníase macular) OR (hanseníase neurítica) OR (exc01.150.252.410.040.552.475.371.850.500*) OR (mh:(leprosy, tuberculoid)) OR (leprosies, macular) OR (leprosies, neural) OR (leprosies, tuberculoid) OR (leprosy, macular) OR (leprosy, neural) OR (macular leprosies) OR (macular leprosy) OR (neural leprosies) OR (neural leprosy) OR (tuberculoid leprosies) OR (tuberculoid leprosy) OR (mh:(lepra tuberculoide)) OR (lepra macular) OR (lepra neural)) OR ((mh:(hanseníase dimorfa)) OR (exc01.150.252.410.040.552.475.371.850.249*) OR (mh:(leprosy borderline)) OR (borderline leprosies) OR (borderline leprosy) OR (dimorphous leprosies) OR (dimorphous leprosy) OR (leprosies, borderline) OR (leprosies, dimorphous) OR (leprosy, dimorphous) OR (mh:(lepra dimorfa)) OR (lepra incierta)) OR ((mh:(hanseníase virchowiana)) OR (hanseníase cutânea) OR (hanseníase nodular) OR (exc01.150.252.410.040.552.475.371.775.500*) OR (mh:(leprosy, lepromatous)) OR (cutaneous leprosies) OR (cutaneous leprosy) OR (lepromatous leprosies) OR (lepromatous leprosy) OR (leprosies, cutaneous) OR (leprosies, lepromatous) OR (leprosies, nodular) OR (leprosy, cutaneous) OR (leprosy, nodular) OR (nodular leprosies) OR (nodular leprosy) OR (mh:(lepra lepromatosa)) OR (lepra cutánea) OR (lepra nodular)) OR ((mh:(mycobacterium leprae)) OR (bacilo de hansen) OR (bacilo de la hanseniasis) OR (bacilo de la lepra) OR (exb03.510.024.962.500.502*) OR (exb03.510.460.400.410.552.552.502*) OR (bacilo da hanseníase) OR (bacilo de hansen)))  AND  ((mh:(clofazimina)) OR (exd03.633.300.704.353*) OR (mh:(clofazamine)) OR (b 663) OR (b-663) OR (b663) OR (g 30,320) OR (g-30,320) OR (g30,320) OR (lamprene) OR (n,5-bis(4-chlorophenyl)-3,5-dihydro-3-((1-methylethyl)imino)-2-phenazinamine) OR (clofacimina)) |
| **LILACS via BVS: lilacs.bvsalud.org** |
| **Results: 69** |
| (((mh☹hanseníase)) OR (doença de lofaz) OR (lepra) OR (reação hansênica reversa) OR (reação hansênica tipo 1) OR (reação hansênica tipo 2) OR (reação hansênica do tipo eritema nodoso) OR (reações hansênicas) OR (mh☹leprosy)) OR (lofaz disease) OR (hansen’s disease) OR (mh☹lepra)) OR (enfermedad de lofaz) OR (mal de lofaz) OR (exc01.150.252.410.040.552.475.371*) OR (exsp4.012.148.164*)) OR ((mh:(hanseníase multibacilar)) OR (hanseníase dimorfa-dimorfa) OR (hanseníase dimorfa-virchowiana) OR (mh:(leprosy, multibacillary)) OR (borderline lepromatous) OR (lepromatous, borderline) OR (lepromatous, midborderline) OR (leprosies, multibacillary) OR (midborderline lepromatous) OR (multibacillary leprosies) OR (multibacillary leprosy) OR (mh:(lepra multibacilar)) OR (lepromatosa intermedia) OR (lepromatosa limítrofe) OR (exc01.150.252.410.040.552.475.371.775*)) OR ((mh:(hanseníase paucibacilar)) OR (tuberculoide indeterminada) OR (tuberculoide limítrofe) OR (exc01.150.252.410.040.552.475.371.850*) OR (mh:(leprosy paucibacillary)) OR (borderline tuberculoid) OR (borderline tuberculoids) OR (indeterminate tuberculoid) OR (indeterminate tuberculoids) OR (leprosies, paucibacillary) OR (paucibacillary leprosies) OR (paucibacillary leprosy) OR (tuberculoid, borderline) OR (tuberculoid, indeterminate) OR (tuberculoids, borderline) OR (tuberculoids, indeterminate) OR (mh:(lepra paucibacilar)) OR (tuberculoide indeterminada) OR (tuberculoide limítrofe)) OR ((mh:(hanseníase tuberculoide)) OR (hanseníase macular) OR (hanseníase neurítica) OR (exc01.150.252.410.040.552.475.371.850.500*) OR (mh:(leprosy, tuberculoid)) OR (leprosies, macular) OR (leprosies, neural) OR (leprosies, tuberculoid) OR (leprosy, macular) OR (leprosy, neural) OR (macular leprosies) OR (macular leprosy) OR (neural leprosies) OR (neural leprosy) OR (tuberculoid leprosies) OR (tuberculoid leprosy) OR (mh:(lepra tuberculoide)) OR (lepra macular) OR (lepra neural)) OR ((mh:(hanseníase dimorfa)) OR (exc01.150.252.410.040.552.475.371.850.249*) OR (mh:(leprosy borderline)) OR (borderline leprosies) OR (borderline leprosy) OR (dimorphous leprosies) OR (dimorphous leprosy) OR (leprosies, borderline) OR (leprosies, dimorphous) OR (leprosy, dimorphous) OR (mh:(lepra dimorfa)) OR (lepra incierta)) OR ((mh:(hanseníase virchowiana)) OR (hanseníase cutânea) OR (hanseníase nodular) OR (exc01.150.252.410.040.552.475.371.775.500*) OR (mh:(leprosy, lepromatous)) OR (cutaneous leprosies) OR (cutaneous leprosy) OR (lepromatous leprosies) OR (lepromatous leprosy) OR (leprosies, cutaneous) OR (leprosies, lepromatous) OR (leprosies, nodular) OR (leprosy, cutaneous) OR (leprosy, nodular) OR (nodular leprosies) OR (nodular leprosy) OR (mh:(lepra lepromatosa)) OR (lepra cutánea) OR (lepra nodular)) OR ((mh:(mycobacterium leprae)) OR (bacilo de hansen) OR (bacilo de la hanseniasis) OR (bacilo de la lepra) OR (exb03.510.024.962.500.502*) OR (exb03.510.460.400.410.552.552.502*) OR (bacilo da hanseníase) OR (bacilo de hansen)))  AND  ((mh:(clofazimina)) OR (exd03.633.300.704.353*) OR (mh:(clofazamine)) OR (b 663) OR (b-663) OR (b663) OR (g 30,320) OR (g-30,320) OR (g30,320) OR (lamprene) OR (n,5-bis(4-chlorophenyl)-3,5-dihydro-3-((1-methylethyl)imino)-2-phenazinamine) OR (clofacimina))  AND  ( db:("LILACS")) |
| **Cochrane Library: cochranelibrary.com** |
| **Results: 72** |
| #1 MeSH descriptor: [Leprosy] explode all trees 298  #2 Hansen Disease 1700  #3 Hansen's Disease 1700  #4 Leprosy 866  #5 MeSH descriptor: [Leprosy, Borderline] explode all trees 20  #6 Leprosy, Dimorphous 0  #7 Leprosies, Borderline 0  #8 Borderline Leprosy 56  #9 Dimorphous Leprosy 0  #10 Borderline Leprosies 0  #11 Dimorphous Leprosies 0  #12 Leprosies, Dimorphous 0  #13 MeSH descriptor: [Leprosy, Multibacillary] explode all trees 53  #14 Multibacillary Leprosies 0  #15 Lepromatous, Borderline 43  #16 Midborderline Lepromatous 2  #17 Borderline Lepromatous 43  #18 Leprosies, Multibacillary 0  #19 Multibacillary Leprosy 123  #20 Lepromatous, Midborderline 2  #21 MeSH descriptor: [Leprosy, Paucibacillary] explode all trees 31  #22 Paucibacillary Leprosies 0  #23 Indeterminate Tuberculoid 4  #24 Indeterminate Tuberculoids 0  #25 Leprosies, Paucibacillary 0  #26 Tuberculoids, Indeterminate 0  #27 Tuberculoid, Indeterminate 4  #28 Borderline Tuberculoid 19  #29 Borderline Tuberculoids 0  #30 Tuberculoid, Borderline 19  #31 Tuberculoids, Borderline 0  #32 Paucibacillary Leprosy 66  #33 MeSH descriptor: [Leprosy, Tuberculoid] explode all trees 10  #34 Leprosy, Macular 5  #35 Leprosies, Macular 0  #36 Macular Leprosies 0  #37 Macular Leprosy 5  #38 Neural Leprosy 23  #39 Leprosies, Neural 0  #40 Leprosy, Neural 23  #41 Neural Leprosies 0  #42 Leprosies, Tuberculoid 0  #43 Tuberculoid Leprosy 34  #44 Tuberculoid Leprosies 0  #45 MeSH descriptor: [Leprosy, Lepromatous] explode all trees 47  #46 Lepromatous Leprosies 0  #47 Lepromatous Leprosy 142  #48 Leprosies, Lepromatous 0  #49 Leprosies, Cutaneous 0  #50 Nodular Leprosy 5  #51 Leprosy, Cutaneous 55  #52 Nodular Leprosies 0  #53 Cutaneous Leprosy 55  #54 Cutaneous Leprosies 0  #55 Leprosies, Nodular 0  #56 Leprosy, Nodular 5  #57 MeSH descriptor: [Mycobacterium leprae] explode all trees 67  #58 {OR #1-#57} 2545  #59 MeSH descriptor: [Clofazimine] explode all trees 100  #60 Lamprene 7  #61 G30,320 0  #62 G-30,320 3  #63 G 30,320 3  #64 B663 6  #65 B 663 1080  #66 B-663 7  #67 {OR #59-#66} 1189  #68 #58 AND #67 72 |
| **Clinicaltrials.gov** |
| **Results: 2** |
| Leprosy AND Clofazimine |
| **International Clinical Trials Registry Platform: who.int/clinical-trials-registry-platform** |
| **Results: 9** |
| Leprosy AND Clofazimine |
| **OpenGrey via Data Archiving and Network Services (DANS): easy.dans.knaw.nl/ui/datasets/id/easy-dataset:200362** |
| **Results: 0** |
| Leprosy AND Clofazimine |
| **Web of Science: https://www-webofscience.ez54.periodicos.capes.gov.br/wos/woscc/basic-search** |
| **Results: 414** |
| (((((((((((ALL=(leprosy)) OR ALL=(Hansen's Disease)) OR ALL=(Paucibacillary Leprosies)) OR ALL=(Mycobacterium leprae)) OR ALL=(Tuberculoid Leprosy)) OR ALL=(Macular Leprosies)) OR ALL=(Lepromatous Leprosy)) OR ALL=(Cutaneous Leprosy)) OR ALL=(Borderline Leprosy)) OR ALL=(Dimorphous Leprosies)) OR ALL=(Multibacillary Leprosies)) AND ALL=(clofazimine) |
| **Scopus: scopus.com** |
| **Results: 4,023** |
| ( ALL ( lepros* ) OR ALL ( "hansen* disease" ) OR ALL ( "mycobacterium leprae" ) OR ALL ( "M. leprae" ) AND ALL ( clofazimine ) ) |

**Thesis databases searched (clofazimine review)**

1) Queen’s Univerity Library Catalogue

<https://ocul-qu.primo.exlibrisgroup.com/discovery/search?query=any,contains,leprosy&tab=Everything&search_scope=MyInst_and_CI&vid=01OCUL_QU:QU_DEFAULT&facet=rtype,include,dissertations&offset=0>

<https://ocul-qu.primo.exlibrisgroup.com/discovery/search?query=any,contains,Clofazimine%20and%20leprosy&tab=Everything&search_scope=MyInst_and_CI&vid=01OCUL_QU:QU_DEFAULT&offset=100>

2) Queen’s Univerity Research & Learning Repository (QSpace): Theses & Dissertations

<https://qspace.library.queensu.ca/discover?scope=%2F&query=leprosy+&submit=>

<https://qspace.library.queensu.ca/discover?scope=1974%2F196&query=leprosy+and+Clofazimine&submit=>

3) Theses Canada Portal

<https://www.bac-lac.gc.ca/eng/services/theses/Pages/list.aspx?AW_S=leprosy>

<https://www.bac-lac.gc.ca/eng/services/theses/Pages/list.aspx?TW_S=leprosy+and+Clofazimine>

4) The Directory of Open Access Repositories

<http://v2.sherpa.ac.uk/cgi/search/repository/basic?repository_name-auto=leprosy&_action_search=Search&screen=Search&repository_name-auto_merge=ALL>

<https://v2.sherpa.ac.uk/cgi/search/repository/basic?repository_name-auto=leprosy+and+clofazimine&_action_search=Search&screen=Search&repository_name-auto_merge=ALL>

5) ProQuest Open Access Dissertations and Theses

<https://www.proquest.com/resultsol/61E9B086CA3D4659PQ/1>

<https://www.proquest.com/resultsol/8A118434DAB14BCDPQ/1>

6) OpenThesis.org

7) PhdData.org

8) Center for Research Libraries

<http://catalog.crl.edu/search/Y?SEARCH=leprosy&searchscope=4&x=6&y=15>

<http://catalog.crl.edu/search~S4/?searchtype=Y&searcharg=leprosy+and+clofazimine&searchscope=4&sortdropdown=-&SORT=DZ&extended=0&SUBMIT=Procurar&searchlimits=&searchorigarg=Yleprosy+and+clofazimine>

9) Networked Digital Library of Theses and Dissertations

<http://search.ndltd.org/search.php?q=leprosy&source_set_names=&year_start=&year_end=>

<http://search.ndltd.org/search.php?q=leprosy+and+clofazimine&source_set_names=&year_start=&year_end=>

10) National Library of Australia’s Trove Service

<https://trove.nla.gov.au/book/result?l-format=Thesis&q=leprosy>

<https://trove.nla.gov.au/search/category/books?keyword=leprosy%20and%20clofazimine>

11) United Kingdom E-Theses Online Service

<https://ethos.bl.uk/SearchResults.do>

12) DART-Europe

<https://www.dart-europe.org/basic-results.php?kw%5B%5D=leprosy&f=n>

<https://www.dart-europe.org/basic-results.php?kw%5B%5D=leprosy+and+clofazimine&f=n>

13) Système universitaire de documentation

[http://www.sudoc.abes.fr/cbs/xslt//DB=2.1/SET=1/TTL=1/CMD?ACT=SRCHA&IKT=1016&SRT=RLV&TRM=leprosy](http://www.sudoc.abes.fr/cbs/xslt/DB=2.1/SET=1/TTL=1/CMD?ACT=SRCHA&IKT=1016&SRT=RLV&TRM=leprosy)

<http://www.sudoc.abes.fr/cbs/xslt//DB=2.1/SET=2/TTL=1/CMD?ACT=SRCHA&IKT=1016&SRT=RLV&TRM=leprosy+and+clofazimine>

<http://www.sudoc.abes.fr/cbs/xslt/DB=2.1//SRCH?IKT=12&TRM=22481706X>

14) Fichier central des thèses

<http://theses.fr/fr/?q=leprosy+>

<http://theses.fr/fr/?q=leprosy+and+clofazimine>

15) Deutschen National Bibliothek

<https://portal.dnb.de/opac/simpleSearch?query=leprosy>+

<https://portal.dnb.de/opac/simpleSearch?query=leprosy+and+Clofazimine>

16) Bibliotecario de la Universidad Nacional Autónoma de México

<https://eds.s.ebscohost.com/eds/resultsadvanced?vid=2&sid=0984d99a-3cc3-4789-88a9-cd1f498e6158%40redis&bquery=leprosy&bdata=JmNsaTA9RlQmY2x2MD1ZJmxhbmc9ZXMmdHlwZT0xJnNlYXJjaE1vZGU9U3RhbmRhcmQmc2l0ZT1lZHMtbGl2ZQ%3d%3d>

<https://eds.s.ebscohost.com/eds/resultsadvanced?vid=3&sid=0984d99a-3cc3-4789-88a9-cd1f498e6158%40redis&bquery=leprosy+AND+clofazimine&bdata=JmNsaTA9RlQmY2x2MD1ZJmxhbmc9ZXMmdHlwZT0xJnNlYXJjaE1vZGU9U3RhbmRhcmQmc2l0ZT1lZHMtbGl2ZQ%3d%3d>

17) National Academic Research and Collaborations Information System

<https://www.narcis.nl/search/uquery/leprosy/Language/EN>

<https://www.narcis.nl/search/uquery/leprosy%20and%20clofazimine/Language/EN>

18) Spain’s Ministry of Education thesis database

<https://administracion.gob.es/buscador/buscadorSimple.htm?q=leprosy&site=PAG_ES&filter=p&busqueda=true&buscar=Buscar&buscar=#gsc.tab=0&gsc.q=leprosy&gsc.page=1>

<https://administracion.gob.es/buscador/buscadorSimple.htm?q=leprosy+and+clofazimine&site=PAG_ES&filter=p&busqueda=true&buscar=Buscar&buscar=#gsc.tab=0&gsc.q=leprosy%20and%20clofazimine&gsc.page=1>

19) Biblioteca Virtual Miguel de Cervantes

<http://www.cervantesvirtual.com/buscador/?q=leprosy>

<https://www.cervantesvirtual.com/buscador/?q=leprosy+and+clofazimine>

20) Universidad Complutense de Madrid’s catalogue

<https://ucm.on.worldcat.org/search?databaseList=&queryString=leprosy>

<https://ucm.on.worldcat.org/search?queryString=leprosy%20and%20clofazimine&databaseList=&clusterResults=false&groupVariantRecords=true&database=Z-dissert&changedFacet=database>

21) Tesis Doctorals en Xarxa

[[https://tdx.cat/discover?scope=%2F&query=leprosy&submit=](https://tdx.cat/discover?scope=%2F&query=leprosy+and+clofazimine&submit=&ocult=0&scope=%2F&rpp=10)](https://tdx.cat/discover?scope=%2F&query=leprosy&submit=)

<https://tdx.cat/discover?scope=%2F&query=leprosy+and+clofazimine&submit=&ocult=0&scope=%2F&rpp=10>

22) Swiss National Library

<https://www.nb.admin.ch/snl/de/home/suche.html#leprosy>

<https://www.nb.admin.ch/snl/de/home/suche.html#leprosy%20and%20clofazimine>

23) Universidade de São Paulo

<https://teses.usp.br/index.php?option=com_jumi&fileid=12&Itemid=77&lang=pt-br&filtro=leprosy>

<https://teses.usp.br/index.php?option=com_jumi&fileid=12&Itemid=77&lang=pt-br&filtro=leprosy%20and%20clofazimine>

24) Universidade Federal de São Paulo

<https://repositorio.unifesp.br/discover?query=leprosy&filtertype=type&filter_relational_operator=equals&filter=Tese+de+doutorado>

<https://repositorio.unifesp.br/discover?scope=%2F&query=leprosy+and+clofazimine&submit=&filtertype_0=type&filter_relational_operator_0=equals&filter_0=Tese+de+doutorado>

25) Universidade Estadual de Campinas

<http://www.bibliotecadigital.unicamp.br/document/list_index.php?palavra=leprosy&type_list=3&tid=498&stid%5B%5D=0&sent=s>

<http://www.bibliotecadigital.unicamp.br/cgi-bin/search.cgi?q=leprosy+and+clofazimine&fl=m&ps=25&uid=0&lg=pt_BR&wf=0>

26) Universidade Estadual Paulista

<https://repositorio.unesp.br/handle/11449/77291/discover?query=leprosy+&submit=>

<https://repositorio.unesp.br/handle/11449/77291/discover?query=leprosy+and+clofazimine&submit=>

27) Pontifícia Universidade Católica de Campinas

<https://itms.libsteps.com/PUCC/index.php/result?charset=utf-8&category1=0&category2=0&category3=0&text1=leprosy&text2=&text3=&op=0&op2=0&year1=&year2=&dbnum=78661+64001+92851+54551+34771+39871+1401+3371+63601+78881+63671+65231+78651+75281&display=30&recstart=1&sort=rel&id=164808612944#/?recstart=1&display=20&dbnum=78651&key=&type=search&reload=off>

<https://itms.libsteps.com/PUCC/index.php/result?id=164808622237368&category1=0&category2=0&category3=0&text1=leprosy%20and%20clofazimine&text2=&text3=&op=0&op2=0&year1=&year2=&dbnum=78661%2064001%2092851%2054551%2034771%2039871%201401%203371%2063601%2078881%2063671%2065231%2078651%2075281&display=30&recstart=1&sort=rel#/?recstart=1&display=20&dbnum=78651&key=&type=search&reload=off>

28) Universidade Católica de Santos

<http://biblioteca.unisantos.br/pergamum/biblioteca/>

29) Pontifícia Universidade Católica de São Paulo

<https://sapientia.pucsp.br/simple-search?query=leprosy>

<https://sapientia.pucsp.br/simple-search?query=leprosy+and+clofazimine>

30) Universidade de Guarulhos

<http://tede.ung.br/simple-search?query=leprosy>

<http://tede.ung.br/simple-search?query=leprosy+clofazimine>

31) Universidade de Taubaté

<https://unitau.br/busca/?termo=leprosy>

<https://unitau.br/busca/?termo=leprosy+and+clofazimine>

32) Universidade do Oeste Paulista

<https://www.unoeste.br/Biblioteca>

33) Universidade Presbiteriana Mackenzie

<http://tede.mackenzie.br/jspui/simple-search?query=leprosy>

<http://tede.mackenzie.br/jspui/simple-search?location=%2F&query=leprosy+and+clofazimine&rpp=10&sort_by=score&order=desc>

34) Universidade Metodista de São Paulo

<http://portal.metodista.br/posreligiao/publicacoes/teses-e-dissertacoes/teses-de-doutorado-1993-a-2003-1>

<http://portal.metodista.br/posreligiao/publicacoes/teses-e-dissertacoes/teses-de-doutorado-2004-a-2009>

<http://portal.metodista.br/posreligiao/publicacoes/teses-e-dissertacoes/teses-de-doutorado-2010-a-2019>

<http://portal.metodista.br/posreligiao/publicacoes/teses-e-dissertacoes/dissertacoes-de-mestrado-1981-a-2000-1>

<http://portal.metodista.br/posreligiao/publicacoes/teses-e-dissertacoes/dissertacoes-de-mestrado-2001-a-2009-1>

<http://portal.metodista.br/posreligiao/publicacoes/teses-e-dissertacoes/dissertacoes-de-mestrado-2010-a-2019>

35) Universidade Anhembi Morumbi

36) Universidade Nove de Julho

<https://bibliotecatede.uninove.br/simple-search?query=leprosy>

<https://bibliotecatede.uninove.br/simple-search?location=%2F&query=leprosy+and+clofazimine&rpp=10&sort_by=score&order=desc>

37) Universidade Federal de São Carlos

<https://repositorio.ufscar.br/handle/ufscar/1538/discover?query=leprosy+&submit=>

<https://repositorio.ufscar.br/handle/ufscar/1538/discover?query=leprosy+and+clofazimine&submit=>

38) Universidade Federal do Acre

<http://www2.ufac.br/ppge/@@busca?SearchableText=leprosy>

<http://www2.ufac.br/ppge/@@busca?SearchableText=leprosy+and+clofazimine>

39) Universidade Federal de Alagoas

<http://www.repositorio.ufal.br/simple-search?query=leprosy>

<http://www.repositorio.ufal.br/simple-search?location=%2F&query=leprosy+and+clofazimine&rpp=10&sort_by=score&order=desc>

40) Universidade Federal do Amazonas

<https://tede.ufam.edu.br/simple-search?query=leprosy>

<https://tede.ufam.edu.br/simple-search?location=%2F&query=leprosy+and+clofazimine&rpp=10&sort_by=score&order=desc>

41) Universidade Salvador

<https://eds.s.ebscohost.com/eds/results?vid=0&sid=30c85ec3-7c3d-4198-8c9f-4f4cb85d6e39%40redis&bquery=leprosy&bdata=JmNsaTA9RlQxJmNsdjA9WSZsYW5nPXB0LWJyJnR5cGU9MCZzZWFyY2hNb2RlPUFuZCZzaXRlPWVkcy1saXZl>

<https://eds.s.ebscohost.com/eds/results?vid=3&sid=30c85ec3-7c3d-4198-8c9f-4f4cb85d6e39%40redis&bquery=leprosy+and+clofazimine&bdata=JmNsaTA9RlQxJmNsdjA9WSZsYW5nPXB0LWJyJnR5cGU9MCZzZWFyY2hNb2RlPUFuZCZzaXRlPWVkcy1saXZl>

42) Universidade Estadual de Feira de Santana

<http://tede2.uefs.br:8080/simple-search?query=leprosy>

<http://tede2.uefs.br:8080/simple-search?location=%2F&query=leprosy+and+clofazimine&rpp=10&sort_by=score&order=desc>

43) Universidade de Federal da Bahia

<https://repositorio.ufba.br/ri/simple-search?query=leprosy&submit=Ir>

<https://repositorio.ufba.br/simple-search?location=%2F&query=leprosy+and+clofazimine&rpp=10&sort_by=score&order=desc>

44) Universidade do Estado da Bahia

<http://www.cdi.uneb.br/site/?cat-trabalhos-academicos=pgdr&termo=filtro-todos&termo-valor=leprosy>

<http://www.cdi.uneb.br/site/?cat-trabalhos-academicos=pgdr&termo=filtro-todos&termo-valor=leprosy+and+clofazimine>

45) Universidade Federal do Ceará

<http://www.repositorio.ufc.br/simple-search?query=leprosy>

<https://repositorio.ufc.br/simple-search?location=%2F&query=leprosy+and+clofazimine&rpp=10&sort_by=score&order=desc>

(<http://search.ndltd.org/show.php?id=oai%3Aunion.ndltd.org%3AIBICT%2Foai%3Awww.repositorio.ufc.br%3Ariufc%2F2724&back=http%3A%2F%2Fsearch.ndltd.org%2Fsearch.php%3Fq%3Dleprosy%2Band%2Bclofazimine%26source_set_names%3D%26year_start%3D%26year_end%3D>)

(<http://search.ndltd.org/show.php?id=oai%3Aunion.ndltd.org%3AIBICT%2Foai%3Awww.repositorio.ufc.br%3Ariufc%2F13853&back=http%3A%2F%2Fsearch.ndltd.org%2Fsearch.php%3Fq%3Dleprosy%2Band%2Bclofazimine%26source_set_names%3D%26year_start%3D%26year_end%3D> )

46) Universidade de Brasília

<https://repositorio.unb.br/simple-search?query=leprosy>

<https://repositorio.unb.br/simple-search?location=%2F&query=leprosy+and+clofazimine&rpp=10&sort_by=score&order=desc>

47) Universidade Católica de Brasília

<https://bdtd.ucb.br:8443/jspui/simple-search?query=leprosy>

<https://bdtd.ucb.br:8443/jspui/simple-search?location=%2F&query=leprosy+and+clofazimine&rpp=10&sort_by=score&order=desc>

48) Universidade Federal do Espírito Santo

<http://repositorio.ufes.br/simple-search?query=leprosy>

<http://repositorio.ufes.br/simple-search?location=%2F&query=leprosy+and+clofazimine&rpp=10&sort_by=score&order=desc>

49) Universidade Federal de Goiás

<https://repositorio.bc.ufg.br/tede/simple-search?query=leprosy>

<https://repositorio.bc.ufg.br/tede/simple-search?location=%2F&query=leprosy+and+clofazimine&rpp=10&sort_by=score&order=desc>

50) Universidade Católica de Goiás

<http://tede2.pucgoias.edu.br:8080/simple-search?query=leprosy>

<http://tede2.pucgoias.edu.br:8080/simple-search?location=%2F&query=leprosy+and+clofazimine&rpp=10&sort_by=score&order=desc>

51) Universidade Federal Mato Grosso do Sul

<https://repositorio.ufms.br:8443/jspui/handle/123456789/52/browse?type=subject&order=ASC&rpp=20&starts_with=leprosy>

<https://repositorio.ufms.br/handle/123456789/52/browse?type=subject&sort_by=1&order=ASC&rpp=20&etal=-1&value=Leprosy&starts_with=leprosy+and+clofazimine>

52) Universidade Federal da Grande Dourados

<http://repositorio.ufgd.edu.br/jspui/simple-search?query=leprosy>

<https://repositorio.ufgd.edu.br/jspui/simple-search?location=%2F&query=leprosy+and+clofazimine&rpp=10&sort_by=score&order=desc>

53) Universidade Católica Dom Bosco

<https://site.ucdb.br/cursos/4/mestrado-e-doutorado/32/mestrado-e-doutorado-em-educacao/13167/doutorado-em-educacao/13192/teses-defendidas/13197/#busca=leprosy>

<https://site.ucdb.br/cursos/4/mestrado-e-doutorado/32/mestrado-e-doutorado-em-educacao/13167/doutorado-em-educacao/13192/teses-defendidas/13197/#busca=leprosy%20and%20clofazimine>

54) Universidade Federal de Uberlândia

<http://repositorio.ufu.br/simple-search?query=leprosy>

<http://repositorio.ufu.br/simple-search?location=%2F&query=leprosy+and+clofazimine&rpp=10&sort_by=score&order=desc>

55) Universidade Federal de Juiz de Fora

<http://repositorio.ufjf.br:8080/jspui/simple-search?query=leprosy>

<http://repositorio.ufjf.br:8080/jspui/simple-search?location=%2F&query=leprosy+and+clofazimine&rpp=10&sort_by=score&order=desc>

56) Universidade Federal de Lavras

<http://repositorio.ufla.br/simple-search?query=leprosy+>

<http://repositorio.ufla.br/simple-search?location=%2F&query=leprosy+and+clofazimine&rpp=10&sort_by=score&order=desc>

57) Universidade Federal de Minas Gerais

<https://repositorio.ufmg.br/simple-search?query=leprosy>

<https://repositorio.ufmg.br/simple-search?location=%2F&query=leprosy+and+clofazimine&rpp=10&sort_by=score&order=desc>

58) Universidade Federal de Ouro Preto

<https://www.repositorio.ufop.br/simple-search?location=%2F&query=leprosy+&rpp=10&sort_by=score&order=desc>

<https://www.repositorio.ufop.br/simple-search?location=%2F&query=leprosy+and+clofazimine&rpp=10&sort_by=score&order=desc>

59) Universidade Federal de Viçosa

<https://www.locus.ufv.br/handle/123456789/1/discover?query=leprosy+&submit=Ir>

<https://www.locus.ufv.br/simple-search?location=%2F&query=leprosy+and+clofazimine&rpp=10&sort_by=score&order=desc>

60) Pontifícia Universidade Católica de Minas Gerais

<https://web.sistemas.pucminas.br/BDP/PUC%20Minas>

61) Centro Universitário de Caratinga

<https://saas3.bnweb.org/unec/bnportal/pt-BR/search?exp=leprosy%20>

<https://saas3.bnweb.org/unec/bnportal/pt-BR/search?exp=leprosy%20and%20clofazimine>

62) Universidade Federal do Pará

<http://www.repositorio.ufpa.br/jspui/handle/2011/2289/browse?type=subject&order=ASC&rpp=20&starts_with=leprosy>

<http://www.repositorio.ufpa.br/jspui/handle/2011/2289/browse?type=subject&order=ASC&rpp=20&starts_with=leprosy+and+clofazimine>

63) Universidade Federal da Paraíba

<https://repositorio.ufpb.br/jspui/simple-search?query=leprosy>

<https://repositorio.ufpb.br/jspui/simple-search?location=%2F&query=leprosy+and+clofazimine&rpp=10&sort_by=score&order=desc>

64) Universidade Federal do Paraná

<https://acervodigital.ufpr.br/handle/1884/284/discover?query=leprosy+&submit=>

<https://acervodigital.ufpr.br/handle/1884/284/discover?query=leprosy+and+clofazimine&submit=>

65) Pontifícia Universidade Católica do Paraná

66) Universidade Estadual de Londrina

<http://www.bibliotecadigital.uel.br/document/results.php?words=leprosy>

<http://www.bibliotecadigital.uel.br/document/results.php?method=and&sort=score&matchesperpage=10&words=leprosy+and+clofazimine>

67) Universidade Estadual do Oeste do Paraná

<http://tede.unioeste.br/simple-search?query=leprosy>

<https://tede.unioeste.br/simple-search?location=%2F&query=leprosy+and+clofazimine&rpp=10&sort_by=score&order=desc>

68) Universidade Estadual de Ponta Grossa

<https://tede2.uepg.br/jspui/simple-search?location=%2F&query=leprosy+&rpp=10&sort_by=score&order=desc>

<https://tede2.uepg.br/jspui/simple-search?location=%2F&query=leprosy+and+clofazimine&rpp=10&sort_by=score&order=desc>

69) Universidade Estadual de Maringá

<http://nou-rau.uem.br/nou-rau/document/results.php?words=leprosy>

<http://nou-rau.uem.br/nou-rau/document/results.php?method=and&sort=score&matchesperpage=10&words=leprosy+and+clofazimine>

70) Universidade Federal de Pernambuco

<https://repositorio.ufpe.br/handle/123456789/50/browse?type=subject&order=ASC&rpp=20&value=Leprosy>

<https://repositorio.ufpe.br/simple-search?query=leprosy+and+clofazimine>

71) Universidade de Pernambuco

<https://w2.solucaoatrio.net.br/somos/upe-csaude/index.php/pt/doutorado/teses-doutorado>

72) Universidade Católica de Pernambuco

<http://tede2.unicap.br:8080/simple-search?query=leprosy>

<http://tede2.unicap.br:8080/simple-search?location=%2F&query=leprosy+and+clofazimine&rpp=10&sort_by=score&order=desc>

73) Universidade Federal do Piauí

<https://sigaa.ufpi.br/sigaa/public/biblioteca/buscaPublicaAcervo.jsf>

74) Universidade do Estado do Rio de Janeiro

<https://www.bdtd.uerj.br:8443/simple-search?location=%2F&query=leprosy+&rpp=10&sort_by=score&order=desc>

<https://www.bdtd.uerj.br:8443/simple-search?location=%2F&query=leprosy+and+clofazimine&rpp=10&sort_by=score&order=desc>

75) Universidade Federal do Estado do Rio de Janeiro

<https://pantheon.ufrj.br/simple-search?query=leprosy>+

<https://pantheon.ufrj.br/simple-search?location=%2F&query=leprosy+and+clofazimine&rpp=10&sort_by=score&order=desc>

76) Universidade Federal Fluminense

<https://app.uff.br/riuff/discover?scope=%2F&query=leprosy+&submit=>

<https://app.uff.br/riuff/discover?scope=%2F&query=leprosy+and+clofazimine&submit=>

77) Universidade Federal do Rio de Janeiro

<https://pantheon.ufrj.br/simple-search?location=%2F&query=leprosy&rpp=10&sort_by=score&order=desc>

<https://pantheon.ufrj.br/simple-search?query=leprosy+and+clofazimine>

78) Universidade Federal Rural do Rio de Janeiro

<https://tede.ufrrj.br/jspui/simple-search?query=leprosy>

<https://tede.ufrrj.br/jspui/simple-search?location=%2F&query=leprosy+and+clofazimine&rpp=10&sort_by=score&order=desc>

79) Pontifícia Universidade Católica do Rio de Janeiro

<https://www.maxwell.vrac.puc-rio.br/colecao.php?strSearch=leprosy&strTit=&strAut=>

<https://www.maxwell.vrac.puc-rio.br/colecao.php?strSearch=leprosy+and+clofazimine&strTit=&strAut=>

80) Fundação Oswaldo Cruz

<http://teses.icict.fiocruz.br/cgi-bin/wxis1660.exe/lildbi/iah/>

<https://www.arca.fiocruz.br/simple-search?query=leprosy+and+clofazimine>

81) Universidade Federal do Rio Grande do Norte

<https://sigaa.ufrn.br/sigaa/public/biblioteca/buscaPublicaAcervo.jsf>

82) Universidade Potiguar

<https://eds.p.ebscohost.com/eds/results?vid=3&sid=6586bbe7-fdf6-49c3-96b8-b260c6f5d992%40redis&bquery=leprosy&bdata=JmNsaTA9RlQxJmNsdjA9WSZsYW5nPXB0LWJyJnR5cGU9MCZzZWFyY2hNb2RlPUFuZCZzaXRlPWVkcy1saXZl>

<https://eds.p.ebscohost.com/eds/results?vid=6&sid=6586bbe7-fdf6-49c3-96b8-b260c6f5d992%40redis&bquery=leprosy+and+clofazimine&bdata=JmNsaTA9RlQxJmNsdjA9WSZsYW5nPXB0LWJyJnR5cGU9MCZzZWFyY2hNb2RlPUFuZCZzaXRlPWVkcy1saXZl>

83) Universidade Federal do Rio Grande do Sul

<https://sabi.ufrgs.br/F/XHY991GN9UH98297D9UI6VX1YUXJ746348FNIN2DMY1XU18HKE-17446?func=find-b&request=leprosy&find_code=WRD&adjacent=N&x=49&y=5&filter_code_2=WLN&filter_request_2=&filter_code_3=WYR&filter_request_3=&filter_code_4=WYR&filter_request_4=>

<https://sabi.ufrgs.br/F/LELR5FVGUF3HAXPSYYNPQRJHV23KCB7FXTA7G94TIRM7TPSALB-11050?func=find-b&REQUEST=leprosy+and+clofazimine&find_code=WRD&ADJACENT=N&x=44&y=8>

84) Universidade Federal de Santa Maria

<https://repositorio.ufsm.br/discover?scope=%2F&query=leprosy+&submit=>

<https://repositorio.ufsm.br/discover?scope=%2F&query=leprosy+and+clofazimine&submit=>

85) Fundação Universidade Federal do Rio Grande

https://lume.ufrgs.br/handle/10183/1/discover?query=leprosy+and+clofazimine&submit=&dateMode=select&querytype_0=title&query_relational_operator_0=contains&query_value_0=&querytype_1=authortd&query_relational_operator_1=contains&query_value_1=&querytype_2=orientador&query_relational_operator_2=contains&query_value_2=&querytype_3=subject&query_relational_operator_3=contains&query_value_3=&querytype_4=dateIssued&query_relational_operator_4=contains&query_value_4=&select_mes_inicio_4=&select_dia_inicio_4=&select_mes_fim_4=&select_dia_fim_4=&querytype_5=dataAno&query_relational_operator_5=equals&query_value_5=&querytype_6=nivelAcademico&query_relational_operator_6=equals&query_value_6=&querytype_7=tipo&query_relational_operator_7=equals&query_value_7=&querytype_8=idioma&query_relational_operator_8=equals&query_value_8=&querytype_9=formatoArquivo&query_relational_operator_9=equals&query_value_9=&querytype_10=serie&query_relational_operator_10=contains&query_value_10=&querytype_11=author&query_relational_operator_11=contains&query_value_11=&querytype_12=acervo&query_relational_operator_12=contains&query_value_12=&querytype_13=descriptionSection&query_relational_operator_13=contains&query_value_13=&querytype_14=tipoAto&query_relational_operator_14=contains&query_value_14=&querytype_15=natureza&query_relational_operator_15=contains&query_value_15=&querytype_16=numeroAto&query_relational_operator_16=contains&query_value_16=&querytype_17=orgao&query_relational_operator_17=contains&query_value_17=&querytype_18=dataFinal&query_relational_operator_18=contains&query_value_18=&select_mes_inicio_18=&select_dia_inicio_18=&select_mes_fim_18=&select_dia_fim_18=&querytype_19=programa&query_relational_operator_19=contains&query_value_19=&querytype_20=entrevistado&query_relational_operator_20=contains&query_value_20=&querytype_21=grandeArea&query_relational_operator_21=contains&query_value_21=&querytype_22=tipoDeApresentacao&query_relational_operator_22=contains&query_value_22=&querytype_23=areaTematica&query_relational_operator_23=contains&query_value_23=&querytype_24=coordenador&query_relational_operator_24=contains&query_value_24=&querytype_25=origem&query_relational_operator_25=contains&query_value_25=&querytype_26=unidade&query_relational_operator_26=contains&query_value_26=&querytype_27=status&query_relational_operator_27=contains&query_value_27=&querytype_28=curso&query_relational_operator_28=contains&query_value_28=&querytype_29=nivelDeEnsino&query_relational_operator_29=contains&query_value_29=&querytype_30=tipoDeMaterial&query_relational_operator_30=contains&query_value_30=

86) Universidade Católica de Pelotas

<https://pos.ucpel.edu.br/ppgsc/?s=leprosy>

<https://pos.ucpel.edu.br/ppgsc/?s=leprosy+and+clofazimine>

87) Pontifícia Universidade Católica do Rio Grande do Sul

<http://tede2.pucrs.br/tede2/simple-search?query=leprosy>

<http://tede2.pucrs.br/tede2/simple-search?location=%2F&query=leprosy+and+clofazimine&rpp=10&sort_by=score&order=desc>

88) Universidade de Passo Fundo

<https://secure.upf.br/pergamum/biblioteca/index.php>

89) Universidade de Caxias do Sul

<https://repositorio.ucs.br/xmlui/handle/11338/37/discover?query=leprosy+&submit=>

<https://repositorio.ucs.br/xmlui/handle/11338/37/discover?query=leprosy+and+clarithromycin&submit=>

90) Universidade do Vale do Rio dos Sinos

<http://www.repositorio.jesuita.org.br/handle/UNISINOS/1565/discover?query=leprosy+&submit=>+++

<http://www.repositorio.jesuita.org.br/handle/UNISINOS/1565/discover?query=leprosy+and+clofazimine&submit=>+++

91) Universidade Luterana do Brasil

<http://www.ppgecim.ulbra.br/teses/index.php/ppgecim/search/search>

92) Universidade Federal de Rondônia

<https://ri.unir.br/jspui/simple-search?location=%2F&query=leprosy+&rpp=10&sort_by=score&order=desc&filter_field_1=subject&filter_type_1=equals&filter_value_1=Hansen%C3%ADase>

<https://ri.unir.br/jspui/simple-search?location=%2F&query=leprosy+and+Clofazimine&rpp=10&sort_by=score&order=desc&filter_field_1=subject&filter_type_1=equals&filter_value_1=Hansen%C3%ADase>

93) Universidade Federal de Santa Catarina

<https://repositorio.ufsc.br/handle/123456789/74645/discover?filtertype_1=title&filter_relational_operator_1=contains&filter_1=leprosy&submit_apply_filter=Aplicar&query=leprosy>+

<https://repositorio.ufsc.br/handle/123456789/74645/discover?filtertype_1=title&filter_relational_operator_1=contains&filter_1=leprosy&filtertype_2=title&filter_relational_operator_2=contains&filter_2=clofazimine&submit_apply_filter=Aplicar&query=leprosy>+

94) Universidade do Estado de Santa Catarina

<https://pergamumweb.udesc.br/biblioteca/index.php>

95) Universidade do Vale do Itajaí

<https://eds.s.ebscohost.com/eds/results?vid=3&sid=1376ae56-49e2-4828-9396-60b6e23b570f%40redis&bquery=leprosy&bdata=JmNsaTA9RlQxJmNsdjA9WSZsYW5nPXB0LWJyJnR5cGU9MCZzZWFyY2hNb2RlPUFuZCZzaXRlPWVkcy1saXZlJnNjb3BlPXNpdGU%3d>

<https://eds.s.ebscohost.com/eds/results?vid=4&sid=1376ae56-49e2-4828-9396-60b6e23b570f%40redis&bquery=leprosy+and+clofazimine&bdata=JmNsaTA9RlQxJmNsdjA9WSZsYW5nPXB0LWJyJnR5cGU9MCZzZWFyY2hNb2RlPUFuZCZzaXRlPWVkcy1saXZlJnNjb3BlPXNpdGU%3d>

96) Universidade Regional de Blumenau

<https://bu.furb.br/consulta/novaConsulta/pesqPosGrad.php?acao=pesquisar&rdbtTpConsulta=10&ExpBusca=Leprosy&anoDefesaIni=--Todos+os+anos--&anoDefesaFim=--Todos+os+anos--&progPG=--Todos+os+programas--&Submit=Pesquisar>

<https://bu.furb.br/consulta/novaConsulta/pesqPosGrad.php?acao=pesquisar&rdbtTpConsulta=10&ExpBusca=leprosy+and+clofazimine&anoDefesaIni=--Todos+os+anos--&anoDefesaFim=--Todos+os+anos--&progPG=--Todos+os+programas--&Submit=Pesquisar>

97) Universidade Federal de Sergipe

<https://ri.ufs.br/handle/riufs/2145/browse?type=subject&order=ASC&rpp=20&value=Leprosy>

<https://ri.ufs.br/handle/riufs/2145/browse?type=subject&sort_by=1&order=ASC&rpp=20&etal=-1&value=Leprosy&starts_with=leprosy+and+clofazimine>

**Table 2S-A.** Complete search strategy of clarithromycin review in general databases and register clinical trials databases

| **MEDLINE via Pubmed: pubmed.ncbi.nlm.nih.gov** |
| --- |
| **Results: 90** |
| (((((((((((("Leprosy"[Mesh]) OR (Hansen's Disease[Text Word])) OR (Hansen Disease[Text Word])) OR (Leprosy[Text Word])) OR ((((((((((((("Leprosy, Paucibacillary"[Mesh]) OR (Leprosy, Paucibacillary[Text Word])) OR (Leprosies, Paucibacillary[Text Word])) OR (Paucibacillary Leprosies[Text Word])) OR (Indeterminate Tuberculoid[Text Word])) OR (Indeterminate Tuberculoids[Text Word])) OR (Tuberculoid, Indeterminate[Text Word])) OR (Tuberculoids, Indeterminate[Text Word])) OR (Paucibacillary Leprosy[Text Word])) OR (Borderline Tuberculoid[Text Word])) OR (Borderline Tuberculoids[Text Word])) OR (Tuberculoid, Borderline[Text Word])) OR (Tuberculoids, Borderline[Text Word]))) OR (("Mycobacterium leprae"[Mesh]) OR (Mycobacterium leprae[Text Word]))) OR ((((((((((((("Leprosy, Tuberculoid"[Mesh]) OR (Leprosy, Tuberculoid[Text Word])) OR (Leprosies, Tuberculoid[Text Word])) OR (Tuberculoid Leprosies[Text Word])) OR (Tuberculoid Leprosy[Text Word])) OR (Leprosy, Neural[Text Word])) OR (Leprosies, Neural[Text Word])) OR (Neural Leprosies[Text Word])) OR (Neural Leprosy[Text Word])) OR (Leprosy, Macular[Text Word])) OR (Leprosies, Macular[Text Word])) OR (Macular Leprosies[Text Word])) OR (Macular Leprosy[Text Word]))) OR ((((((((((((("Leprosy, Lepromatous"[Mesh]) OR (Leprosy, Lepromatous[Text Word])) OR (Lepromatous Leprosies[Text Word])) OR (Lepromatous Leprosy[Text Word])) OR (Leprosies, Lepromatous[Text Word])) OR (Leprosy, Cutaneous[Text Word])) OR (Cutaneous Leprosies[Text Word])) OR (Cutaneous Leprosy[Text Word])) OR (Leprosies, Cutaneous[Text Word])) OR (Leprosy, Nodular[Text Word])) OR (Leprosies, Nodular[Text Word])) OR (Nodular Leprosies[Text Word])) OR (Nodular Leprosy[Text Word]))) OR ((((((((("Leprosy, Borderline"[Mesh]) OR (Leprosy, Borderline[Text Word])) OR (Borderline Leprosies[Text Word])) OR (Borderline Leprosy[Text Word])) OR (Leprosies, Borderline[Text Word])) OR (Leprosy, Dimorphous[Text Word])) OR (Dimorphous Leprosies[Text Word])) OR (Dimorphous Leprosy[Text Word])) OR (Leprosies, Dimorphous[Text Word]))) OR ((((((((("Leprosy, Multibacillary"[Mesh]) OR (Leprosy, Multibacillary[Text Word])) OR (Leprosies, Multibacillary[Text Word])) OR (Multibacillary Leprosies[Text Word])) OR (Midborderline Lepromatous[Text Word])) OR (Lepromatous, Midborderline[Text Word])) OR (Multibacillary Leprosy[Text Word])) OR (Borderline Lepromatous[Text Word])) OR (Lepromatous, Borderline[Text Word]))) ) OR (Lepros*[Text Word]))  AND  (((((((((("Clarithromycin"[Mesh]) OR (Clarithromycin[Text Word])) OR (6-O-Methylerythromycin[Text Word])) OR (TE-031[Text Word])) OR (TE 031[Text Word])) OR (TE031[Text Word])) OR (A-56268[Text Word])) OR (A 56268[Text Word])) OR (A56268[Text Word])) OR (Biaxin[Text Word])) |
| **EMBASE: embase.com** |
| **Results: 466** |
| ('m. leprae infection'/exp OR 'm. leprae infection' OR 'mycobacterium leprae infection'/exp OR 'mycobacterium leprae infection' OR 'elephantiasis graecorum'/exp OR 'elephantiasis graecorum' OR 'hansen disease'/exp OR 'hansen disease' OR 'hanseniasis'/exp OR 'hanseniasis' OR 'infection by mycobacterium leprae'/exp OR 'infection by mycobacterium leprae' OR 'infection of mycobacterium leprae'/exp OR 'infection of mycobacterium leprae' OR 'lepra'/exp OR 'lepra' OR 'leprology'/exp OR 'leprology' OR 'leprosis'/exp OR 'leprosis' OR 'leprosy'/exp OR 'leprosy' OR 'leprous infection'/exp OR 'leprous infection' OR 'morbus hansen'/exp OR 'morbus hansen' OR 'border line leprosy'/exp OR 'border line leprosy' OR 'borderline lepromatous'/exp OR 'borderline lepromatous' OR 'borderline leprosy'/exp OR 'borderline leprosy' OR 'dimorphous leprosy'/exp OR 'dimorphous leprosy' OR 'leprosy, borderline'/exp OR 'leprosy, borderline' OR 'midborderline leprosy'/exp OR 'midborderline leprosy' OR 'lepromatous lepra'/exp OR 'lepromatous lepra' OR 'lepromatous leprosy'/exp OR 'lepromatous leprosy' OR 'leprosy, lepromatous'/exp OR 'leprosy, lepromatous' OR 'leprosy, tuberculoid'/exp OR 'leprosy, tuberculoid' OR 'tuberculoid lepra'/exp OR 'tuberculoid lepra' OR 'tuberculoid leprosy'/exp OR 'tuberculoid leprosy' OR 'mb leprosy'/exp OR 'mb leprosy' OR 'leprosy, multibacillary'/exp OR 'leprosy, multibacillary' OR 'multibacillary leprosy'/exp OR 'multibacillary leprosy' OR 'pb leprosy'/exp OR 'pb leprosy' OR 'leprosy, paucibacillary'/exp OR 'leprosy, paucibacillary' OR 'paucibacillary leprosy'/exp OR 'paucibacillary leprosy' OR 'mycobacterium leprae'/exp OR 'mycobacterium leprae' OR 'bacillus hansen'/exp OR 'bacillus hansen' OR 'bacillus leprae'/exp OR 'bacillus leprae' OR 'hansen bacillus'/exp OR 'hansen bacillus' OR 'icrc bacillus'/exp OR 'icrc bacillus' OR 'leprosy bacillus'/exp OR 'leprosy bacillus' OR 'mycobacterium hansenii'/exp OR 'mycobacterium hansenii' OR 'mycoplasma leprae'/exp OR 'mycoplasma leprae')  AND  ('6 [4 (dimethylamino) 3 hydroxy 6 methyl 2 oxanyl] oxy 14 ethyl 12, 13 dihydroxy 4 [5 hydroxy 4 methoxy 4, 6 dimethyl 2 oxanyl] oxy 7 methoxy 3, 5, 7, 9, 11, 13 hexamethyl oxacyclotetradecane 2, 10 dione'/exp OR '6 [4 (dimethylamino) 3 hydroxy 6 methyl 2 oxanyl] oxy 14 ethyl 12, 13 dihydroxy 4 [5 hydroxy 4 methoxy 4, 6 dimethyl 2 oxanyl] oxy 7 methoxy 3, 5, 7, 9, 11, 13 hexamethyl oxacyclotetradecane 2, 10 dione' OR '6 [4 (dimethylamino) 3 hydroxy 6 methyloxan 2 yl] oxy 14 ethyl 12, 13 dihydroxy 4 [5 hydroxy 4 methoxy 4, 6 dimethyloxan 2 yl] oxy 7 methoxy 3, 5, 7, 9, 11, 13 hexamethyl oxacyclotetradecane 2, 10 dione'/exp OR '6 [4 (dimethylamino) 3 hydroxy 6 methyloxan 2 yl] oxy 14 ethyl 12, 13 dihydroxy 4 [5 hydroxy 4 methoxy 4, 6 dimethyloxan 2 yl] oxy 7 methoxy 3, 5, 7, 9, 11, 13 hexamethyl oxacyclotetradecane 2, 10 dione' OR '6 o methylerythromycin a'/exp OR '6 o methylerythromycin a' OR 'a 56268'/exp OR 'a 56268' OR 'a56268'/exp OR 'a56268' OR 'abbotic'/exp OR 'abbotic' OR 'abbotic xl'/exp OR 'abbotic xl' OR 'abbott 56268'/exp OR 'abbott 56268' OR 'aeroxina'/exp OR 'aeroxina' OR 'bactirel'/exp OR 'bactirel' OR 'baxin filmtab'/exp OR 'baxin filmtab' OR 'biaxin'/exp OR 'biaxin' OR 'biaxin hp'/exp OR 'biaxin hp' OR 'biaxin xl'/exp OR 'biaxin xl' OR 'biaxin xl-pak'/exp OR 'biaxin xl-pak' OR 'biclar'/exp OR 'biclar' OR 'biclar forte'/exp OR 'biclar forte' OR 'biclar iv'/exp OR 'biclar iv' OR 'biclar kids'/exp OR 'biclar kids' OR 'biclar uno'/exp OR 'biclar uno' OR 'bicrolid'/exp OR 'bicrolid' OR 'binoklar'/exp OR 'binoklar' OR 'bremon'/exp OR 'bremon' OR 'brevil od'/exp OR 'brevil od' OR 'c-clarin'/exp OR 'c-clarin' OR 'carimycin'/exp OR 'carimycin' OR 'celex'/exp OR 'celex' OR 'clacin'/exp OR 'clacin' OR 'clacine'/exp OR 'clacine' OR 'clambiotic'/exp OR 'clambiotic' OR 'clapharma'/exp OR 'clapharma' OR 'clari'/exp OR 'clari' OR 'claribid'/exp OR 'claribid' OR 'claridar'/exp OR 'claridar' OR 'clarikan'/exp OR 'clarikan' OR 'clarimac'/exp OR 'clarimac' OR 'claripen'/exp OR 'claripen' OR 'clarith'/exp OR 'clarith' OR 'clarithromycin'/exp OR 'clarithromycin' OR 'clarithromycin extended release'/exp OR 'clarithromycin extended release' OR 'clarithromycin lactobionate'/exp OR 'clarithromycin lactobionate' OR 'clarithromycina'/exp OR 'clarithromycina' OR 'clarithromycine'/exp OR 'clarithromycine' OR 'claritrol'/exp OR 'claritrol' OR 'claroma'/exp OR 'claroma' OR 'clormicin'/exp OR 'clormicin' OR 'crixan'/exp OR 'crixan' OR 'cylind'/exp OR 'cylind' OR 'cyllind'/exp OR 'cyllind' OR 'dicupal'/exp OR 'dicupal' OR 'er 36469'/exp OR 'er 36469' OR 'er36469'/exp OR 'er36469' OR 'erythromycin a, 6 o methyl'/exp OR 'erythromycin a, 6 o methyl' OR 'gervaken'/exp OR 'gervaken' OR 'hecobac'/exp OR 'hecobac' OR 'heliclar'/exp OR 'heliclar' OR 'helitic'/exp OR 'helitic' OR 'klacid'/exp OR 'klacid' OR 'klacid baby'/exp OR 'klacid baby' OR 'klacid forte'/exp OR 'klacid forte' OR 'klacid iv'/exp OR 'klacid iv' OR 'klacid kid'/exp OR 'klacid kid' OR 'klacid la'/exp OR 'klacid la' OR 'klacid od'/exp OR 'klacid od' OR 'klacid paediatric'/exp OR 'klacid paediatric' OR 'klacid pro'/exp OR 'klacid pro' OR 'klacid rm'/exp OR 'klacid rm' OR 'klacid saft'/exp OR 'klacid saft' OR 'klacid sr'/exp OR 'klacid sr' OR 'klacid uno'/exp OR 'klacid uno' OR 'klacid xl'/exp OR 'klacid xl' OR 'klacina'/exp OR 'klacina' OR 'klaciped'/exp OR 'klaciped' OR 'klaribac'/exp OR 'klaribac' OR 'klaricid'/exp OR 'klaricid' OR 'klaricid adult'/exp OR 'klaricid adult' OR 'klaricid h.p.'/exp OR 'klaricid h.p.' OR 'klaricid o.d.'/exp OR 'klaricid o.d.' OR 'klaricid paediatric'/exp OR 'klaricid paediatric' OR 'klaricid pediatric'/exp OR 'klaricid pediatric' OR 'klaricid xl'/exp OR 'klaricid xl' OR 'klaridex'/exp OR 'klaridex' OR 'klaridia'/exp OR 'klaridia' OR 'klarin'/exp OR 'klarin' OR 'klerimed'/exp OR 'klerimed' OR 'kofron'/exp OR 'kofron' OR 'lagur'/exp OR 'lagur' OR 'lekoklar'/exp OR 'lekoklar' OR 'macladim'/exp OR 'macladim' OR 'macladin'/exp OR 'macladin' OR 'macladin rm'/exp OR 'macladin rm' OR 'maclar'/exp OR 'maclar' OR 'macrobiol'/exp OR 'macrobiol' OR 'macrobiol s.r.'/exp OR 'macrobiol s.r.' OR 'makcin'/exp OR 'makcin' OR 'maksin sr'/exp OR 'maksin sr' OR 'mavid'/exp OR 'mavid' OR 'monoclarium'/exp OR 'monoclarium' OR 'monozeclar'/exp OR 'monozeclar' OR 'naxy'/exp OR 'naxy' OR 'soriclar'/exp OR 'soriclar' OR 'soriclar rm'/exp OR 'soriclar rm' OR 'te 031'/exp OR 'te 031' OR 'te031'/exp OR 'te031' OR 'veclam'/exp OR 'veclam' OR 'winclar'/exp OR 'winclar' OR 'zeclar'/exp OR 'zeclar' OR 'zeclar od'/exp OR 'zeclar od')  AND  [embase]/lim |
| **BVS: bvsalud.org** |
| **Results: 36** |
| (((mh:(hanseníase)) OR (doença de hansen) OR (lepra) OR (reação hansênica reversa) OR (reação hansênica tipo 1) OR (reação hansênica tipo 2) OR (reação hansênica do tipo eritema nodoso) OR (reações hansênicas) OR (mh:(leprosy)) OR (hansen disease) OR (hansen's disease) OR (mh:(lepra)) OR (enfermedad de hansen) OR (mal de hansen) OR (exc01.150.252.410.040.552.475.371*) OR (exsp4.012.148.164*)) OR ((mh:(hanseníase multibacilar)) OR (hanseníase dimorfa-dimorfa) OR (hanseníase dimorfa-virchowiana) OR (mh:(leprosy, multibacillary)) OR (borderline lepromatous) OR (lepromatous, borderline) OR (lepromatous, midborderline) OR (leprosies, multibacillary) OR (midborderline lepromatous) OR (multibacillary leprosies) OR (multibacillary leprosy) OR (mh:(lepra multibacilar)) OR (lepromatosa intermedia) OR (lepromatosa limítrofe) OR (exc01.150.252.410.040.552.475.371.775*)) OR ((mh:(hanseníase paucibacilar)) OR (tuberculoide indeterminada) OR (tuberculoide limítrofe) OR (exc01.150.252.410.040.552.475.371.850*) OR (mh:(leprosy paucibacillary)) OR (borderline tuberculoid) OR (borderline tuberculoids) OR (indeterminate tuberculoid) OR (indeterminate tuberculoids) OR (leprosies, paucibacillary) OR (paucibacillary leprosies) OR (paucibacillary leprosy) OR (tuberculoid, borderline) OR (tuberculoid, indeterminate) OR (tuberculoids, borderline) OR (tuberculoids, indeterminate) OR (mh:(lepra paucibacilar)) OR (tuberculoide indeterminada) OR (tuberculoide limítrofe)) OR ((mh:(hanseníase tuberculoide)) OR (hanseníase macular) OR (hanseníase neurítica) OR (exc01.150.252.410.040.552.475.371.850.500*) OR (mh:(leprosy, tuberculoid)) OR (leprosies, macular) OR (leprosies, neural) OR (leprosies, tuberculoid) OR (leprosy, macular) OR (leprosy, neural) OR (macular leprosies) OR (macular leprosy) OR (neural leprosies) OR (neural leprosy) OR (tuberculoid leprosies) OR (tuberculoid leprosy) OR (mh:(lepra tuberculoide)) OR (lepra macular) OR (lepra neural)) OR ((mh:(hanseníase dimorfa)) OR (exc01.150.252.410.040.552.475.371.850.249*) OR (mh:(leprosy borderline)) OR (borderline leprosies) OR (borderline leprosy) OR (dimorphous leprosies) OR (dimorphous leprosy) OR (leprosies, borderline) OR (leprosies, dimorphous) OR (leprosy, dimorphous) OR (mh:(lepra dimorfa)) OR (lepra incierta)) OR ((mh:(hanseníase virchowiana)) OR (hanseníase cutânea) OR (hanseníase nodular) OR (exc01.150.252.410.040.552.475.371.775.500*) OR (mh:(leprosy, lepromatous)) OR (cutaneous leprosies) OR (cutaneous leprosy) OR (lepromatous leprosies) OR (lepromatous leprosy) OR (leprosies, cutaneous) OR (leprosies, lepromatous) OR (leprosies, nodular) OR (leprosy, cutaneous) OR (leprosy, nodular) OR (nodular leprosies) OR (nodular leprosy) OR (mh:(lepra lepromatosa)) OR (lepra cutánea) OR (lepra nodular)) OR ((mh:(mycobacterium leprae)) OR (bacilo de hansen) OR (bacilo de la hanseniasis) OR (bacilo de la lepra) OR (exb03.510.024.962.500.502*) OR (exb03.510.460.400.410.552.552.502*) OR (bacilo da hanseníase) OR (bacilo de hansen)))  AND  ((mh:(claritromicina)) OR (exd02.540.576.500.992.100*) OR (mh:(clarithromycin)) OR (6-o-methylerythromycin) OR (a 56268) OR (a-56268) OR (a56268) OR (biaxin) OR (te 031) OR (te-031) OR (te031)) |
| **LILACS via BVS: lilacs.bvsalud.org** |
| **Results: 4** |
| (((mh:(hanseníase)) OR (doença de hansen) OR (lepra) OR (reação hansênica reversa) OR (reação hansênica tipo 1) OR (reação hansênica tipo 2) OR (reação hansênica do tipo eritema nodoso) OR (reações hansênicas) OR (mh:(leprosy)) OR (hansen disease) OR (hansen's disease) OR (mh:(lepra)) OR (enfermedad de hansen) OR (mal de hansen) OR (exc01.150.252.410.040.552.475.371*) OR (exsp4.012.148.164*)) OR ((mh:(hanseníase multibacilar)) OR (hanseníase dimorfa-dimorfa) OR (hanseníase dimorfa-virchowiana) OR (mh:(leprosy, multibacillary)) OR (borderline lepromatous) OR (lepromatous, borderline) OR (lepromatous, midborderline) OR (leprosies, multibacillary) OR (midborderline lepromatous) OR (multibacillary leprosies) OR (multibacillary leprosy) OR (mh:(lepra multibacilar)) OR (lepromatosa intermedia) OR (lepromatosa limítrofe) OR (exc01.150.252.410.040.552.475.371.775*)) OR ((mh:(hanseníase paucibacilar)) OR (tuberculoide indeterminada) OR (tuberculoide limítrofe) OR (exc01.150.252.410.040.552.475.371.850*) OR (mh:(leprosy paucibacillary)) OR (borderline tuberculoid) OR (borderline tuberculoids) OR (indeterminate tuberculoid) OR (indeterminate tuberculoids) OR (leprosies, paucibacillary) OR (paucibacillary leprosies) OR (paucibacillary leprosy) OR (tuberculoid, borderline) OR (tuberculoid, indeterminate) OR (tuberculoids, borderline) OR (tuberculoids, indeterminate) OR (mh:(lepra paucibacilar)) OR (tuberculoide indeterminada) OR (tuberculoide limítrofe)) OR ((mh:(hanseníase tuberculoide)) OR (hanseníase macular) OR (hanseníase neurítica) OR (exc01.150.252.410.040.552.475.371.850.500*) OR (mh:(leprosy, tuberculoid)) OR (leprosies, macular) OR (leprosies, neural) OR (leprosies, tuberculoid) OR (leprosy, macular) OR (leprosy, neural) OR (macular leprosies) OR (macular leprosy) OR (neural leprosies) OR (neural leprosy) OR (tuberculoid leprosies) OR (tuberculoid leprosy) OR (mh:(lepra tuberculoide)) OR (lepra macular) OR (lepra neural)) OR ((mh:(hanseníase dimorfa)) OR (exc01.150.252.410.040.552.475.371.850.249*) OR (mh:(leprosy borderline)) OR (borderline leprosies) OR (borderline leprosy) OR (dimorphous leprosies) OR (dimorphous leprosy) OR (leprosies, borderline) OR (leprosies, dimorphous) OR (leprosy, dimorphous) OR (mh:(lepra dimorfa)) OR (lepra incierta)) OR ((mh:(hanseníase virchowiana)) OR (hanseníase cutânea) OR (hanseníase nodular) OR (exc01.150.252.410.040.552.475.371.775.500*) OR (mh:(leprosy, lepromatous)) OR (cutaneous leprosies) OR (cutaneous leprosy) OR (lepromatous leprosies) OR (lepromatous leprosy) OR (leprosies, cutaneous) OR (leprosies, lepromatous) OR (leprosies, nodular) OR (leprosy, cutaneous) OR (leprosy, nodular) OR (nodular leprosies) OR (nodular leprosy) OR (mh:(lepra lepromatosa)) OR (lepra cutánea) OR (lepra nodular)) OR ((mh:(mycobacterium leprae)) OR (bacilo de hansen) OR (bacilo de la hanseniasis) OR (bacilo de la lepra) OR (exb03.510.024.962.500.502*) OR (exb03.510.460.400.410.552.552.502*) OR (bacilo da hanseníase) OR (bacilo de hansen)))  AND  ((mh:(claritromicina)) OR (exd02.540.576.500.992.100*) OR (mh:(clarithromycin)) OR (6-o-methylerythromycin) OR (a 56268) OR (a-56268) OR (a56268) OR (biaxin) OR (te 031) OR (te-031) OR (te031))  AND  ( db:("LILACS")) |
| **Cochrane Library: cochranelibrary.com** |
| **Results: 39** |
| #1 MeSH descriptor: [Leprosy] explode all trees 298  #2 Hansen Disease 1693  #3 Hansen's Disease 1693  #4 Leprosy 863  #5 MeSH descriptor: [Leprosy, Borderline] explode all trees 20  #6 Leprosy, Dimorphous 0  #7 Leprosies, Borderline 0  #8 Borderline Leprosy 56  #9 Dimorphous Leprosy 0  #10 Borderline Leprosies 0  #11 Dimorphous Leprosies 0  #12 Leprosies, Dimorphous 0  #13 MeSH descriptor: [Leprosy, Multibacillary] explode all trees 53  #14 Multibacillary Leprosies 0  #15 Lepromatous, Borderline 43  #16 Midborderline Lepromatous 2  #17 Borderline Lepromatous 43  #18 Leprosies, Multibacillary 0  #19 Multibacillary Leprosy 122  #20 Lepromatous, Midborderline 2  #21 MeSH descriptor: [Leprosy, Paucibacillary] explode all trees 31  #22 Paucibacillary Leprosies 0  #23 Indeterminate Tuberculoid 4  #24 Indeterminate Tuberculoids 0  #25 Leprosies, Paucibacillary 0  #26 Tuberculoids, Indeterminate 0  #27 Tuberculoid, Indeterminate 4  #28 Borderline Tuberculoid 19  #29 Borderline Tuberculoids 0  #30 Tuberculoid, Borderline 19  #31 Tuberculoids, Borderline 0  #32 Paucibacillary Leprosy 66  #33 MeSH descriptor: [Leprosy, Tuberculoid] explode all trees 10  #34 Leprosy, Macular 5  #35 Leprosies, Macular 0  #36 Macular Leprosies 0  #37 Macular Leprosy 5  #38 Neural Leprosy 23  #39 Leprosies, Neural 0  #40 Leprosy, Neural 23  #41 Neural Leprosies 0  #42 Leprosies, Tuberculoid 0  #43 Tuberculoid Leprosy 34  #44 Tuberculoid Leprosies 0  #45 MeSH descriptor: [Leprosy, Lepromatous] explode all trees 47  #46 Lepromatous Leprosies 0  #47 Lepromatous Leprosy 142  #48 Leprosies, Lepromatous 0  #49 Leprosies, Cutaneous 0  #50 Nodular Leprosy 5  #51 Leprosy, Cutaneous 55  #52 Nodular Leprosies 0  #53 Cutaneous Leprosy 55  #54 Cutaneous Leprosies 0  #55 Leprosies, Nodular 0  #56 Leprosy, Nodular 5  #57 MeSH descriptor: [Mycobacterium leprae] explode all trees 67  #58 {OR #1-#57} 2535  #59 MeSH descriptor: [Clarithromycin] explode all trees 1519  #60 TE-031 7  #61 TE031 2  #62 TE 031 731  #63 A56268 2  #64 A 56268 7  #65 A-56268 4  #66 Biaxin 40  #67 {OR #59-#66} 2267  #68 #58 AND #67 39 |
| **Clinicaltrials.gov** |
| **Results: 0** |
| Leprosy AND clarithromycin |
| **International Clinical Trials Registry Platform: who.int/clinical-trials-registry-platform** |
| **Results: 2** |
| Leprosy AND clarithromycin |
| **OpenGrey via Data Archiving and Network Services (DANS): easy.dans.knaw.nl/ui/datasets/id/easy-dataset:200362** |
| **Results: 0** |
| Leprosy AND clarithromycin |
| **Web of Science: https://www-webofscience.ez54.periodicos.capes.gov.br/wos/woscc/basic-search** |
| **Results: 111** |
| (((((((((((ALL=(leprosy)) OR ALL=(Hansen's Disease)) OR ALL=(Paucibacillary leprosis)) OR ALL=(Mycobacterium leprae)) OR ALL=(Tuberculoid Leprosy)) OR ALL=(Macular leprosis)) OR ALL=(Lepromatous Leprosy)) OR ALL=(Cutaneous Leprosy)) OR ALL=(Borderline Leprosy)) OR ALL=(dimorphus leprosis)) OR ALL=(Multibacillary leprosis)) AND ALL=(clarithromycin ) |
| **Scopus: scopus. com** |
| **Results: 1,385** |
| ( ALL ( lepros* ) OR ALL ( "hansen* disease" ) OR ALL ( "mycobacterium leprae" ) OR ALL ( "M. leprae" ) AND ALL ( clarithromycin ) ) |

**Thesis databases searched (Clarithromycin review)**

1) Queen’s Univerity Library Catalogue

<https://ocul-qu.primo.exlibrisgroup.com/discovery/search?query=any,contains,leprosy&tab=Everything&search_scope=MyInst_and_CI&vid=01OCUL_QU:QU_DEFAULT&facet=rtype,include,dissertations&offset=0>

<https://ocul-qu.primo.exlibrisgroup.com/discovery/search?query=any,contains,Clarithromycin%20and%20leprosy&tab=Everything&search_scope=MyInst_and_CI&vid=01OCUL_QU:QU_DEFAULT&facet=rtype,include,dissertations&offset=40&came_from=pagination_4_5>

2) Queen’s Univerity Research & Learning Repository (QSpace): Theses & Dissertations

<https://qspace.library.queensu.ca/discover?scope=%2F&query=leprosy+&submit=>

<https://qspace.library.queensu.ca/discover?scope=1974%2F196&query=leprosy+and+Clarithromycin&submit=>

3) Theses Canada Portal

<https://www.bac-lac.gc.ca/eng/services/theses/Pages/list.aspx?AW_S=leprosy>

<https://www.bac-lac.gc.ca/eng/services/theses/Pages/list.aspx?TW_S=leprosy+and+Clarithromycin>

4) The Directory of Open Access Repositories

<http://v2.sherpa.ac.uk/cgi/search/repository/basic?repository_name-auto=leprosy&_action_search=Search&screen=Search&repository_name-auto_merge=ALL>

https://v2.sherpa.ac.uk/cgi/search/repository/basic?repository_name-auto=leprosy+and+Clarithromycin&_action_search=Search&screen=Search&repository_name-auto_merge=ALL

5) ProQuest Open Access Dissertations and Theses

<https://www.proquest.com/resultsol/61E9B086CA3D4659PQ/1>

<https://www.proquest.com/resultsol/CCD3D0CB22C745E4PQ/1>

6) OpenThesis.org

7) PhdData.org

8) Center for Research Libraries

<http://catalog.crl.edu/search/Y?SEARCH=leprosy&searchscope=4&x=6&y=15>

<http://catalog.crl.edu/search~S4?/Yleprosy+clarithromycin&searchscope=4&SORT=DZ/Yleprosy+and+clarithromycin&searchscope=4&SORT=DZ&extended=0&SUBKEY=leprosy+and+clarithromycin/51%2C56%2C56%2CB/browse>

9) Networked Digital Library of Theses and Dissertations

<http://search.ndltd.org/search.php?q=leprosy&source_set_names=&year_start=&year_end=>

<http://search.ndltd.org/search.php?q=leprosy+and+clarithromycin&source_set_names=&year_start=&year_end=>

10) National Library of Australia’s Trove Service

<https://trove.nla.gov.au/book/result?l-format=Thesis&q=leprosy>

<https://trove.nla.gov.au/search/category/books?keyword=leprosy%20and%20clarithromycin>

11) United Kingdom E-Theses Online Service

<https://ethos.bl.uk/SearchResults.do>

12) DART-Europe

<https://www.dart-europe.org/basic-results.php?kw%5B%5D=leprosy&f=n>

<https://www.dart-europe.org/basic-results.php?kw%5B%5D=leprosy+and+clarithromycin&f=n>

13) Système universitaire de documentation

[http://www.sudoc.abes.fr/cbs/xslt//DB=2.1/SET=1/TTL=1/CMD?ACT=SRCHA&IKT=1016&SRT=RLV&TRM=leprosy](http://www.sudoc.abes.fr/cbs/xslt/DB=2.1/SET=1/TTL=1/CMD?ACT=SRCHA&IKT=1016&SRT=RLV&TRM=leprosy)

<http://www.sudoc.abes.fr/cbs/xslt//DB=2.1/SET=2/TTL=1/CMD?ACT=SRCHA&IKT=1016&SRT=RLV&TRM=leprosy+and+clarithromycin>

14) Fichier central des thèses

<http://theses.fr/fr/?q=leprosy+>

<http://theses.fr/fr/?q=leprosy+and+clarithromycin>

15) Deutschen National Bibliothek

<https://portal.dnb.de/opac/simpleSearch?query=leprosy>+

<https://portal.dnb.de/opac/simpleSearch?query=leprosy+and+Clarithromycin>

16) Bibliotecario de la Universidad Nacional Autónoma de México

<https://eds.s.ebscohost.com/eds/resultsadvanced?vid=2&sid=0984d99a-3cc3-4789-88a9-cd1f498e6158%40redis&bquery=leprosy&bdata=JmNsaTA9RlQmY2x2MD1ZJmxhbmc9ZXMmdHlwZT0xJnNlYXJjaE1vZGU9U3RhbmRhcmQmc2l0ZT1lZHMtbGl2ZQ%3d%3d>

<https://eds.s.ebscohost.com/eds/resultsadvanced?vid=4&sid=0984d99a-3cc3-4789-88a9-cd1f498e6158%40redis&bquery=leprosy+AND+clarithromycin&bdata=JmNsaTA9RlQmY2x2MD1ZJmxhbmc9ZXMmdHlwZT0xJnNlYXJjaE1vZGU9U3RhbmRhcmQmc2l0ZT1lZHMtbGl2ZQ%3d%3d>

17) National Academic Research and Collaborations Information System

<https://www.narcis.nl/search/uquery/leprosy/Language/EN>

<https://www.narcis.nl/search/uquery/leprosy%20and%20clarithromycin/Language/EN>

18) Spain’s Ministry of Education thesis database

<https://administracion.gob.es/buscador/buscadorSimple.htm?q=leprosy&site=PAG_ES&filter=p&busqueda=true&buscar=Buscar&buscar=#gsc.tab=0&gsc.q=leprosy&gsc.page=1>

<https://administracion.gob.es/buscador/buscadorSimple.htm?q=leprosy+and+clarithromycin&site=PAG_ES&filter=p&busqueda=true&buscar=Buscar&buscar=#gsc.tab=0&gsc.q=leprosy%20and%20clarithromycin&gsc.page=1>

19) Biblioteca Virtual Miguel de Cervantes

<http://www.cervantesvirtual.com/buscador/?q=leprosy>

<https://www.cervantesvirtual.com/buscador/?q=leprosy+and+clarithromycin>

20) Universidad Complutense de Madrid’s catalogue

<https://ucm.on.worldcat.org/search?databaseList=&queryString=leprosy>

<https://ucm.on.worldcat.org/search?queryString=leprosy%20and%20clarithromycin&databaseList=&clusterResults=false&groupVariantRecords=true&database=Z-dissert&changedFacet=database>

21) Tesis Doctorals en Xarxa

<https://tdx.cat/discover?scope=%2F&query=leprosy&submit=>

<https://tdx.cat/discover?scope=%2F&query=leprosy+and+clarithromycin&submit=&scope=%2F>

22) Swiss National Library

<https://www.nb.admin.ch/snl/de/home/suche.html#leprosy>

<https://www.nb.admin.ch/snl/de/home/suche.html#leprosy%20and%20clarithromycin>

23) Universidade de São Paulo

<https://teses.usp.br/index.php?option=com_jumi&fileid=12&Itemid=77&lang=pt-br&filtro=leprosy>

<https://teses.usp.br/index.php?option=com_jumi&fileid=12&Itemid=77&lang=pt-br&filtro=leprosy%20and%20clarithromycin>

24) Universidade Federal de São Paulo

<https://repositorio.unifesp.br/discover?query=leprosy&filtertype=type&filter_relational_operator=equals&filter=Tese+de+doutorado>

<https://repositorio.unifesp.br/discover?scope=%2F&query=leprosy+and+clarithromycin&submit=&filtertype_0=type&filter_relational_operator_0=equals&filter_0=Tese+de+doutorado>

25) Universidade Estadual de Campinas

<http://www.bibliotecadigital.unicamp.br/document/list_index.php?palavra=leprosy&type_list=3&tid=498&stid%5B%5D=0&sent=s>

<http://www.bibliotecadigital.unicamp.br/cgi-bin/search.cgi?q=lleprosy+and+clarithromycin&fl=m&ps=25&uid=0&lg=pt_BR&wf=0>

26) Universidade Estadual Paulista

<https://repositorio.unesp.br/handle/11449/77291/discover?query=leprosy+&submit=>

<https://repositorio.unesp.br/handle/11449/77291/discover?query=leprosy+and+clarithromycin&submit=>

27) Pontifícia Universidade Católica de Campinas

<https://itms.libsteps.com/PUCC/index.php/result?charset=utf-8&category1=0&category2=0&category3=0&text1=leprosy&text2=&text3=&op=0&op2=0&year1=&year2=&dbnum=78661+64001+92851+54551+34771+39871+1401+3371+63601+78881+63671+65231+78651+75281&display=30&recstart=1&sort=rel&id=164808612944#/?recstart=1&display=20&dbnum=78651&key=&type=search&reload=off>

<https://itms.libsteps.com/PUCC/index.php/result?id=164808627314414&category1=0&category2=0&category3=0&text1=leprosy%20and%20clarithromycin&text2=&text3=&op=0&op2=0&year1=&year2=&dbnum=78661%2064001%2092851%2054551%2034771%2039871%201401%203371%2063601%2078881%2063671%2065231%2078651%2075281&display=30&recstart=1&sort=rel#/?recstart=1&display=20&dbnum=78651&key=&type=search&reload=off>

28) Universidade Católica de Santos

<http://biblioteca.unisantos.br/pergamum/biblioteca/>

29) Pontifícia Universidade Católica de São Paulo

<https://sapientia.pucsp.br/simple-search?query=leprosy>

<https://sapientia.pucsp.br/simple-search?query=leprosy+and+clarithromycin>

30) Universidade de Guarulhos

<http://tede.ung.br/simple-search?query=leprosy>

<http://tede.ung.br/simple-search?query=leprosy+clarithromycin>

31) Universidade de Taubaté

<https://unitau.br/busca/?termo=leprosy>

<https://unitau.br/busca/?termo=leprosy+and+clarithromycin>

32) Universidade do Oeste Paulista

<https://www.unoeste.br/Biblioteca>

33) Universidade Presbiteriana Mackenzie

<http://tede.mackenzie.br/jspui/simple-search?query=leprosy>

<http://tede.mackenzie.br/jspui/simple-search?location=%2F&query=leprosy+and+clarithromycin&rpp=10&sort_by=score&order=desc>

34) Universidade Metodista de São Paulo

<http://portal.metodista.br/posreligiao/publicacoes/teses-e-dissertacoes/teses-de-doutorado-1993-a-2003-1>

<http://portal.metodista.br/posreligiao/publicacoes/teses-e-dissertacoes/teses-de-doutorado-2004-a-2009>

<http://portal.metodista.br/posreligiao/publicacoes/teses-e-dissertacoes/teses-de-doutorado-2010-a-2019>

<http://portal.metodista.br/posreligiao/publicacoes/teses-e-dissertacoes/dissertacoes-de-mestrado-1981-a-2000-1>

<http://portal.metodista.br/posreligiao/publicacoes/teses-e-dissertacoes/dissertacoes-de-mestrado-2001-a-2009-1>

<http://portal.metodista.br/posreligiao/publicacoes/teses-e-dissertacoes/dissertacoes-de-mestrado-2010-a-2019>

35) Universidade Anhembi Morumbi

36) Universidade Nove de Julho

<https://bibliotecatede.uninove.br/simple-search?query=leprosy>

<https://bibliotecatede.uninove.br/simple-search?location=%2F&query=leprosy+and+clarithromycin&rpp=10&sort_by=score&order=desc>

37) Universidade Federal de São Carlos

<https://repositorio.ufscar.br/handle/ufscar/1538/discover?query=leprosy+&submit=>

<https://repositorio.ufscar.br/handle/ufscar/1538/discover?query=leprosy+and+clarithromycin&submit=>

38) Universidade Federal do Acre

<http://www2.ufac.br/ppge/@@busca?SearchableText=leprosy>

<http://www2.ufac.br/ppge/@@busca?SearchableText=leprosy+and+clarithromycin>

39) Universidade Federal de Alagoas

<http://www.repositorio.ufal.br/simple-search?query=leprosy>

<http://www.repositorio.ufal.br/simple-search?location=%2F&query=leprosy+and+clarithromycin&rpp=10&sort_by=score&order=desc>

40) Universidade Federal do Amazonas

<https://tede.ufam.edu.br/simple-search?query=leprosy>

<https://tede.ufam.edu.br/simple-search?location=%2F&query=leprosy+and+clarithromycin&rpp=10&sort_by=score&order=desc>

41) Universidade Salvador

<https://eds.s.ebscohost.com/eds/results?vid=0&sid=30c85ec3-7c3d-4198-8c9f-4f4cb85d6e39%40redis&bquery=leprosy&bdata=JmNsaTA9RlQxJmNsdjA9WSZsYW5nPXB0LWJyJnR5cGU9MCZzZWFyY2hNb2RlPUFuZCZzaXRlPWVkcy1saXZl>

<https://eds.s.ebscohost.com/eds/results?vid=7&sid=30c85ec3-7c3d-4198-8c9f-4f4cb85d6e39%40redis&bquery=leprosy+and+clarithromycin&bdata=JmNsaTA9RlQxJmNsdjA9WSZsYW5nPXB0LWJyJnR5cGU9MCZzZWFyY2hNb2RlPUFuZCZzaXRlPWVkcy1saXZl>

42) Universidade Estadual de Feira de Santana

<http://tede2.uefs.br:8080/simple-search?query=leprosy>

<http://tede2.uefs.br:8080/simple-search?location=%2F&query=leprosy+and+clarithromycin&rpp=10&sort_by=score&order=desc>

43) Universidade de Federal da Bahia

<https://repositorio.ufba.br/ri/simple-search?query=leprosy&submit=Ir>

<https://repositorio.ufba.br/simple-search?location=%2F&query=leprosy+and+clarithromycin&rpp=10&sort_by=score&order=desc>

44) Universidade do Estado da Bahia

<http://www.cdi.uneb.br/site/?cat-trabalhos-academicos=pgdr&termo=filtro-todos&termo-valor=leprosy>

<http://www.cdi.uneb.br/site/?cat-trabalhos-academicos=pgdr&termo=filtro-todos&termo-valor=leprosy+and+clarithromycin>

45) Universidade Federal do Ceará

<http://www.repositorio.ufc.br/simple-search?query=leprosy>

<https://repositorio.ufc.br/simple-search?location=%2F&query=leprosy+and+clarithromycin&rpp=10&sort_by=score&order=desc>

46) Universidade de Brasília

<https://repositorio.unb.br/simple-search?query=leprosy>

<https://repositorio.unb.br/simple-search?location=%2F&query=leprosy+and+clarithromycin&rpp=10&sort_by=score&order=desc>

47) Universidade Católica de Brasília

<https://bdtd.ucb.br:8443/jspui/simple-search?query=leprosy>

<https://bdtd.ucb.br:8443/jspui/simple-search?location=%2F&query=leprosy+and+clarithromycin&rpp=10&sort_by=score&order=desc>

48) Universidade Federal do Espírito Santo

<http://repositorio.ufes.br/simple-search?query=leprosy>

<http://repositorio.ufes.br/simple-search?location=%2F&query=leprosy+and+clarithromycin&rpp=10&sort_by=score&order=desc>

49) Universidade Federal de Goiás

<https://repositorio.bc.ufg.br/tede/simple-search?query=leprosy>

<https://repositorio.bc.ufg.br/tede/simple-search?location=%2F&query=leprosy+and+clarithromycin&rpp=10&sort_by=score&order=desc>

50) Universidade Católica de Goiás

<http://tede2.pucgoias.edu.br:8080/simple-search?query=leprosy>

<http://tede2.pucgoias.edu.br:8080/simple-search?location=%2F&query=leprosy+and+clarithromycin&rpp=10&sort_by=score&order=desc>

51) Universidade Federal Mato Grosso do Sul

<https://repositorio.ufms.br:8443/jspui/handle/123456789/52/browse?type=subject&order=ASC&rpp=20&starts_with=leprosy>

<https://repositorio.ufms.br/handle/123456789/52/browse?type=subject&sort_by=1&order=ASC&rpp=20&etal=-1&value=Leprosy&starts_with=leprosy+and+clarithromycin>

52) Universidade Federal da Grande Dourados

<http://repositorio.ufgd.edu.br/jspui/simple-search?query=leprosy>

<https://repositorio.ufgd.edu.br/jspui/simple-search?location=%2F&query=leprosy+and+clarithromycin&rpp=10&sort_by=score&order=desc>

53) Universidade Católica Dom Bosco

<https://site.ucdb.br/cursos/4/mestrado-e-doutorado/32/mestrado-e-doutorado-em-educacao/13167/doutorado-em-educacao/13192/teses-defendidas/13197/#busca=leprosy>

<https://site.ucdb.br/cursos/4/mestrado-e-doutorado/32/mestrado-e-doutorado-em-educacao/13167/doutorado-em-educacao/13192/teses-defendidas/13197/#busca=leprosy%20and%20clarithromycin>

54) Universidade Federal de Uberlândia

<http://repositorio.ufu.br/simple-search?query=leprosy>

<http://repositorio.ufu.br/simple-search?location=%2F&query=leprosy+and+clarithromycin&rpp=10&sort_by=score&order=desc>

55) Universidade Federal de Juiz de Fora

<http://repositorio.ufjf.br:8080/jspui/simple-search?query=leprosy>

<http://repositorio.ufjf.br:8080/jspui/simple-search?location=%2F&query=leprosy+and+clarithromycin&rpp=10&sort_by=score&order=desc>

56) Universidade Federal de Lavras

<http://repositorio.ufla.br/simple-search?query=leprosy+>

<http://repositorio.ufla.br/simple-search?location=%2F&query=leprosy+and+clarithromycin&rpp=10&sort_by=score&order=desc>

57) Universidade Federal de Minas Gerais

<https://repositorio.ufmg.br/simple-search?query=leprosy>

<https://repositorio.ufmg.br/simple-search?location=%2F&query=leprosy+and+clarithromycin&rpp=10&sort_by=score&order=desc>

58) Universidade Federal de Ouro Preto

<https://www.repositorio.ufop.br/simple-search?location=%2F&query=leprosy+&rpp=10&sort_by=score&order=desc>

<https://www.repositorio.ufop.br/simple-search?query=leprosy+and+clarithromycin>

59) Universidade Federal de Viçosa

<https://www.locus.ufv.br/handle/123456789/1/discover?query=leprosy+&submit=Ir>

<https://www.locus.ufv.br/simple-search?query=leprosy+and+clarithromycin>

60) Pontifícia Universidade Católica de Minas Gerais

<https://web.sistemas.pucminas.br/BDP/PUC%20Minas>

61) Centro Universitário de Caratinga

<https://saas3.bnweb.org/unec/bnportal/pt-BR/search?exp=leprosy%20>

<https://saas3.bnweb.org/unec/bnportal/pt-BR/search?exp=leprosy%20and%20clarithromycin>

62) Universidade Federal do Pará

<http://www.repositorio.ufpa.br/jspui/handle/2011/2289/browse?type=subject&order=ASC&rpp=20&starts_with=leprosy>

<http://www.repositorio.ufpa.br/jspui/handle/2011/2289/browse?type=subject&order=ASC&rpp=20&starts_with=leprosy+and+clarithromycin>

63) Universidade Federal da Paraíba

<https://repositorio.ufpb.br/jspui/simple-search?query=leprosy>

<https://repositorio.ufpb.br/jspui/simple-search?location=%2F&query=leprosy+and+clarithromycin&rpp=10&sort_by=score&order=desc>

64) Universidade Federal do Paraná

<https://acervodigital.ufpr.br/handle/1884/284/discover?query=leprosy+&submit=>

<https://acervodigital.ufpr.br/handle/1884/284/discover?query=leprosy+and+clarithromycin&submit=>

65) Pontifícia Universidade Católica do Paraná

66) Universidade Estadual de Londrina

<http://www.bibliotecadigital.uel.br/document/results.php?words=leprosy>

<http://www.bibliotecadigital.uel.br/document/results.php?words=leprosy+and+clarithromycin>

67) Universidade Estadual do Oeste do Paraná

<http://tede.unioeste.br/simple-search?query=leprosy>

<https://tede.unioeste.br/simple-search?location=%2F&query=leprosy+and+clarithromycin&rpp=10&sort_by=score&order=desc>

68) Universidade Estadual de Ponta Grossa

<https://tede2.uepg.br/jspui/simple-search?location=%2F&query=leprosy+&rpp=10&sort_by=score&order=desc>

<https://tede2.uepg.br/jspui/simple-search?query=leprosy+and+clarithromycin>

69) Universidade Estadual de Maringá

<http://nou-rau.uem.br/nou-rau/document/results.php?words=leprosy>

<http://nou-rau.uem.br/nou-rau/document/results.php?words=leprosy+and+clarithromycin>

70) Universidade Federal de Pernambuco

<https://repositorio.ufpe.br/handle/123456789/50/browse?type=subject&order=ASC&rpp=20&value=Leprosy>

<https://repositorio.ufpe.br/simple-search?query=leprosy+and+clarithromycin>

71) Universidade de Pernambuco

<https://w2.solucaoatrio.net.br/somos/upe-csaude/index.php/pt/doutorado/teses-doutorado>

72) Universidade Católica de Pernambuco

<http://tede2.unicap.br:8080/simple-search?query=leprosy>

<http://tede2.unicap.br:8080/simple-search?location=%2F&query=leprosy+and+clarithromycin&rpp=10&sort_by=score&order=desc>

73) Universidade Federal do Piauí

<https://sigaa.ufpi.br/sigaa/public/biblioteca/buscaPublicaAcervo.jsf>

74) Universidade do Estado do Rio de Janeiro

<https://www.bdtd.uerj.br:8443/simple-search?location=%2F&query=leprosy+&rpp=10&sort_by=score&order=desc>

<https://www.bdtd.uerj.br:8443/simple-search?query=leprosy+and+clarithromycin>

75) Universidade Federal do Estado do Rio de Janeiro

<https://pantheon.ufrj.br/simple-search?query=leprosy>+

<https://pantheon.ufrj.br/simple-search?location=%2F&query=leprosy+and+clarithromycin&rpp=10&sort_by=score&order=desc>

76) Universidade Federal Fluminense

<https://app.uff.br/riuff/discover?scope=%2F&query=leprosy+&submit=>

<https://app.uff.br/riuff/discover?scope=%2F&query=leprosy+and+clarithromycin&submit=&rpp=10>

77) Universidade Federal do Rio de Janeiro

<https://pantheon.ufrj.br/simple-search?location=%2F&query=leprosy&rpp=10&sort_by=score&order=desc>

<https://pantheon.ufrj.br/simple-search?query=leprosy+and+clarithromycin>

78) Universidade Federal Rural do Rio de Janeiro

<https://tede.ufrrj.br/jspui/simple-search?query=leprosy>

<https://tede.ufrrj.br/jspui/simple-search?location=%2F&query=leprosy+and+clarithromycin&rpp=10&sort_by=score&order=desc>

79) Pontifícia Universidade Católica do Rio de Janeiro

<https://www.maxwell.vrac.puc-rio.br/colecao.php?strSearch=leprosy&strTit=&strAut=>

<https://www.maxwell.vrac.puc-rio.br/colecao.php?strSearch=leprosy+and+clarithromycin&strTit=&strAut=>

80) Fundação Oswaldo Cruz

<http://teses.icict.fiocruz.br/cgi-bin/wxis1660.exe/lildbi/iah/>

<https://www.arca.fiocruz.br/simple-search?query=leprosy+and+clarithromycin>

81) Universidade Federal do Rio Grande do Norte

<https://sigaa.ufrn.br/sigaa/public/biblioteca/buscaPublicaAcervo.jsf>

82) Universidade Potiguar

<https://eds.p.ebscohost.com/eds/results?vid=3&sid=6586bbe7-fdf6-49c3-96b8-b260c6f5d992%40redis&bquery=leprosy&bdata=JmNsaTA9RlQxJmNsdjA9WSZsYW5nPXB0LWJyJnR5cGU9MCZzZWFyY2hNb2RlPUFuZCZzaXRlPWVkcy1saXZl>

<https://eds.p.ebscohost.com/eds/results?vid=9&sid=6586bbe7-fdf6-49c3-96b8-b260c6f5d992%40redis&bquery=leprosy+and+clarithromycin&bdata=JmNsaTA9RlQxJmNsdjA9WSZsYW5nPXB0LWJyJnR5cGU9MCZzZWFyY2hNb2RlPUFuZCZzaXRlPWVkcy1saXZl>

83) Universidade Federal do Rio Grande do Sul

<https://sabi.ufrgs.br/F/XHY991GN9UH98297D9UI6VX1YUXJ746348FNIN2DMY1XU18HKE-17446?func=find-b&request=leprosy&find_code=WRD&adjacent=N&x=49&y=5&filter_code_2=WLN&filter_request_2=&filter_code_3=WYR&filter_request_3=&filter_code_4=WYR&filter_request_4=>

<https://sabi.ufrgs.br/F/LELR5FVGUF3HAXPSYYNPQRJHV23KCB7FXTA7G94TIRM7TPSALB-10955?func=find-b&request=leprosy+and+clarithromycin&find_code=WRD&adjacent=N&x=33&y=7&filter_code_2=WLN&filter_request_2=&filter_code_3=WYR&filter_request_3=&filter_code_4=WYR&filter_request_4=>

84) Universidade Federal de Santa Maria

<https://repositorio.ufsm.br/discover?scope=%2F&query=leprosy+&submit=>

<https://repositorio.ufsm.br/discover?scope=%2F&query=leprosy+and+clarithromycin&submit=>

85) Fundação Universidade Federal do Rio Grande

https://lume.ufrgs.br/handle/10183/1/discover?query=leprosy+and+clarithromycin&submit=&dateMode=select&querytype_0=title&query_relational_operator_0=contains&query_value_0=&querytype_1=authortd&query_relational_operator_1=contains&query_value_1=&querytype_2=orientador&query_relational_operator_2=contains&query_value_2=&querytype_3=subject&query_relational_operator_3=contains&query_value_3=&querytype_4=dateIssued&query_relational_operator_4=contains&query_value_4=&select_mes_inicio_4=&select_dia_inicio_4=&select_mes_fim_4=&select_dia_fim_4=&querytype_5=dataAno&query_relational_operator_5=equals&query_value_5=&querytype_6=nivelAcademico&query_relational_operator_6=equals&query_value_6=&querytype_7=tipo&query_relational_operator_7=equals&query_value_7=&querytype_8=idioma&query_relational_operator_8=equals&query_value_8=&querytype_9=formatoArquivo&query_relational_operator_9=equals&query_value_9=&querytype_10=serie&query_relational_operator_10=contains&query_value_10=&querytype_11=author&query_relational_operator_11=contains&query_value_11=&querytype_12=acervo&query_relational_operator_12=contains&query_value_12=&querytype_13=descriptionSection&query_relational_operator_13=contains&query_value_13=&querytype_14=tipoAto&query_relational_operator_14=contains&query_value_14=&querytype_15=natureza&query_relational_operator_15=contains&query_value_15=&querytype_16=numeroAto&query_relational_operator_16=contains&query_value_16=&querytype_17=orgao&query_relational_operator_17=contains&query_value_17=&querytype_18=dataFinal&query_relational_operator_18=contains&query_value_18=&select_mes_inicio_18=&select_dia_inicio_18=&select_mes_fim_18=&select_dia_fim_18=&querytype_19=programa&query_relational_operator_19=contains&query_value_19=&querytype_20=entrevistado&query_relational_operator_20=contains&query_value_20=&querytype_21=grandeArea&query_relational_operator_21=contains&query_value_21=&querytype_22=tipoDeApresentacao&query_relational_operator_22=contains&query_value_22=&querytype_23=areaTematica&query_relational_operator_23=contains&query_value_23=&querytype_24=coordenador&query_relational_operator_24=contains&query_value_24=&querytype_25=origem&query_relational_operator_25=contains&query_value_25=&querytype_26=unidade&query_relational_operator_26=contains&query_value_26=&querytype_27=status&query_relational_operator_27=contains&query_value_27=&querytype_28=curso&query_relational_operator_28=contains&query_value_28=&querytype_29=nivelDeEnsino&query_relational_operator_29=contains&query_value_29=&querytype_30=tipoDeMaterial&query_relational_operator_30=contains&query_value_30=

86) Universidade Católica de Pelotas

<https://pos.ucpel.edu.br/ppgsc/?s=leprosy>

<https://pos.ucpel.edu.br/ppgsc/?s=leprosy+and+clarithromycin>

87) Pontifícia Universidade Católica do Rio Grande do Sul

<http://tede2.pucrs.br/tede2/simple-search?query=leprosy>

<http://tede2.pucrs.br/tede2/simple-search?location=%2F&query=leprosy+and+clarithromycin&rpp=10&sort_by=score&order=desc>

88) Universidade de Passo Fundo

<https://secure.upf.br/pergamum/biblioteca/index.php>

89) Universidade de Caxias do Sul

<https://repositorio.ucs.br/xmlui/handle/11338/37/discover?query=leprosy+&submit=>

<https://repositorio.ucs.br/xmlui/handle/11338/37/discover?query=leprosy+and+clarithromycin&submit=>

90) Universidade do Vale do Rio dos Sinos

<http://www.repositorio.jesuita.org.br/handle/UNISINOS/1565/discover?query=leprosy+&submit=>+++

<http://www.repositorio.jesuita.org.br/handle/UNISINOS/1565/discover?query=leprosy+and+clarithromycin&submit=>+++

91) Universidade Luterana do Brasil

<https://ri.unir.br/jspui/simple-search?location=%2F&query=leprosy+&rpp=10&sort_by=score&order=desc&filter_field_1=subject&filter_type_1=equals&filter_value_1=Hansen%C3%ADase>

92) Universidade Federal de Rondônia

<https://ri.unir.br/jspui/simple-search?location=%2F&query=hansen%C3%ADase+e+claritromicina&rpp=10&sort_by=score&order=desc&filter_field_1=subject&filter_type_1=equals&filter_value_1=Hansen%C3%ADase>

<https://ri.unir.br/jspui/simple-search?location=%2F&query=leprosy+and+clarithromycin&rpp=10&sort_by=score&order=desc&filter_field_1=subject&filter_type_1=equals&filter_value_1=Hansen%C3%ADase>

93) Universidade Federal de Santa Catarina

<https://repositorio.ufsc.br/handle/123456789/74645/discover?filtertype_1=title&filter_relational_operator_1=contains&filter_1=leprosy&submit_apply_filter=Aplicar&query=leprosy>+

<https://repositorio.ufsc.br/handle/123456789/74645/discover?filtertype_1=title&filter_relational_operator_1=contains&filter_1=leprosy&filtertype_2=title&filter_relational_operator_2=contains&filter_2=clarithromycin&submit_apply_filter=Aplicar&query=leprosy>+

94) Universidade do Estado de Santa Catarina

<https://pergamumweb.udesc.br/biblioteca/index.php>

95) Universidade do Vale do Itajaí

<https://eds.s.ebscohost.com/eds/results?vid=3&sid=1376ae56-49e2-4828-9396-60b6e23b570f%40redis&bquery=leprosy&bdata=JmNsaTA9RlQxJmNsdjA9WSZsYW5nPXB0LWJyJnR5cGU9MCZzZWFyY2hNb2RlPUFuZCZzaXRlPWVkcy1saXZlJnNjb3BlPXNpdGU%3d>

<https://eds.s.ebscohost.com/eds/results?vid=5&sid=1376ae56-49e2-4828-9396-60b6e23b570f%40redis&bquery=leprosy+and+clarithromycin&bdata=JmNsaTA9RlQxJmNsdjA9WSZsYW5nPXB0LWJyJnR5cGU9MCZzZWFyY2hNb2RlPUFuZCZzaXRlPWVkcy1saXZlJnNjb3BlPXNpdGU%3d>

96) Universidade Regional de Blumenau

<https://bu.furb.br/consulta/novaConsulta/pesqPosGrad.php?acao=pesquisar&rdbtTpConsulta=10&ExpBusca=Leprosy&anoDefesaIni=--Todos+os+anos--&anoDefesaFim=--Todos+os+anos--&progPG=--Todos+os+programas--&Submit=Pesquisar>

<https://bu.furb.br/consulta/novaConsulta/pesqPosGrad.php?acao=pesquisar&rdbtTpConsulta=10&ExpBusca=leprosy+and+clarithromycin&anoDefesaIni=--Todos+os+anos--&anoDefesaFim=--Todos+os+anos--&progPG=--Todos+os+programas--&Submit=Pesquisar>

97) Universidade Federal de Sergipe

<https://ri.ufs.br/handle/riufs/2145/browse?type=subject&order=ASC&rpp=20&value=Leprosy>

<https://ri.ufs.br/handle/riufs/2145/browse?type=subject&sort_by=1&order=ASC&rpp=20&etal=-1&value=Leprosy&starts_with=leprosy+and+clarithromycin>

98) Universidad Nacional de Colombia

<https://eds.p.ebscohost.com/eds/detail/detail?vid=0&sid=5a729644-b708-4e70-800d-9649e45faabd%40redis&bdata=Jmxhbmc9cHQtYnImc2l0ZT1lZHMtbGl2ZQ%3d%3d#AN=edsbas.DCD6E4D9&db=edsbas>

**Figure 1S-A.** Flow chart of studies selection of clofazimine review.

**Clofazimine review**

**
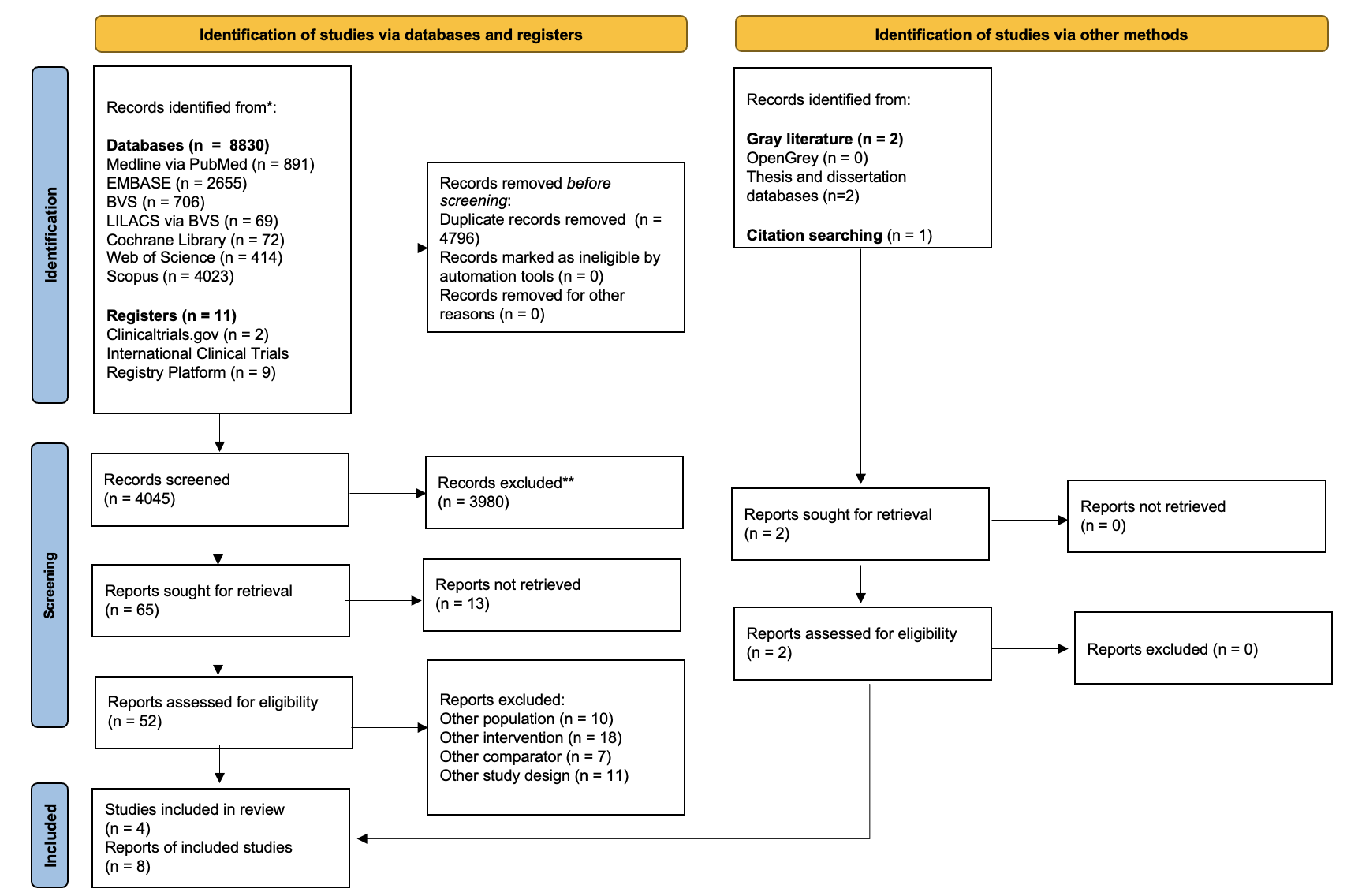
**

**Figure 2S-A.** Flow chart of studies selection of clarithromycin review.

Clarithromycin review

**
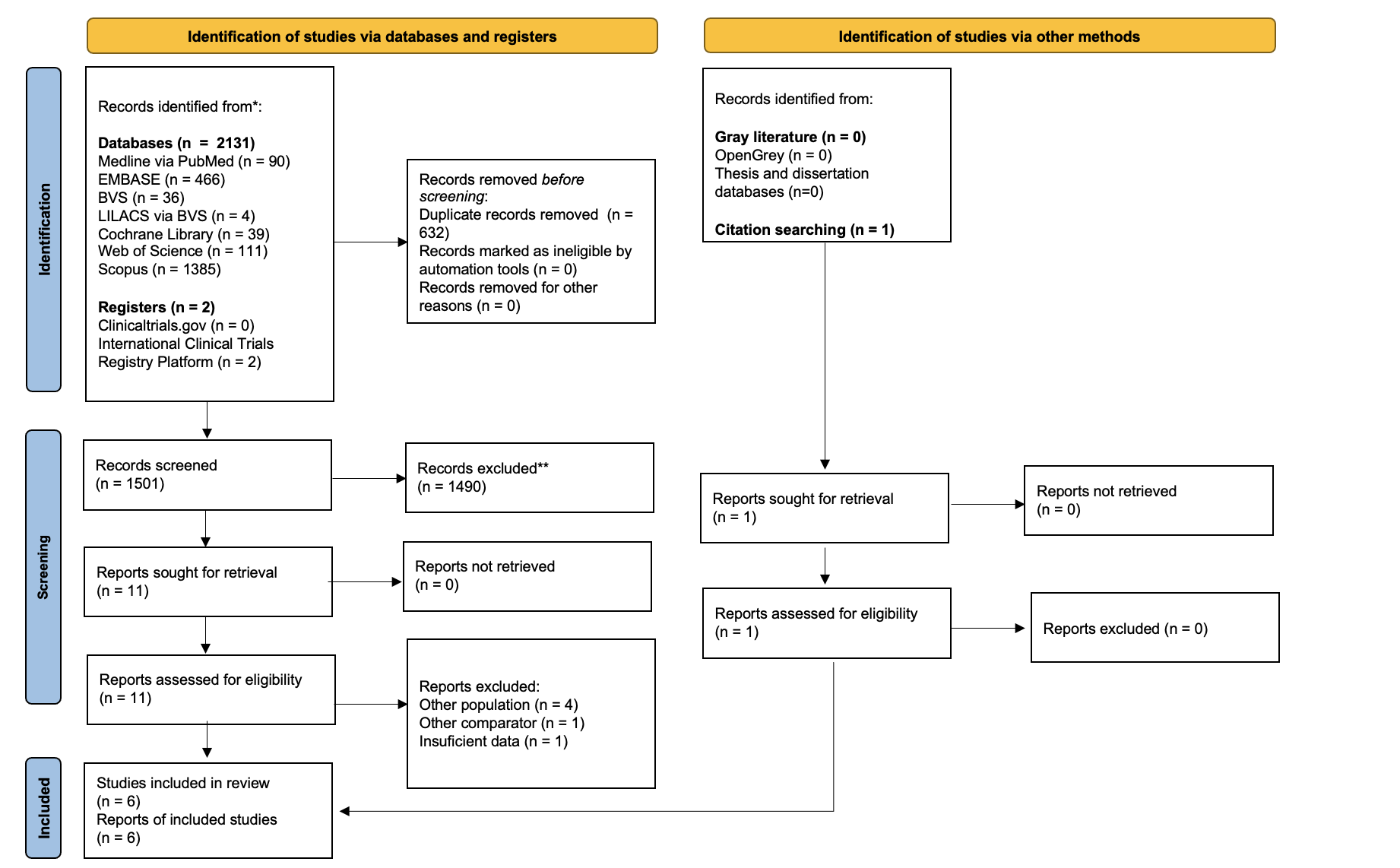
**

**Table 3S-A.** Excluded studies in the selection of reports assessed for eligibility and the reasons for exclusion for Clofazimine review.

| **Author (year)** | **Publication Title** | **Reason for exclusion** | |  |
| --- | --- | --- | --- | --- |
| - | A second report on multidrug therapy for leprosy in Trinidad and Tobago | Other population | |  |
| Browne (1965) | Treatment of leprosy with B 663 appraisal of the pilot trial after three years | Other population | |  |
| Chatterjee (1989) | Bacillaemia in leprosy and effect of multidrug therapy | Other population | |  |
|  |  |  |  |  |
| Goto (1998) | Chemotherapy of Hansen's disease in Japan--present status | Other population |  |  |
| Katoch (1987) | Follow up of BL/LL patients on a slightly modified WHO regimen of multidrug therapy | Other population |  |  |
| Pieters (1988) | Influence of once-monthly rifampicin and daily clofazimine on the pharmacokinetics of dapsone in leprosy patients in Nigeria | Other population |  |  |
| Ramasoota (1973) | Clinical trial of low doses of B 663 in the treatment of leprosy | Other population |  |  |
| Sapkota (2008) | A retrospective study of the effect of modified multi-drug therapy in Nepali leprosy patients following the development of adverse effects due to dapsone | Other population |  |  |
| Tolentino (1971) | Controlled drug trial of B.663 compared with DDS. Preliminary (48 week) report | Other population |  |  |
| Tolentino (1973) | Controlled long term therapy of leprosy with B 663 | Other population |  |  |
|  |  |  |  |  |
| Andrade (1987) | Feasibility of multidrug therapy (MDT) in Hansen’s disease in an urban population – Curupaiti State Hospital, Rio de Janeiro, Brazil | Other intervention |  |  |
|  |  |  |  |  |
| Bernades (2002) | Adverse effects of multidrug therapy in leprosy patients: A five-year survey at a Health Center of the Federal University of Uberlândia | Other intervention |  |  |
| Bopp (1972) | Clofazimine (Lamprene; G663) in the treatment of leprosy | Other intervention |  |  |
|  |  |  |  |  |
|  |  |  |  |  |
|  |  |  |  |  |
| Chow (2019) | Characterization and Outcomes of Patients with Hansen Disease Treated at the Los Angeles County Hospital | Other intervention |  |  |
| Chum (1986) | The impact of MDT implementation in the Tanzania National TB-Leprosy Programme | Other intervention |  |  |
| De Carsalade (1997) | Daily Multidrug Therapy for Leprosy; Results of a Fourteen-Year Experience | Other intervention |  |  |
| Gallo (1995) | Intercorrências pelas drogas utilizadas nos esquemas poliquimioterápicos em hanseníase | Other intervention |  |  |
|  |  |  |  |  |
| Gatti (1970) | Treatment of leprosy with a phenazine derivative (B 663 or G 30 320)--clofazimine. | Other intervention |  |  |
| Grugni (1990) | Relapses in paucibacillary leprosy. After MDT - A clinical study | Other intervention |  |  |
|  |  |  |  |  |
|  |  |  |  |  |
| John (2004) | Incidence of neuritis among paucibacillary leprosy patients during treatment and surveillance | Other intervention |  |  |
| Kumar (1987) | Short term combination therapy for paucibacillary leprosy--histological evaluation & follow-up study. | Other intervention |  |  |
| Lim (1993) | Efficacy and safety of multidrug therapy in paucibacillary leprosy in Singapore. | Other intervention |  |  |
|  |  |  |  |  |
| Mehta (1985) | Effect of clofazimine and dapsone on rifampicin (lositril) pharmacokinetics in multibacillary and paucibacillary leprosy cases | Other intervention |  |  |
|  |  |  |  |  |
| Pattyn (1994) | A randomized clinical trial of two single-dose treatments for paucibacillary leprosy | Other intervention |  |  |
| Rose (1984) | Short-course multi-drug therapy for paucibacillary patients in Guyana: Preliminary communication | Other intervention |  |  |
| Samuel (1984) | Multidrug treatment of leprosy - practical application in Nepal | Other intervention |  |  |
|  |  |  |  |  |
|  |  |  |  |  |
| Tiwari (1988) | Multidrug therapy in hospitalised leprosy cases | Other intervention |  |  |
|  |  |  |  |  |
|  |  |  |  |  |
| Vara (2005) | A study on the impact of FD-MDT on 200 leprosy patients | Other intervention |  |  |
| Belda (1972) | Clinical trial of clofazimine in the outpatient therapy of leprosy | Other comparator |  |  |
| Biswas (2003) | Multidrug therapy in leprosy can prevent relapse - A retrospective study | Other comparator |  |  |
|  |  |  |  |  |
|  |  |  |  |  |
| Browne (1961) | ''B 663'' in the treatment of leprosy supplementary report of the pilot trial | Other comparator |  |  |
|  |  |  |  |  |
|  |  |  |  |  |
| Hastings (1976) | Long term clinical toxicity studies with clofazimine (B663) in leprosy | Other comparator |  |  |
|  |  |  |  |  |
| Karuru (1970) | Clinical evaluation of Lamprene (Geigy). A preliminary report. | Other comparator |  |  |
|  |  |  |  |  |
|  |  |  |  |  |
| Opromolla (1973) | Use of clofazimine (B 663) in the treatment of leprosy | Other comparator |  |  |
|  |  |  |  |  |
| Ramakrishnan (1988) | Paucibacillary leprosy: A comparative study of different schedules of multidrug therapy | Other comparator |  |  |
| Blok (1991) | A retrospective study on seven years of multiple drug treatment for paucibacillary and multibacillary leprosy, in Bayara General Hospital, Nigeria | Other study design |  |  |
| Browne (1972) | Clofazimine in Leprosy | Other study design |  |  |
|  |  |  |  |  |
| Carvalho (1973) | Chemotherapeutic trial with clofazimine (1) in leprosy | Other study design |  |  |
|  |  |  |  |  |
|  |  |  |  |  |
|  |  |  |  |  |
| Castro-Coto (1977) | Clofazimine in combatting leprosy | Other study design |  |  |
|  |  |  |  |  |
| Mehta (1986) | Effect of clofazimine and dapsone on rifampicin (Lositril) pharmacokinetics in multibacillary and paucibacillary leprosy cases | Other study design |  |  |
| Murray (2003) | Short report: Treatment failure in Hansen's disease | Other study design |  |  |
|  |  |  |  |  |
|  |  |  |  |  |
| NCT00669643 (2008) | Uniform Multidrug Therapy Regimen for Leprosy Patients | Other study design |  |  |
|  |  |  |  |  |
|  |  |  |  |  |
| Pai (1999) | Single-dose treatment for paucibacillary leprosy; clinical problems and management [3] | Other study design |  |  |
| - | Uniform Multidrug Therapy Regimen for Leprosy Patients | Other study design |  |  |
| Theophilus (1973) | Report on treatment with B 663 (Lamprene) on 107 leprosy patients (93 lepromatous and 14 non lepromatous) | Other study design |  |  |
|  |  |  |  |  |
| Williams (1965) | Leprosy research at the National Institutes of Health: experience with B.663 in the treatment of leprosy. | Other study design |  |  |
|  |  |  |  |  |
| Becx-Bleumink (1994) | Multidrug therapy in the control of leprosy. A field study | Other study design |  |  |
|  |  |  |  |  |
|  |  |  |  |  |
| Belda (1973) | Clinical results of treatment of leprosy with clofazimine | Report not retrieved |  |  |
|  |  |  |  |  |
|  |  |  |  |  |
|  |  |  |  |  |
| Bourges (1981) | Summary of anti-leprosy treatments in France and in non-European countries (Francophone areas) | Report not retrieved |  |  |
|  |  |  |  |  |
| Carvalho (1974) | Trial of chemotherapy with clofazimine in leprosy | Report not retrieved |  |  |
|  |  |  |  |  |
|  |  |  |  |  |
|  |  |  |  |  |
| Cnudde (1987) | [Short-term clinical and histological evaluation of combination drug therapy of leprosy in Mayotte]. | Report not retrieved |  |  |
| Desfontaine (1988) | Leprosy polychemotherapy in the Central African Republic in 1987 | Report not retrieved |  |  |
|  |  |  |  |  |
|  |  |  |  |  |
| Devadason (1969) | B.663 (Geigy 30. 320) in the treatment of leprosy. A preliminary report. | Report not retrieved |  |  |
| Dhir (1986) | Short term chemotherapy of paucibacillary leprosy | Report not retrieved |  |  |
|  |  |  |  |  |
| Kundu (1976) | Experiences with clofazimine therapy in leprosy | Report not retrieved |  |  |
|  |  |  |  |  |
|  |  |  |  |  |
| Millan (1986) | [A trial of polychemotherapy of leprosy in the Dakar region. Initial observations on the acceptability of the protocols used]. | Report not retrieved |  |  |
| N'Deli (1986) | Practical problems of polychemotherapy of leprosy in Ivory Coast | Report not retrieved |  |  |
| Ramu (1976) | Side effects of clofazimine therapy. | Report not retrieved |  |  |
|  |  |  |  |  |
| Ribeiro (1997) | Leprosy polichemotherapy and new therapeutics in Araraquara, Sao Paulo - 1990-1993 | Report not retrieved |  |  |

**Table 4S-A.** Excluded studies in the selection of reports assessed for eligibility and the reasons for exclusion for Clarithromycin review.

| **Author (year)** | **Publication Title** | **Reason for exclusion** |
| --- | --- | --- |
| Astari et al., 2021 | A 5-year evaluation of chemoprophylactic treatment in elementary school children with subclinical leprosy | Other population |
| Chan et al., 1994 | Clinical-trial of clarithromycin for lepromatous leprosy | Other population |
| Kroger et al., 2008 | International open trial of uniform multi-drug therapy regimen for 6 months for all types of leprosy patients: Rationale, design and preliminary results | Other population |
| Kumar et al., 2015 | WHO multidrug therapy for leprosy: Epidemiology of default in treatment in Agra District, Uttar Pradesh, India | Other population |
| Rea, 2000 | Trials of Daily, Long-Term Minocycline and Rifampin or Clarithromycin and Rifampin in the Treatment of Borderline Lepromatous and Lepromatous Leprosy | Insufficient data |
| Rea, 2001 | Decreases in mean hemoglobin and serum albumin values in erythema nodosum leprosum and lepromatous leprosy | Other comparator |
