# Supplementary material for: Effectiveness and safety of multidrug therapy containing clofazimine for paucibacillary leprosy and clarithromycin for rifampicin-resistant leprosy: a systematic review and meta-analysis
Source: Front Med (Lausanne). 2023 May 10;10:1139304. doi: 10.3389/fmed.2023.1139304 (PMC10206035; doi:10.3389/fmed.2023.1139304)
Supplement: Supplementary material B — Detailed evaluation of all secondary outcomes included in the clofazimine review, clarithromycin review, and certainty of evidence analysis using the GRADE approach. [file Data_Sheet_2.docx]

SUPPLEMENTARY FILE B

**Detailed evaluation of all secondary outcomes included in clofazimine review and clarithromycin review and certainty of evidence analysis using GRADE approach.**

Table 1S-B discloses, in detail, all data extracted and evaluated in this review.

**Table 1S-B**. A list of data extracted from the studies included in the present review.

| Type of information | Information and variables extracted |
| --- | --- |
| Bibliographic data | Journal publication name, author names, year of publication |
| Study characteristics | Study site, number of centres, sample size, number of subjects in each treatment arm, study inclusion and exclusion criteria, study design, follow-up time |
| Sample characteristics | Age, sex, diagnostic method, diagnosis time, disease classification |
| Intervention characteristics | Administration method, dosage, administration frequency and treatment duration, use of adjuvant therapies |
| Outcome measurement method and outcome data at all follow-up times | Number of individuals evaluated in each follow-up of outcomes analysed in each intervention arm. The following continuous outcomes were extracted: measures of central tendency and variability, mean preference and standard deviation (bacteriological and morphological index, quality of life). The following dichotomous outcomes were extracted: number of cures, number of relapses, number of any adverse events, number of serious adverse events and percentage of drug-adhering individuals. |

**CLOFAZIMINE-RELATED OUTCOMES**

**Risk of bias**

The RoB of the included RCTs (19,20,22) was assessed using the RoB 2 tool for the primary outcomes of this systematic review. The studies by Bhathe et al.(19) and Katoch et al. (20) were classified as having a high RoB for the cure outcome, as shown in Figure 1S-B.

**Figure 1S-B.** Risk of bias assessment of the included RCTs for the cure outcome, performed using the RoB 2 tool.

The study by Katoch et al.,(20) was classified as having a high RoB for the relapse outcome, as shown in Figure 2S-B.

**Figure 2S-B.** Assessment of the risk of bias of the included RCT for the relapse outcome, performed using the RoB 2 tool.

The studies published by Bhate et al.,(19) Gonçalves et al.,(22) and Katoch et al., (20) presented a high RoB for the outcome ‘adverse events’, as shown in Figure 3S-B.

**Figure 3S-B.** Risk of bias assessment of the included RCTs for the adverse event outcome, performed using the RoB 2 tool.

The nonrandomized clinical trial study (23) was evaluated by the ROBINS-I tool for the outcomes ‘cure’ and ‘adverse events’, and the analysis of its domains can be seen in Figure 4S-B.


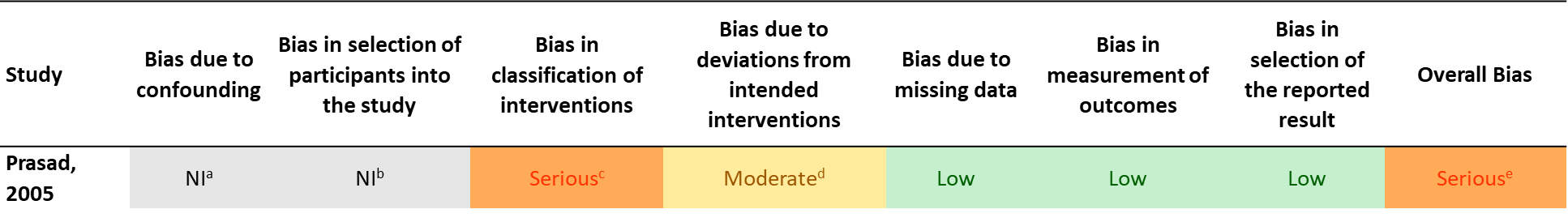


**Figure 4S-B.** Risk of bias of the included nonrandomized clinical trial, assessed using the ROBINS-I tool.

^a^ The study did not provide information on the presence of confounders and on appropriate analyses to control them.

^b^ The study did not provide information on the method of selection of participants.

^c^ The study did not specify how the interventions were administered or their dosages.

^d^ There were deviations from usual practice, but it was expected that there would be no influence on the outcome because these were objective (Cure and Adverse Events).

^e^ The study had some major issues, and at least one domain was judged as a serious risk but none as a critical risk.

**SECONDARY OUTCOMES (CLOFAZIMINE REVIEW)**

**Safety**

*Six-month follow-up*

Total adverse events (AEs) were reported in four studies at a six-month follow-up time;(20,22,23) however, it was possible to perform a meta-analysis of only those events data from three of the studies.(19,20,23)

Bhate et al.,(19) observed the presence of AEs in six patients in the clofazimine group (receiving clofazimine, rifampicin and dapsone, with four patients developing neuritis and two experiencing reactions in the lesions during multidrug therapy) and in four patients in the control group (receiving rifampicin and dapsone), of a total of 40 patients evaluated in each group. The study by Katoch et al.,(20) observed the presence of four patients with erythema, infiltrations, and lesions after the treatment period in the clofazimine group and three new lesions in this group, whereas in the control group, it was observed that six patients had the same AE and four new injuries. The study by Prasad et al.,(23) observed one patient with a reverse reaction after six months of completion in the intervention group and no patients with AEs in the control group.

As shown in Figure 5S-B, for the outcome ‘total adverse events’, a summary estimate of RR 1.07 (95% CI 0.47 to 2.45) was found. It is also possible to observe that there was no significant statistical heterogeneity among the studies included in the meta-analysis (Chi^2^ with *p value* = 0.49; I^2^ of 0%).


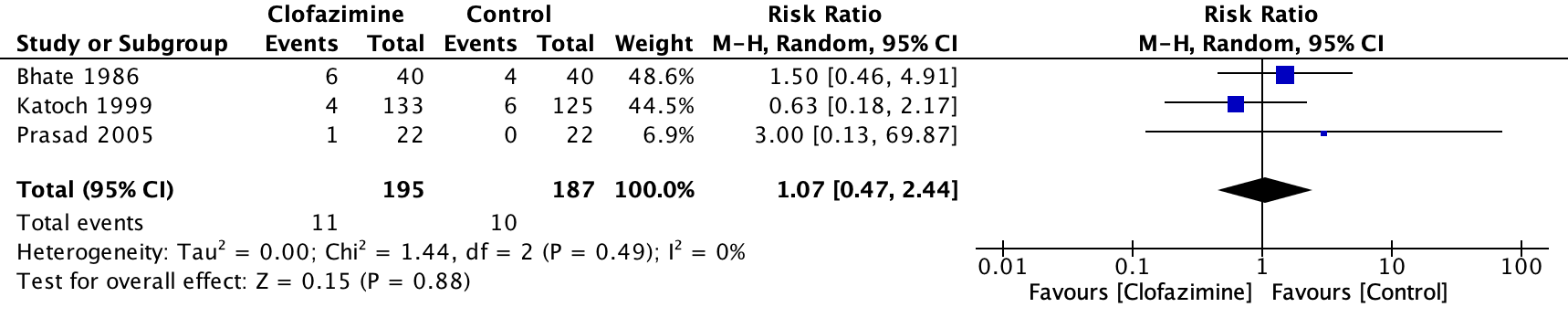


**Figure 5S-B**. Forest plot of the meta-analysis of the outcome ‘total adverse events’ at the six-month follow-up time

The study by Gonçalves et al.,(22) showed that the clofazimine group had a 95% incidence of haemolytic anaemia, with a 65% incidence in the control group. The most frequent AEs were red blood cell reduction (65%: control group; 95%: clofazimine group), reduced haematocrit (65%: control group; 95%: clofazimine group), and decreased haemoglobin (60%: control group; 90%: clofazimine group). The most common symptoms were headache (15%: control group; 10%: clofazimine group), fatigue (15%: control group; 10%: clofazimine group), and dizziness (10%: control group; 0%: clofazimine group).

*Greater than six-month follow-up*

Bhate et al.,(19) observed that after discontinuation of multidrug therapy, two patients had a reaction with synovial oedema on the dorsum of the wrist, three patients developed reactions in the lesions, and one patient developed neuritis in the clofazimine group (clofazimine, dapsone and rifampicin). In the control group (dapsone and rifampicin), only one case of lesion reaction was observed during the same follow-up period.

Katoch et al.,(20) verified the presence of a late AE in the intervention group with signs of erythema and facial paralysis, which were resolved with steroid treatment. In the control group, two late AEs were observed, one with an erythematous lesion on the face and the other with two lesions (forearm and forehead) that resolved with steroid treatment.

*Quality of life*

The quality-of-life outcome was not evaluated in any of the studies included in the present systematic review.

*Treatment adherence*

The study by Gonçalves et al.,(22) was combined with a publication referring to the same randomized clinical trial (25) that evaluated the outcome ‘adherence to medication use’. In the intervention group (clofazimine, dapsone and rifampicin), 36 participants out of a total of 41 (87.8%) responded that they had not considered interrupting treatment, and in the control group (dapsone and rifampicin), there were 30 participants out of a total of 33 (90.9%).

In addition, the study evaluated the participants' satisfaction with the treatment through a score from zero to five, where zero denoted dissatisfaction with the treatment and five indicated total satisfaction; 38 participants out of 41 evaluated (92.7%) assigned a score from three to five for satisfaction, while in the control group, 33 participants (100%) attributed the same score.

*Certainty of evidence analysis using GRADE approach*

The certainty in the final set of evidence was evaluated for the primary outcomes and was classified as very low certainty, as seen in detail in Table 2S-B with the appropriate judgements for the downgrading of the certainty of the evidence for each domain evaluated by the GRADE system approach. It was not possible to assess publication bias due to the low number of studies included in the evaluation of outcomes. In addition, for the recurrence outcome, it was not possible to assess the inconsistency because only one study was involved.

**Table 2S-B.** Summary table of the findings of Clofazimine Review using the GRADE approach

| **Certainty assessment** | | | | | | | **№ of patients** | | **Effect** | | **Certainty** | **Importance** |
| --- | --- | --- | --- | --- | --- | --- | --- | --- | --- | --- | --- | --- |
| **№ of studies** | **Study design** | **Risk of bias** | **Inconsistency** | **Indirectness** | **Imprecision** | **Other considerations** | **Clofazimine, dapsone and rifampicin** | **dapsone and rifampicin** | **Relative (95% CI)** | **Absolute (95% CI)** |  |  |
| Cure (follow-up: 6 months) | | | | | | | | | | | | |
| 3 | Randomized trials and nonrandomized trial | Very serious^a^ | Not serious | Not serious | Very serious^b^ | None | 155/195 (79.5%) | 137/187 (73.3%) | **RR 1.09** (0.92 to 1.29) | **66 more per 1.000** (from 59 fewer to 212 more) | ⨁◯◯◯ Very low | CRITICAL |
| Cure (follow-up: 12 months) | | | | | | | | | | | | |
| 2 | Randomized trials and nonrandomized trial | Very serious^c^ | Very serious^d^ | Not serious | Very serious^b^ | None | 55/62 (88.7%) | 54/62 (87.1%) | **RR 1.05** (0.78 to 1.40) | **44 more per 1.000** (from 192 fewer to 348 more) | ⨁◯◯◯ Very low | CRITICAL |
| Relapse | | | | | | | | | | | | |
| 1 | Randomized trials | serious^e^ | Not serious | Not serious | Very serious^f^ | None | 0/150 (0.0%) | 2/150 (1.3%) | **RR 0.20** (0.01 para 4.13) | **11 more per 1.000** (from 13 fewer to 42 more) | ⨁◯◯◯ Very low | CRITICAL |
| Adverse events (follow-up: 6 months) | | | | | | | | | | | | |
| 3 | Randomized trials and nonrandomized trial | Very serious^g^ | Not serious | Not serious | Very serious^h^ | None | 11/195 (5.6%) | 10/187 (5.3%) | **RR 1.07** (0.47 to 2.44) | **4 more per 1.000** (from 28 fewer to 77 more) | ⨁◯◯◯ Very low | CRITICAL |

**CI:** Confidence interval; **RR:** Risk ratio

**Legends:**

^a^ The RCTs presented a high risk of general bias in the analysis of the cure outcome using the RoB 2 tool, and Bhate et al.,(19) presented some concerns in the randomization process and the deviation of the intended interventions and high risk in the outcome measurement domain, whereas the study by Katoch et al.,(20) showed high risk in the domains of deviation from the intended interventions and missing outcome data. The nonrandomized clinical trial study analysed by the ROBINS-I tool was judged as having a serious risk of bias, as it presented a serious risk of bias in the classification of interventions and a moderate risk of bias in relation to deviations from intended interventions. Therefore, the methodological limitations of the studies would be sufficient to substantially decrease the confidence in the effect estimate.

^b^ The CI for the cure outcome presented as the RR comprised both the null effect and considerable risk or benefit (risk ratio ≥ 1.25 or ≤ 0.75); therefore, it was considered imprecise. In addition, the sample size and the number of events were not sufficient to reach the threshold of optimal information size (OIS).

^c^ The study by Bhate et al.,(19) which was of the RCT type, presented a high risk of general bias in the analysis of the cure outcome using the RoB 2 tool, with some concerns in the randomization process and the deviation of the intended interventions and high risk in the outcome measurement domain. The nonrandomized clinical trial study analysed by the ROBINS-I tool was judged as having a serious risk of bias, as it presented a serious risk of bias in the classification of interventions and a moderate risk of bias in relation to deviations from intended interventions. Therefore, the methodological limitations of the studies would be sufficient to substantially decrease the confidence in the effect estimate.

^d^ In the evaluation of the forest plot graph, it was possible to observe little overlap of the confidence intervals between the two studies; in addition, important statistical heterogeneity was observed (Chi^2^ with *p value* = 0.04; I2 of 75%).

^e^ The study by Katoch et al.,(20) presented high risk in the domains of deviation from intended interventions and missing outcome data through the analysis performed by the RoB 2 tool for the relapse outcome.

^f^ The confidence interval for the outcome ‘relapse’ (RR 0.20; 95% CI 0.01-4.13) comprised both a null effect and a considerable risk or benefit (relative risk ≥ 1.25 or ≤ 0.75), so this was considered inaccurate. In addition, the sample size and the number of events were not sufficient to reach the threshold of optimal information size (OIS).

^g^ The RCT showed a high risk of general bias in the analysis of the outcome ‘total adverse events’ by the RoB 2 tool, and Bhate et al.,(19) presented some concerns in the randomization process and in the deviation of the intended interventions and high risk in the measurement of outcomes domain, whereas the study by Katoch et al.,(20) showed high risk in the domains of deviation from intended interventions and missing outcome data. The nonrandomized clinical trial study analysed by the ROBINS-I tool was judged as having a serious risk of bias, as it presented a serious risk of bias in the classification of interventions and a moderate risk of bias in relation to deviations from intended interventions. Therefore, the methodological limitations of the studies would be sufficient to substantially decrease the confidence in the effect estimate.

^h^ The confidence interval for the outcome ‘total adverse events’ (RR 1.07; 95% CI: 0.47-2.44) comprised both a null effect and a considerable risk or benefit (risk ratio ≥ 1.25 or ≤ 0.75); therefore, this was considered inaccurate). In addition, the sample size and the number of events were not sufficient to reach the threshold of optimal information size (OIS).

**CLARITHROMYCIN-RELATED OUTCOMES**

**Risk of bias**

The risk of bias of RCTs for the cure outcome was assessed using the RoB 2 tool. The study by Girdhar et al.,(27) was classified as having a high risk of general bias, and the studies Ji et al.,(29,30) were assessed as having some concerns (Figure 6S-B).

**Figure 6S-B.** Risk of bias assessment of the RCTs included for the cure outcome, performed using the RoB 2 tool.

The study by Girdhar et al.,(27) was classified as having a high RoB for the recurrence outcome, as shown in Figure 7S-B.

**Figure 7S-B.** Assessment of the risk of bias of the RCT included for the relapse outcome, performed using the RoB 2 tool.

The RoB in the studies by Gunawan et al.,(28) and Tejasvi et al.,(31) was judged as high risk of general bias, and the studies by Ji et al.,(29,30) and Wongdjaja et al.,(32) were judged as presenting some concern regarding the reduction in the bacteriological index and the reduction in the morphological index outcomes, according to the analysis performed using the RoB 2 tool (Figure 8S-B).

**
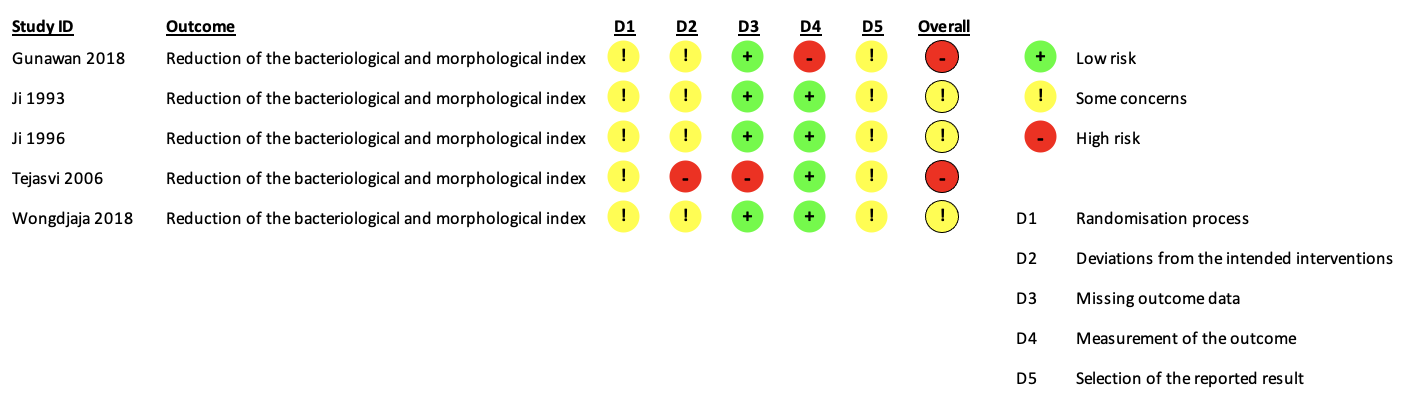
**

**Figure 8S-B.** Assessment of the risk of bias in the RCT included for the outcomes ‘reduction in the bacteriological index’ and ‘reduction in the morphological index’, performed using the RoB 2 tool.

The RoB in the studies by Gunawan et al.,(28) and Tejasvi et al.,(31) was judged as high risk of general bias, and the studies by Ji et al.,(29) and Wongdjaja et al.,(32) were judged as presenting some concern regarding the adverse event outcome (Figure 9S-B).

**
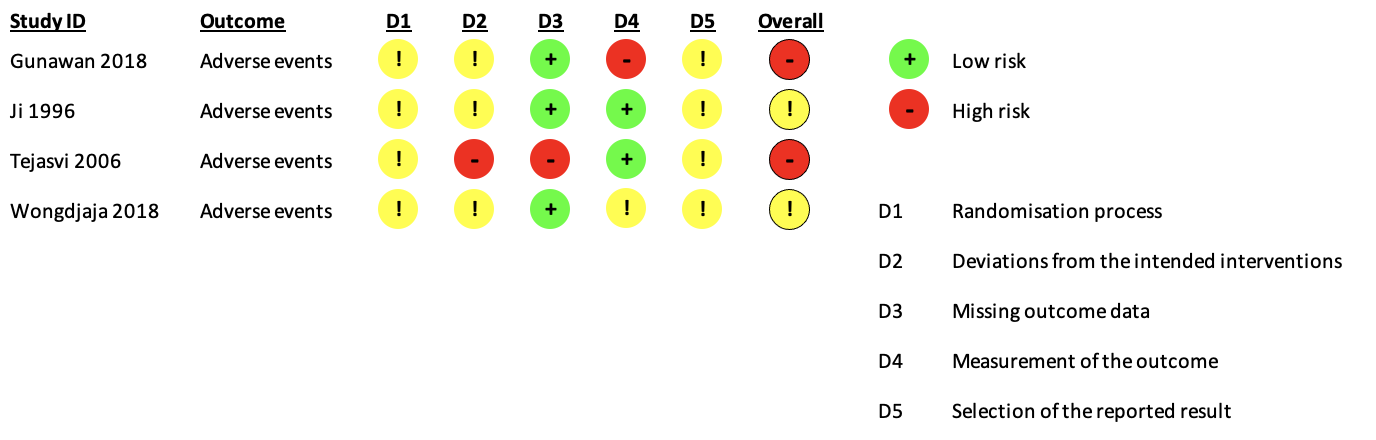
**

**Figure 9S-B.** Risk of bias assessment of the included RCT for the adverse event outcome performed using the RoB 2 tool.

**SECONDARY OUTCOMES (CLARITHROMYCIN REVIEW)**

**Reduction in the bacteriological and morphological index**

The outcomes of bacteriological and morphological index reduction were evaluated in the Gunawan et al.,(28) Ji et al.,(29,30) Tejasvi et al.,(31) and Wongdjaja et al.,(32) studies (Tables **3S-B and 4S-B**). The study by Tejasvi et al.,(31) did not present the posttreatment mean and standard deviation, and consequently, it was not possible to present the standard deviation of each group and calculate the confidence interval of the mean difference in the treatment effect. The study showed that at baseline, the clarithromycin, rifampicin, sparfloxacin and minocycline groups had a bacteriological index mean of 3.72±1.8, and at 48 weeks, they showed a reduction of 19.17%, whereas the WHO/MDT group (MB) presented an index of 3.35±1.63 at baseline and presented a reduction of 18.87% at the same follow-up timepoint (the difference in the reduction of the bacteriological index between groups was not significant, *p value* = 0.09). Regarding the morphological index, both groups showed 100% reduction at the end of 8 weeks; however, the authors reported that there was a faster reduction in the clarithromycin, rifampicin, sparfloxacin and minocycline groups than in the WHO-MB-MDT group at four weeks (100% *vs.* 93.95%, p value = 0.001).

**Table 3S-B.** Bacteriological index postintervention of the included studies.

| **Autor, year** | **Follow-up time** | **Intervention** | **mean** | **SD** | **N** | **Comparator** | **mean** | **SD** | **N** | **MD (95% CI)^*^** | **Effect direction** |
| --- | --- | --- | --- | --- | --- | --- | --- | --- | --- | --- | --- |
| Gunawan et al. 2018 (28) | 3 months | clarithromycin dapsone and clofazimine | 2.51 | 0.80 | 7 | WHO-MB-MDT | 2.40 | 0.94 | 7 | 0.11 (-0.80 to 1.02) | No difference |
| Ji et al. 1993 (30) | 56 days | clarithromycin and minocycline | 4.88 | 0.51 | 12 | clarithromycin | 4.75 | 0.74 | 12 | 0.13 (-0.38 to 0.64) | No difference |
|  |  |  |  |  |  | minocycline | 4.68 | 0.51 | 11 | 0.20 (-0.22 to 0.62) |  |
| Ji et al. 1996 (29) | 30 days | clarithromycin and minocycline, followed by placebo | 4.18 | 0.70 | 10 | clarithromycin, minocycline and ofloxacin followed by placebo | 4.72 | 0.33 | 10 | -0.54 (-1.02 to -0.06) | Favours the intervention |
|  |  |  |  |  |  | rifampicin, clofazimine and dapsone | 4.41 | 0.67 | 10 | -0.23 (-0.83 to 0.37) | No difference |
|  |  |  |  |  |  | rifampicin followed by placebo | 4.43 | 0.67 | 10 | -0.25 (-0.85 to 0.35) | No difference |
|  |  |  |  |  |  | dapsone and clofazimine | 4.51 | 0.64 | 10 | -0.33 (-0.92 to 0.26) | No difference |
| Tejasvi et al. 2006 (31) | 48 weeks | clarithromycin, rifampicin, sparfloxacin and minocycline | 3.01 | NR | 18 | WHO-MB-MDT | 2.72 | NR | 12 | 0.29 (Not estimable) | - |
| Wongdjaja et al. 2018 (32) | 12 weeks | clarithromycin, rifampicin and ofloxacin | 1.23 | 1.48 | 13 | WHO-MB-MDT | 2.85 | 0.55 | 13 | -1.62 (-2.48 to -0.76) | Favours the intervention |

^*^MD (95% CI): mean difference (95% confidence interval), measured by *Review Manager* version 5.4 software

**Table 4S-B.** Morphological index post intervention in the included studies.

| **Autor, year** | | **Follow-up time** | | **Intervention** | | **mean** | | **SD** | | **N** | | **Comparator** | | **mean** | | **SD** | | **N** | | **MD (95% CI)^*^** | | **Effect direction** | |
| --- | --- | --- | --- | --- | --- | --- | --- | --- | --- | --- | --- | --- | --- | --- | --- | --- | --- | --- | --- | --- | --- | --- | --- |
| Gunawan et al. 2018 (28) | 3 months | | clarithromycin, dapsone and clofazimine | | 17.34 | | 10.49 | | 7 | | WHO-MB-MDT | | 32.33 | | 20.14 | | 7 | | -14.99 (-31.81 to 1.83) | | No difference | |  |
| Ji et al. 1993 (30) | 56 days | | clarithromycin and minocycline | | 0.90 | | 1.00 | | 12 | | clarithromycin | | 1.3 | | 1.6 | | 12 | | -0.40 (-1.47 to 0.67) | | No difference | |  |
|  |  |  |  |  |  |  |  |  |  |  | minocycline | | 1.6 | | 1.9 | | 11 | | -0.70 (-1.96 to 0.56) | |  |  |  |
| Ji et al. 1996 (29) | 30 days | | clarithromycin and minocycline, followed by placebo | | 1.8 | | 1.1 | | 10 | | clarithromycin, minocycline and ofloxacin followed by placebo | | 1.9 | | 1.4 | | 10 | | -0.10 (-1.20 to 1.00) | | No difference | |  |
|  |  |  |  |  |  |  |  |  |  |  | rifampicin, clofazimine and dapsone | | 1.4 | | 1.9 | | 10 | | 0.40 (-0.96 a 1.76) | | No difference | |  |
|  |  |  |  |  |  |  |  |  |  |  | rifampicin followed by placebo | | 1.0 | | 0.8 | | 10 | | -0.10 (-0.94 a 0.74) | | No difference | |  |
|  |  |  |  |  |  |  |  |  |  |  | dapsone and clofazimine | | 3.5 | | 1.8 | | 10 | | -1.70 (-3.01 a -0.39) | | Favours the intervention | |  |
| Tejasvi et al. 2006 (31) | 8 weeks | | clarithromycin, rifampicin, sparfloxacin and minocycline | | 0 | | NR | | 18 | | WHO-MB-MDT | | 0 | | NR | | 12 | | 0 (Not estimable) | | - | |  |
| Wongdjaja et al. 2018 (32) | 12 weeks | | clarithromycin, rifampicin and ofloxacin | | 0.46 | | 0.88 | | 13 | | WHO-MB-MDT | | 1.85 | | 1.77 | | 13 | | -1.39 (-2.49 a -0.32) | | Favours the intervention | |  |

^*^MD (95% CI): mean difference (95% confidence interval), measured by *Review Manager* version 5.4 software

**Safety**

Total AEs were presented in the studies by Gunawan et al.,(28) Ji et al.,(29) Tejasvi et al.,(31) and Wongdjaja et al.(32) Gunawan et al.,(28) observed that the clarithromycin, dapsone and clofazimine treatment led to nausea in two patients (28.5%), which was related to clarithromycin and showed mild severity and spontaneous disappearance, and that the WHO-MB-MDT group presented copper colouration of the skin in five patients and reddish urine in five patients (71, 4%).

Ji et al.,(29) reported that the development of the most frequent AEs were those related to the gastrointestinal system, but the researchers did not separate them by treatment group; they only reported that these AEs occurred in 17 (85%) of 20 individuals in the groups treated with clarithromycin and minocycline followed by placebo and with clarithromycin, minocycline and ofloxacin followed by placebo, as well as in two (6.7%) of 30 subjects in the other groups (the difference between the frequency of AEs in the groups using clarithromycin and minocycline was significantly greater than that in the other groups, P < 0.01). Most events were mild to moderate in intensity and occurred rapidly (between 15 min and two hours after the administration of clarithromycin and minocycline, with or without ofloxacin) and lasted no longer than a few hours. This study also identified the presence of some leprosy reactions: four cases of erythema nodosum leprosum, one reverse reaction, and one case of acute neuritis in the groups treated with clarithromycin, minocycline and ofloxacin followed by placebo; four cases of erythema nodosum leprosum and one case of acute neuritis in the rifampicin, clofazimine and dapsone groups; one reverse reaction in the group receiving rifampicin followed by placebo; and one case of erythema nodosum leprosum in the group receiving rifampicin followed by placebo.

Tejasvi et al.,(31) observed that seven (38.8%) individuals in the clarithromycin, rifampicin, sparfloxacin and minocycline groups had mild AEs, and 16 individuals developed reversible hyperpigmentation on the face and extremities due to the use of minocycline. All individuals in the WHO-MB-MDT group developed diffuse, lesional reddish-brown pigmentation associated with ichthyosis, attributed to the use of clofazimine. Regarding leprosy reactions, a type I reverse reaction was observed in three (16.6%) individuals in the clarithromycin, rifampicin, sparfloxacin and minocycline groups, and no reaction was observed in the WHO-MB-MDT group. Wongdjaja et al.,(32) reported the occurrence of a reverse reaction in both groups and two cases of erythema nodosum leprosum in the WHO-MB-MDT group.

**Quality of life**

The quality-of-life outcome was not evaluated in any of the studies included in the present systematic review.

**Treatment adherence**

The outcome ‘adherence to medication use’ was not evaluated in any of the studies included in this systematic review.

**GRADE approach**

Due to the heterogeneity of the studies in terms of treatment groups and follow-up time, an analysis of the certainty of the evidence was performed considering the outcomes of each study individually, and it was not possible to assess the downgrade of inconsistency and publication bias domains. Therefore, the analysis of certainty of evidence study by Girdhar et al.,(27) that compared the treatment groups receiving clarithromycin, rifampicin, ofloxacin and minocycline *vs.* rifampicin, ofloxacin and minocycline was considered very low for the cure outcome and low for the relapse outcome (Table 5S-B).

**Table 5S-B.** Analysis of the certainty of evidence for the comparison of clarithromycin, rifampicin, ofloxacin and minocycline *vs.* rifampicin, ofloxacin and minocycline, considering the cure and relapse outcomes.

| **Certainty assessment** | | | | | | | **№ of patients** | | **Effect** | | **Certainty** | **Importance** |
| --- | --- | --- | --- | --- | --- | --- | --- | --- | --- | --- | --- | --- |
| **№ of studies** | **Study design** | **Risk of bias** | **Inconsistence** | **Indirectness** | **Imprecision** | **Other considerations** | **Clarithromycin, rifampicin, ofloxacin and minocycline** | **Rifampicin, ofloxacin and minocycline** | **Relative (95% CI)** | **Absolute (95% CI)** |  |  |
| **Cure (follow-up: 24 months)** | | | | | | | | | | | | |
| 1 | Randomized trials | Very serious^a^ | Not serious | Not serious | serious^b^ | None | 128/140 (91.4%) | 126/135 (93.3%) | **RR 0.98** (0.92 to 1.05) | **19 fewer per 1.000** (from 75 fewer to 47 more) | ⨁◯◯◯ Very low | CRITICAL |
| **Relapse (follow-up: 24 months)** | | | | | | | | | | | | |
| 1 | Randomized trials | serious^c^ | Not serious | Not serious | serious^b^ | None | 0/140 (0.0%) | 1/135 (0.7%) | **RR 0.96** (0.06 to 15.26) | **0 fewer per 1.000** (from 7 fewer to 106 more) | ⨁⨁◯◯ Low | CRITICAL |

**CI:** Confidence interval; **RR:** Risk ratio

1. The risk of study bias for the cure outcome of the study by Girdhar et al.,(27) was considered a high risk of general bias, with some concerns in the domains ‘randomization process’ and ‘selection of reported outcomes’ and high risk of bias in the domains ‘deviation of intended interventions’, ‘missing outcome data’, and ‘outcome measurement’.
2. The sample size and the number of events were not enough to reach the threshold of optimal information size (OIS).
3. The risk of study bias for the relapse outcome of the study by Girdhar et al.,(27) was considered a high risk of general bias, with some concerns in the domains ‘randomization process’ and ‘selection of reported outcomes’ and high risk of bias in the domains ‘deviation from intended interventions’, ‘missing outcome data’, and ‘outcome measurement’.

The analysis of the certainty of evidence for the reduction in bacteriological and morphological index outcomes of the study by Gunawan et al.,(28) that compared treatment groups receiving clarithromycin, dapsone and clofazimine *vs.* WHO-MB-MDT is presented in Table 6S-B and was considered to show low certainty. It was not possible to present the certainty of the evidence for the adverse event outcome because the authors did not identify the number of individuals with an event in each treatment arm but presented the number of events, which made it impossible to calculate the relative risk effect estimate and 95% confidence interval.

**Table 6S-B.** Analysis of the certainty of evidence for the comparison of treatment groups receiving clarithromycin, dapsone and clofazimine *vs.* WHO-MB-MDT, considering a reduction in the bacteriological and morphological index as the outcome.

| **Certainty assessment** | | | | | | | **№ of patients** | | **Effect** | | **Certainty** | **Importance** |
| --- | --- | --- | --- | --- | --- | --- | --- | --- | --- | --- | --- | --- |
| **№ of studies** | **Study design** | **Risk of bias** | **Inconsistence** | **Indirectness** | **Imprecision** | **Other considerations** | **Clarithromycin dapsone and clofazimine** | **WHO-MB-MDT** | **Relative (95% CI)** | **Absolute (95% CI)** |  |  |
| **Bacteriological index reduction (follow-up: 3 months)** | | | | | | | | | | | | |
| 1 | Randomized trials | Serious^a^ | Not serious | Not serious | Serious^b^ | None | 7 | 7 | - | MD **0.11 higher** (0.8 lower to 1.02 higher) | ⨁⨁◯◯ Low | IMPORTANT |
| **Morphological index reduction (follow-up: 3 months)** | | | | | | | | | | | | |
| 1 | Randomized trials | Serious^c^ | Not serious | Not serious | Serious^b^ | None | 7 | 7 | - | MD **14.99 lower** (31.81 lower to 1.83 higher) | ⨁⨁◯◯ Low | IMPORTANT |

**CI:** Confidence interval; **MD:** Mean difference

^a^ The study by Gunawan et al.,(28) was classified as having a high risk of bias for the outcome ‘reduction in the bacteriological index’; it presented some concerns in the domains ‘randomization process’, ‘deviation from intended interventions’ and ‘selection of reported results’ and presented a high risk of bias in the domain of outcome measurement.

^b^ The confidence interval of the mean difference in the effect of the interventions was considered very wide.

^c^ The study by Gunawan et al.,(28) was classified as having a high risk of bias for the outcome ‘reduction in the morphological index’; it presented some concerns in the domains ‘randomization process’, ‘deviation from intended interventions’ and ‘selection of reported results’ and presented a high risk of bias in the domain of outcome measurement.

The analysis of cure, reduction in the bacteriological and morphological index outcomes of the study by Ji et al.,(30) that compared treatment groups receiving clarithromycin and minocycline *vs.* clarithromycin was considered low certainty of evidence (Table 7S-B).

**Table 7S-B.** Analysis of the certainty of evidence for the comparison of clarithromycin and minocycline *vs.* minocycline, considering the outcomes ‘cure’ and ‘reduction in the bacteriological and morphological index’

| **Certainty assessment** | | | | | | | **№ of patients** | | **Effect** | | **Certainty** | **Importance** |
| --- | --- | --- | --- | --- | --- | --- | --- | --- | --- | --- | --- | --- |
| **№ of studies** | **Study design** | **Risk of bias** | **Inconsistence** | **Indirectness** | **Imprecision** | **Other considerations** | **Clarithromycin and minocycline** | **Minocycline** | **Relative (95% CI)** | **Absolute (95% CI)** |  |  |
| **Cure (follow-up: 56 days)** | | | | | | | | | | | | |
| 1 | Randomized trials | Serious^a^ | Not serious | Not serious | Serious^b^ | None | 11/11 (100.0%) | 11/11 (100.0%) | **RR 1.00** (0.85 to 1.18) | **0 fewer per 1.000** (from 150 fewer to 180 more) | ⨁⨁◯◯ Low | CRITICAL |
| **Bacteriological index reduction (follow-up: 56 days)** | | | | | | | | | | | | |
| 1 | Randomized trials | Serious^c^ | Not serious | Not serious | Serious^d^ | None | 12 | 11 | - | MD **0.2 higher** (0.22 lower to 0.62 higher) | ⨁⨁◯◯ Low | IMPORTANT |
| **Morphological index reduction (follow-up: 56 days)** | | | | | | | | | | | | |
| 1 | Randomized trials | Serious^e^ | Not serious | Not serious | Serious^d^ | None | 12 | 11 | - | MD **0.7 lower** (1.96 lower to 0.56 higher) | ⨁⨁◯◯ Low | IMPORTANT |

**CI:** confidence interval; **MD:** mean difference; **RR:** risk ratio

^a^ The study by Ji et al.,(30) was considered a concern in terms of the analysis of the risk of bias considering the cure outcome. Domains considered with some concerns were the randomization process, deviations from intended interventions and selection of reported outcomes.

^b^ The sample size and the number of events are not enough to reach the threshold of optimal information size (OIS).

^c^ The study by Ji et al.,(30) was considered as presenting some concerns in the analysis of the risk of bias considering the outcome ‘reduction in the bacteriological index’. Domains considered with some concerns were the randomization process, deviations from intended interventions and selection of reported outcomes.

^d^ The confidence interval of the MD in the effects of the interventions was considered very wide.

^e^ The study by Ji et al.,(30) was considered as presenting some concerns in the analysis of the risk of bias considering the outcome ‘reduction in the morphological index’. The domains considered with some concerns were the randomization process, deviations from intended interventions and selection of reported results.

The analysis of the certainty of evidence for the cure outcome was considered very low, that for the outcome ‘reduction in the bacteriological index’ was considered moderate, and that for the outcome ‘reduction in the morphological index’ was considered low for the study by Ji et al.,(29) which compared the clarithromycin treatment groups receiving minocycline followed by placebo *vs.* clarithromycin, minocycline and ofloxacin followed by placebo (Table 8S-B). It was not possible to present the certainty of the evidence for the adverse event outcome because the authors did not identify the number of individuals with an event in each treatment arm but presented the number of events, which made it impossible to calculate the relative risk effect estimate and 95% confidence interval.

**Table 8S-B.** Analysis of the certainty of evidence for comparison of the treatment groups receiving clarithromycin, minocycline, followed by placebo *vs.* clarithromycin, minocycline and ofloxacin followed by placebo, considering the outcomes ‘cure’ and ‘reduction in the bacteriological and morphological index’.

| **Certainty assessment** | | | | | | | **№ of patients** | | **Effect** | | **Certainty** | **Importance** |
| --- | --- | --- | --- | --- | --- | --- | --- | --- | --- | --- | --- | --- |
| **№ of studies** | **Study design** | **Risk of bias** | **Inconsistence** | **Indirectness** | **Imprecision** | **Other considerations** | **Clarithromycin, minocycline, followed by placebo** | **Clarithromycin, minocycline and ofloxacin followed by placebo** | **Relative (95% CI)** | **Absolute (95% CI)** |  |  |
| **Cure (follow-up: 30 days)** | | | | | | | | | | | | |
| 1 | Randomized trials | Serious^a^ | Not serious | Not serious | Very serious^b^ | None | 3/10 (30.0%) | 2/10 (20.0%) | **RR 1.50** (0.32 to 7.14) | **100 more per 1.000** (from 136 fewer to 1.000 more) | ⨁◯◯◯ Very low | CRITICAL |
| **Bacteriological index reduction (follow-up: 30 days)** | | | | | | | | | | | | |
| 1 | Randomized trials | Serious^c^ | Not serious | Not serious | Not serious | None | 10 | 10 | - | MD **0.54 lower** (1.02 lower to 0.06 lower) | ⨁⨁⨁◯ Moderate | IMPORTANT |
| **Morphologycal index reduction (follow-up: 30 days)** | | | | | | | | | | | | |
| 1 | Randomized trials | Serious^d^ | Not serious | Not serious | Serious^e^ | None | 10 | 10 | - | MD **0.1 lower** (1.2 lower to 1 higher) | ⨁⨁◯◯ Low | IMPORTANT |

**CI:** confidence interval; **MD:** mean difference; **RR:** risk ratio

^a^ The study by Ji et al.,(29) was considered to present a concern in the analysis of the risk of bias considering the cure outcome. The domains considered with some concerns were the randomization process, deviations from intended interventions and selection of reported results.

^b^ The CI of the RR for the cure outcome comprised both a null effect and considerable risk or benefit (risk ratio ≥ 1.25 or ≤ 0.75); therefore, it was considered imprecise. In addition, the sample size and the number of events were not sufficient to reach the threshold of optimal information size (OIS).

^c^ The study by Ji et al.,(29) was considered to present some concerns in the analysis of the risk of bias considering outcome ‘reduction in the bacteriological index’. The domains considered with some concerns were the randomization process, deviations from intended interventions and selection of reported results.

^d^ The study by Ji et al.,(29) was considered to present a concern in the analysis of the risk of bias considering the outcome ‘reduction in the morphological index’. The domains considered with some concerns were the randomization process, deviations from intended interventions and selection of reported results.

^e^ The CI of MD was considered too wide and therefore inaccurate.

The analysis of certainty of the evidence for cure and reduction in the bacteriological and morphological index was considered low for the study by Ji et al.,(29) which compared the treatment groups receiving clarithromycin and minocycline followed by placebo *vs.* rifampicin, clofazimine and dapsone (Table 9S-B).

**Table 9S-B.** Analysis of certainty of evidence for comparison of the treatment groups receiving clarithromycin and minocycline followed by placebo *vs.* rifampicin, clofazimine and dapsone, considering the outcomes ‘cure’ and ‘reduction in the bacteriological and morphological index’.

| **Certainty assessment** | | | | | | | **№ of patients** | | **Effect** | | **Certainty** | **Importance** |
| --- | --- | --- | --- | --- | --- | --- | --- | --- | --- | --- | --- | --- |
| **№ of studies** | **Study design** | **Risk of bias** | **Inconsistence** | **Indirectness** | **Imprecision** | **Other considerations** | **Clarithromycin, minocycline, followed by placebo** | **Rifampicin, clofazimine and dapsone** | **Relative (95% CI)** | **Absolute (95% CI)** |  |  |
| **Cure (follow-up: 30 days)** | | | | | | | | | | | | |
| 1 | Randomized trials | Serious^a^ | Not serious | Not serious | Serious^b^ | None | 3/10 (30.0%) | 9/9 (100.0%) | **RR 0.33** (0.14 to 0.80) | **670 fewer to 1.000** (from 860 fewer to 200 fewer) | ⨁⨁◯◯ Low | CRITICAL |
| **Bacteriological index reduction (follow-up: 30 days)** | | | | | | | | | | | | |
| 1 | Randomized trials | Serious^c^ | Not serious | Not serious | Serious^d^ | None | 10 | 10 | - | MD **0.23 lower** (0.83 lower 0.37 to higher) | ⨁⨁◯◯ Low | IMPORTANT |
| **Morphological index reduction (follow-up: 30 days)** | | | | | | | | | | | | |
| 1 | Randomized trials | Serious^e^ | Not serious | Not serious | Serious^d^ | None | 10 | 10 | - | MD **0.4 higher** (0.96 lower to 1.76 to higher) | ⨁⨁◯◯ Low | IMPORTANT |

**CI:** confidence interval; **MD:** mean difference; **RR:** risk ratio

^a^ The study by Ji et al.,(29) was considered to present a concern in the analysis of the risk of bias considering the cure outcome. The domains considered with some concerns were the randomization process, deviations from intended interventions and selection of reported results.

^b^ The sample size and the number of events were not enough to reach the threshold of optimal information size (OIS).

^c^ The study by Ji et al.,(29) was considered to present a concern in the analysis of the risk of bias considering the outcome ‘reduction in the bacteriological index’. The domains considered with some concerns were the randomization process, deviations from intended interventions and selection of reported results.

^d^ The CI of MD was considered too wide and therefore inaccurate.

^e^ The study by Ji et al.,(29) was considered to present a concern in the analysis of the risk of bias considering the outcome ‘reduction in the morphological index’. The domains considered with some concerns were the randomization process, deviations from intended interventions and selection of reported results.

The certainty of the evidence from the study by Ji et al.,(29) for comparing clarithromycin and minocycline followed by placebo *vs*. rifampicin followed by placebo was considered low for all outcomes evaluated (Table 10S-B).

**Table 10S-B.** Analysis of the certainty of evidence for the comparison of treatment groups receiving clarithromycin and minocycline followed by placebo *vs.* rifampicin followed by placebo, considering the outcomes ‘cure’ and ‘reduction in the bacteriological and morphological index’.

| **Certainty assessment** | | | | | | | **№ of patients** | | **Effect** | | **Certainty** | **Importance** |
| --- | --- | --- | --- | --- | --- | --- | --- | --- | --- | --- | --- | --- |
| **№ of studies** | **Study design** | **Risk of bias** | **Inconsistence** | **Indirectness** | **Imprecision** | **Other considerations** | **Clarithromycin minocycline, followed by placebo** | **Rifampicin followed by placebo** | **Relative (95% CI)** | **Absolute (95% CI)** |  |  |
| **Cure (follow-up: 30 days)** | | | | | | | | | | | | |
| 1 | Randomized trials | Serious^a^ | Not serious | Not serious | Serious^b^ | None | 3/10 (30.0%) | 10/10 (100.0%) | **RR 0.33** (0.14 to 0.80) | **670 fewer to 1.000** (from 860 fewer to 200 fewer) | ⨁⨁◯◯ Low | CRITICAL |
| **Bacteriological index reduction (follow-up: 30 days)** | | | | | | | | | | | | |
| 1 | Randomized trials | Serious^c^ | Not serious | Not serious | Serious^d^ | None | 10 | 10 | - | MD **0.25 lower** (0.85 lower to 0.35 higher) | ⨁⨁◯◯ Low | IMPORTANT |
| **Morphological index reduction (follow-up: 30 days)** | | | | | | | | | | | | |
| 1 | Randomized trials | Serious^e^ | Not serious | Not serious | Serious^d^ | None | 10 | 10 | - | MD **0.1 lower** (0.94 lower to 0.74 higher) | ⨁⨁◯◯ Low | IMPORTANT |

**CI:** confidence interval; **MD:** mean difference; **RR:** risk ratio

^a^ The study by Ji et al.,(29) was considered to present a concern in the analysis of the risk of bias considering the cure outcome. The domains considered with some concerns were the randomization process, deviations from intended interventions and selection of reported results.

^b^ The sample size and the number of events were not enough to reach the threshold of optimal information size (OIS).

^c^ The study by Ji et al.,(29) was considered to present a concern in the analysis of the risk of bias considering the outcome ‘reduction in the bacteriological index’. The domains considered with some concerns were the randomization process, deviations from intended interventions and selection of reported results.

^d^ The CI of MD was considered too wide and therefore inaccurate.

^e^ The study by Ji et al.,(29) was considered to present a concern in the analysis of the risk of bias considering the outcome ‘reduction in the morphological index’. The domains considered with some concerns were the randomization process, deviations from intended interventions and selection of reported results.

The analysis of the certainty of evidence of the study by Ji et al.,(29) for the comparison of clarithromycin and minocycline followed by placebo *vs.* dapsone and clofazimine was considered very low for the cure outcome and low for the reduction in the bacteriological and morphological index (Table 11S-B).

**Table 11S-B.** Analysis of the certainty of evidence for comparison of treatment groups receiving clarithromycin and minocycline followed by placebo *vs.* dapsone and clofazimine, considering the outcomes ‘cure’ and ‘reduction in the bacteriological and morphological index’.

| **Certainty assessment** | | | | | | | **№ of patients** | | **Effect** | | **Certainty** | **Importance** |
| --- | --- | --- | --- | --- | --- | --- | --- | --- | --- | --- | --- | --- |
| **№ of studies** | **Study design** | **Risk of bias** | **Inconsistence** | **Indirectness** | **Imprecision** | **Other considerations** | **Clarithromycin and minocycline, followed by placebo** | **Dapsone and clofazimine** | **Relative (95% CI)** | **Absolute (95% CI)** |  |  |
| **Cure (follow-up: 30 days)** | | | | | | | | | | | | |
| 1 | Randomized trials | Serious^a^ | Not serious | Not serious | Very serious^b^ | None | 3/10 (30.0%) | 4/10 (40.0%) | **RR 0.75** (0.22 to 2.52) | **100 fewer to 1.000** (de 312 fewer to 608 more) | ⨁◯◯◯ Very low | CRITICAL |
| **Bacteriological index reduction (follow-up: 30 days)** | | | | | | | | | | | | |
| 1 | Randomized trials | Serious^c^ | Not serious | Not serious | Serious^d^ | None | 10 | 10 | - | MD **0.33 lower** (0.92 lower to 0.26 higher) | ⨁⨁◯◯ Low | IMPORTANT |
| **Morphological index reduction (follow-up: 30 days)** | | | | | | | | | | | | |
| 1 | Randomized trials | Serious^e^ | Not serious | Not serious | Serious^d^ | None | 10 | 10 | - | MD **1.7 lower** (3.01 lower to 0.39 lower) | ⨁⨁◯◯ Low | IMPORTANT |

**CI:** confidence interval; **MD:** mean difference; **RR:** risk ratio

^a^ The study by Ji et al.,(29) was considered to present a concern in the analysis of the risk of bias considering the cure outcome. The domains considered with some concerns were the randomization process, deviations from intended interventions and selection of reported results.

^b^ The CI of the RR for the cure outcome comprises both a null effect and considerable risk or benefit (risk ratio ≥ 1.25 or ≤ 0.75); therefore, it was considered imprecise. In addition, the sample size and the number of events were not sufficient to reach the threshold of optimal information size (OIS).

^c^ The study by Ji et al.,(29) was considered to present a concern in the analysis of the risk of bias considering the outcome ‘reduction in the bacteriological index’. The domains considered with some concerns were the randomization process, deviations from intended interventions and selection of reported results.

^d^ The CI of MD was considered wide and therefore inaccurate.

^e^ The study by Ji et al.,(29) was considered to present a concern in the analysis of the risk of bias considering the outcome ‘reduction in the morphological index’. The domains considered with some concerns were the randomization process, deviations from intended interventions and selection of reported results.

It was not possible to assess the certainty of the evidence of the Tejasvi et al.(31) study for the outcome ‘reduction in the bacteriological and morphological index’ because the authors did not present the standard deviation of the mean of these outcomes in the posttreatment, and it was not possible to calculate the confidence interval. However, the certainty of the evidence would have a minimum downgrade of two levels considering the RoB to be very severe, since the study presented a high risk of general bias, with some concerns in the domains ‘randomization process’ and ‘selection of reported results’ and a high RoB in the domains ‘deviation from intended interventions’ and ‘missing outcome data’. Therefore, this study would present a maximum certainty of evidence classified as low for the evaluated outcomes.

Analysis of the certainty of the evidence for the comparison of the clarithromycin, rifampicin and ofloxacin treatment *vs.* WHO-MB-MDT was considered low for the outcome ‘reduction in the bacteriological and morphological index’, as shown in Table 12S-B

**Table 12S-B.** Analysis of the certainty of evidence for comparison of treatment groups receiving clarithromycin, rifampicin and ofloxacin *vs.* WHO-MB-MDT, considering the outcome ‘reduction in the bacteriological and morphological index’.

| **Certainty assessment** | | | | | | | **№ of patients** | | **Effect** | | **Certainty** | **Importance** |
| --- | --- | --- | --- | --- | --- | --- | --- | --- | --- | --- | --- | --- |
| **№ of studies** | **Study design** | **Risk of bias** | **Inconsistence** | **Indirectness** | **Imprecision** | **Other considerations** | **Clarithromycin, rifampicin and ofloxacin** | **WHO-MB-MDT** | **Relative (95% CI)** | **Absolute (95% CI)** |  |  |
| **Bacteriological index reduction (follow-up: 12 weeks)** | | | | | | | | | | | | |
| 1 | Randomized trials | Serious^a^ | Not serious | Not serious | Serious^b^ | None | 13 | 13 | - | MD **1.62 lower** (2.48 lower to 0.76 lower) | ⨁⨁◯◯ Low | IMPORTANT |
| **Morphological index reduction (follow-up: 12 weeks)** | | | | | | | | | | | | |
| 1 | Randomized trials | Serious^c^ | Not serious | Not serious | Serious^b^ | None | 13 | 13 | - | MD **1.39 lower** (2.49 lower to 0.32 lower | ⨁⨁◯◯ Low | IMPORTANT |

**CI:** Confidence interval; **MD:** Mean difference

^a^ The study by Wongdjaja et al.,(32) was classified as having risk of bias with some concerns regarding the outcome ‘reduction in the bacteriological index’, as it presented some concerns in the domains ‘randomization process’, ‘deviation from intended interventions’ and ‘selection of reported results’.

^b^ The CI of MD was considered too wide and therefore inaccurate.

^c^ The study by Wongdjaja et al.,(32) was classified as having risk of bias with some concerns regarding the outcome ‘reduction in the morphological index’, as it presented some concerns in the domains ‘randomization process’, ‘deviation from intended interventions’ and ‘selection of reported results’.
